# Combining Anion Transport and Phospholipid Binding for Improved Antibacterial Activity of Diamidocarbazoles

**DOI:** 10.1021/acsomega.5c09348

**Published:** 2025-10-29

**Authors:** Krystyna Maslowska-Jarzyna, Emmanuel O. Ojah, Maria L. Korczak, Michał J. Chmielewski, Nathalie Busschaert

**Affiliations:** † University of Warsaw, Faculty of Chemistry, Biological and Chemical Research Centre, Żwirki i Wigury 101, Warsaw 02-089, Poland; ‡ Department of Chemistry, 5783 Tulane University, New Orleans, Louisiana 70118, United States

## Abstract

Increasing bacterial resistance necessitates focusing on new antibiotic targets, such as bacterial membranes. Herein, heteroditopic diamidocarbazole-based receptors are shown to display significant antibacterial activity against Gram-positive bacteria by combining transmembrane anion transport with phosphatidylethanolamine binding, while remaining nontoxic for human red blood cells. Liposome-based and bacterial studies consistently support this dual mode of action, highlighting the potential of combining anion transport and lipid-binding properties as a strategy for the development of novel antibiotics.

## Graphical abstract

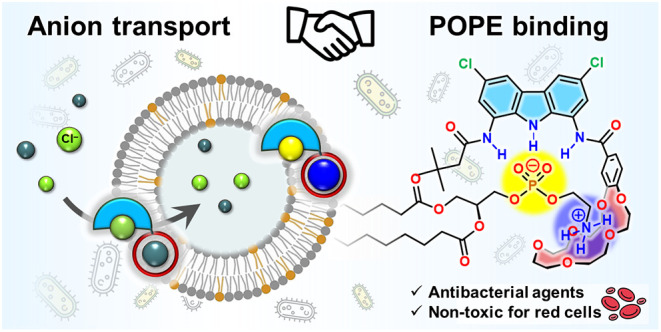

## Introduction

The rise in bacterial resistance to antibiotics poses a severe global health risk, intensified by the stagnation in the development of new antibiotic classes.^1^ Traditional antibiotics often target bacterial proteins and nucleic acids (ribosomes), leading to the rapid emergence of resistant strains. Consequently, there is an urgent need to explore alternative therapeutic targets. One promising option is the bacterial phospholipid membrane,^2^ which is crucial for maintaining cell integrity and function. The membrane can be targeted by either binding to the lipids in the membrane (e.g., antimicrobial peptides),^3^ or by increasing membrane permeability via pore formation or ionophore activity (e.g., valinomycin).^4^ Either way, selectivity can be achieved due to the different membrane composition, smaller size (and thus larger surface-area-to-volume ratio) and higher membrane potential of bacteria compared to mammalian cells.^5^

Bacterial membranes primarily consist of phospholipids such as phosphatidylethanolamine (PE), phosphatidylglycerol (PG), or cardiolipin (CL). In particular, PE is a major component of bacterial membranes of Gram-negative and certain Gram-positive strains.^6^ In contrast, phosphatidylcholine (PC) is the dominant component of mammalian cell membranes,^7^ and differs from PE in the degree of methylation of the ammonium group (Figure 1). This difference in composition creates an opportunity to develop synthetic molecules that can selectively bind to bacterial phospholipids and thereby exhibit antibacterial activity without being toxic to human cells. Busschaert and co-workers have applied this strategy to develop neutral urea-based compounds that target bacterial PE^8,9^ or PG^10^ over mammalian PC, and other research groups have also used similar lipid-targeting strategies.^11–16^ As an alternative to lipid binding, a variety of synthetic anion receptors^17^ and anion transporters (anionophores) have also been shown to possess promising antibacterial activity.^18–24^ Lipid binding has the advantage that a clear strategy for selectivity toward bacterial cells can be envisioned, but high concentrations are needed before lipid headgroup binding can lead to detrimental effects on the bacterial membrane. On the other hand, a single anionophore can transport multiple anions across the membrane, and antibacterial activity should theoretically be possible at low concentrations. However, the basis of selectivity for bacterial cells is less clear in this case, although it is likely related to the fact that fewer ions need to be transported in bacteria to induce a change in membrane potential compared with mammalian cells. We therefore wondered whether both strategies could be combined to create bifunctional molecules capable of anion transport and phospholipid binding. This could allow combining the advantages of both strategies (good selectivity and high potency) and achieving more effective antibacterial agents. While many antimicrobial peptides are thought to form pores upon lipid binding, this effect only occurs at high concentrations.^25^ The combination of lipid binding for selectivity with mobile carrier type ionophore activity (which theoretically can function at the single molecule level) has not yet been reported. In this manuscript, we report a crown ether-containing diamidocarbazole derivative that is able to function as both an ionophore and as a receptor for PE lipids in liposomes, and exerts antibacterial activity against a variety of Gram-positive bacteria with minimum inhibitory concentrations (MICs) as low as ∼4 μM and no observable hemolysis of human red blood cells.

**Figure 1:**
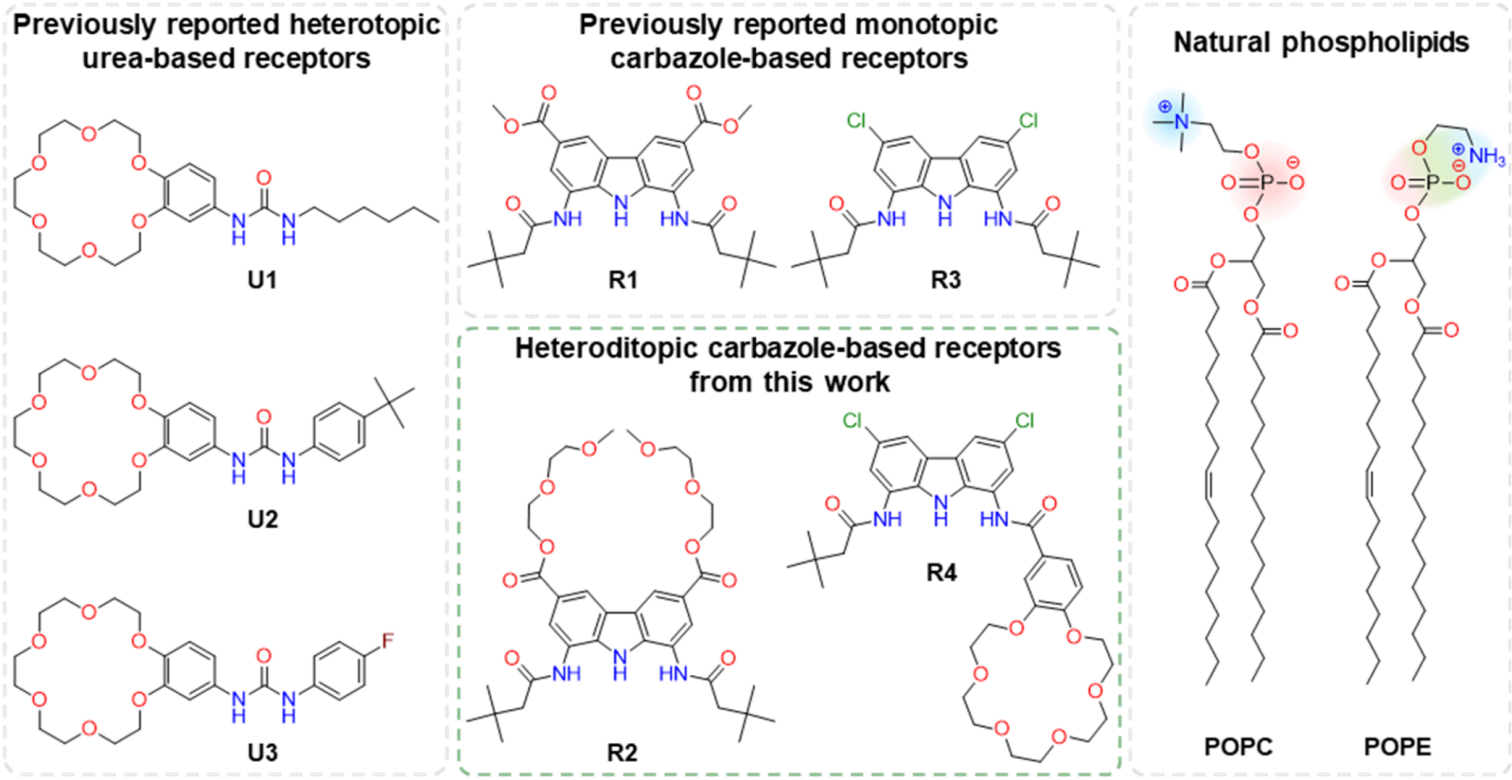
Chemical structures of urea-based receptors **U1**–**U3**, carbazole-based receptors **R1**–**R4** and natural phospholipids POPC and POPE, which were used as examples of PC and PE lipids in this work.

## Results and Discussion

### Design and Synthesis

In our previous work on PE-binding antibacterial compounds, we used a single urea as the anion-binding moiety.^8,9^ Monoureas are generally modest anion transporters,^26,27^ and within the membrane environment anion transport and lipid binding are two competing processes. Given this competition, monoureas functionalized with a crown ether, **U1**–**U3**, preferentially interact with lipid headgroups and do not promote transmembrane ion transport.^9^ To overcome this limitation and develop compounds capable of both lipid binding and efficient anion transport, we selected a more effective anionophore scaffold. Diamidocarbazoles have previously been shown to function as potent transmembrane anion transporters with antibacterial activity.^22^ Furthermore, they also exhibit high affinity for oxyanions,^28–30^ including phosphates, making them good candidates for combined ion transport and phospholipid headgroup binding. However, selective recognition of PE over PC will depend on the ability of the receptors to bind the cationic moiety of these phospholipids as well. Natural and synthetic oligoethers are known to bind ammonium cations,^31^ and we therefore designed and synthesized the first heteroditopic diamidocarbazole-based receptors **R2** and **R4** with oligoether domains for the simultaneous binding of the phosphate and ammonium moieties of the lipids, and compared their properties with model monotopic anion receptors **R1** and **R3** (Figure 1).

Two different design strategies were employed to obtain the heteroditopic receptors. For **R2**, we incorporated flexible oligoether chains at the ester groups of carbazole-based receptor **R1**,^32^ while for **R4** a benzo-18-crown-6 moiety replaced one neopentyl group of receptor **R3**. Crown ether derivatives are notable for their ability to selectively bind primary ammonium cations over substituted ammonium cations.^33^ Furthermore, macrocyclic compounds often show better binding than flexible compounds and a stronger effect of the oligoether was therefore expected for **R4** compared to **R2**. The monotopic analogues **R1** and **R3** were used as comparisons throughout this manuscript, especially because **R3** has previously been shown to exhibit antibacterial activity, likely arising from its anion transport properties.^22^ Synthetic details and characterization of **R2** and **R4** are provided in the Supporting Information.

### Phospholipid Binding Studies

Carbazole-based receptors have a strong chromophore that allows the use of UV–vis titrations to assess the affinity of **R1**–**R4** to PE and PC lipids. Titrations were performed in CHCl_3_ using palmitoyl-2-oleoyl-*sn*-glycero-3-phosphoethanol-amine (POPE) or palmitoyl-2-oleoyl-*sn*-glycero-3-phosphocholine (POPC) as model guests (Figure 2A and Supporting Information). These phospholipids do not form membranes in chloroform and remain as free molecules, allowing accurate determination of association constants. The titration data were fitted using BindFit,^34^ and the obtained binding constants are summarized in Table 1. The results show that all compounds except **R4** bind POPC with similar strength, suggesting comparable affinity to the phosphate moiety in POPC. A modest effect of the oligoether domain was observed for receptor **R2** vs **R1** (3.4-fold increase in binding), whereas a much larger effect was seen for **R4** vs **R3** (19-fold increase). Interactions between 18-crown-6 and quaternary ammonium groups have previously been observed (even though they are not as strong as the interactions with primary ammonium cations),^35,36^ and this likely accounts for the enhanced POPC binding by **R4**. Whereas all compounds showed binding to POPC, **R1**–**R3** did not show any measurable binding to POPE, while a high affinity toward POPE was observed for heteroditopic receptor **R4** (Table 1). Unlike POPC, POPE is known to adopt a “closed” conformation in both nonpolar solvents and membranes due to intramolecular hydrogen bonding (Figure 1).^37^ In this conformation, the phosphate anion is in close proximity to the ammonium cation, which makes it difficult for monotopic or flexible receptors to bind. Among all tested receptors, only **R4** binds to the POPE headgroup. This unique behavior arises from its two preorganized binding domains, which enable simultaneous binding of phosphate by the diamidocarbazole moiety and ammonium by the crown ether moiety, as supported by DFT calculations (Figure 2B).

**Figure 2:**
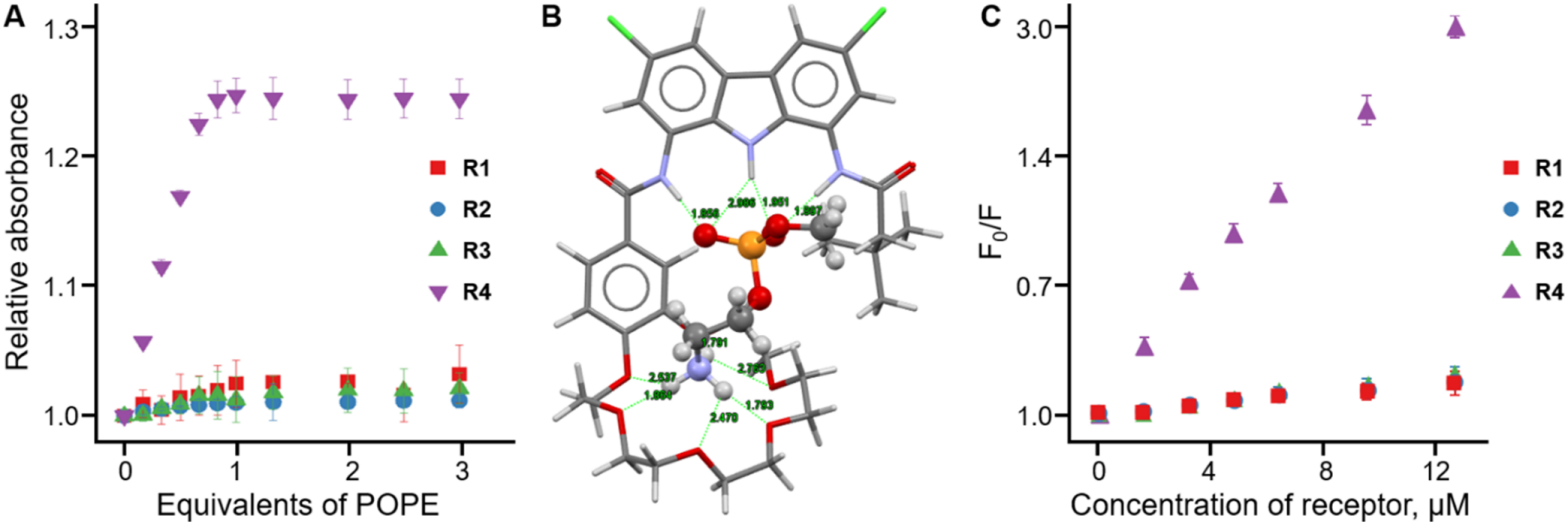
(A) UV–vis titration curves of **R1**–**R4** (5 × 10^–5^ M) with POPE in CHCl_3_ at 298 K. (B) The geometry-optimized structure of **R4** and methyl phosphoethanolamine, as model of PE. (C) Stern–Volmer plots from the fluorescence titrations of 100 nm POPE:POPC (9:1) liposomes containing 1 mol % of 18:1–06:0 NBD-PE with receptors **R1**–**R4** at 530 nm.

**Table 1:** Overview of the Phospholipid Binding Properties and Biological Activity of Receptors **R1**–**R4**

	**log** *** K*** ^a^		***K***_**SV**_,^b^ **M** ^**–1**^			**MIC, μM** ^d^				
	**POPC**	**POPE**	**NBD-PC**	**NBD-PE**	**PE/PC** **selectivity** ^c^	***B***. ***cereus***	***B***. ***subtilis***	***S***. ***aureus***	***E***. ***faecalis***	**HC_50_, μM** ^e^
**R1**	3.68 ± 0.02	weak^f^	2.47 × 10^4^	1.29 × 10^4^	0.52	>128	2–4	32	16	885 ± 30
**R2**	4.21 ± 0.13	weak^f^	2.49 × 10^4^	1.47 × 10^4^	0.59	>128	>128	>128	>128	524 ± 8
**R3**	4.50 ± 0.28	weak^f^	2.54 × 10^4^	1.75 × 10^4^	0.69	>128	2–4	32	16	602 ± 12
**R4**	5.77 ± 0.25	>7	9.06 × 10^4^	1.71 × 10^5^	1.89	4	4–8	2	4	>950^g^

a Association constant obtained through UV–vis titrations in CHCl_3_ at 298 K.

b Stern–Volmer constant obtained through titrations of **R1**–**R4** into POPC or 9:1 POPE:POPC liposomes containing NBD-labeled lipids.

c Ratio of the Stern–Volmer constants obtained with PE and PC to indicate lipid selectivity.

d Minimum inhibitory concentration (MIC) obtained using broth microdilution methods.

e Hemolytic activity of the compounds was determined by the concentration of host needed to achieve 50% hemolysis in washed single-donor human red blood cells (HC_50_).

f No significant changes in absorbance spectra were observed.

g The percentage hemolysis was still less than 50% at the highest concentration measured.

### Studies on the Interactions with Lipid Bilayers

The ability of **R1**–**R4** to interact with phospholipids within the lipid bilayers of liposomes was investigated using our previously reported NBD-based fluorescence assay (see Supporting Information for experimental details).^8^ In this assay, Stern–Volmer-type fluorescence quenching of nitrobenzoxadiazole (NBD)-labeled lipids by putative lipid binders provides information on headgroup binding. The NBD group is attached to one of the lipid tails but is known to curl up toward the lipid–water interface.^38^ As a result, differences in Stern–Volmer constant (*K*_*SV*_) between compounds reflect variations in their local concentrations at the interface, which are influenced by differences in membrane partitioning caused by lipophilicity and/or headgroup binding. The *K*_*SV*_ values were determined using 100 nm POPC liposomes containing 1 mol % of 18:1–06:0 NBD-PC (1-oleoyl-2-(6-[(7-nitro-2–1,3-benzoxadiazol-4-yl)­amino]­hexanoyl)-*sn*-glycero-3-phosphocholine) to estimate PC binding, or 9:1 POPE:POPC liposomes containing 1 mol % of 18:1–06:0 NBD-PE (1-oleoyl-2-(6-[(7-nitro-2–1,3-benzoxadiazol-4-yl)­amino]­hexanoyl)-*sn*-glycero-3-phosphoethanolamine) to estimate PE binding. The Stern–Volmer plots obtained using POPC liposomes containing NBD-PC are provided in the Supporting Information, while the results obtained using 9:1 POPE:POPC liposomes containing NBD-PE are shown in Figure 2C. All Stern–Volmer constants are summarized in Table 1.

Receptors **R1**–**R3** showed comparable Stern–Volmer constants for NBD-PC, while the Stern–Volmer constant of **R4** was 3.6 times higher, consistent with the log*K* values obtained from UV–vis titrations (Table 1). Similarly, **R1**–**R3** had only a slight effect on NBD-PE fluorescence, suggesting weak interactions with PE, again in agreement with the titration results. In contrast, addition of **R4** to POPE-containing liposomes caused pronounced quenching of the NBD fluorophore. The calculated Stern–Volmer constants reveal almost 2-fold selectivity of **R4** for POPE over POPC, whereas **R1**–**R3** display the opposite trend, favoring the more exposed phosphate group of POPC (PE/PC selectivity < 1, Table 1).

### Anion Transport Studies

Model receptor **R3** was previously shown to transport various biologically important anions via a mobile carrier mechanism,^39,40^ and to possess antibacterial activity.^22^ To evaluate the ability of the other receptors to mediate anion transport and to examine the potential of **R4** to act as a dual lipid binder and anion transporter, we used 200 nm liposomes as a model lipid bilayer system. POPC:cholesterol (7:3) liposomes were loaded with the halide-sensitive dye lucigenin and suspended in a sodium nitrate buffer (Figure 3A; see Supporting Information for experimental details).^41^ A pulse of NaCl was added to initiate chloride transport in the presence of receptors **R1**–**R4** (1 mol % with respect to lipids, Figure 3B).

**Figure 3:**
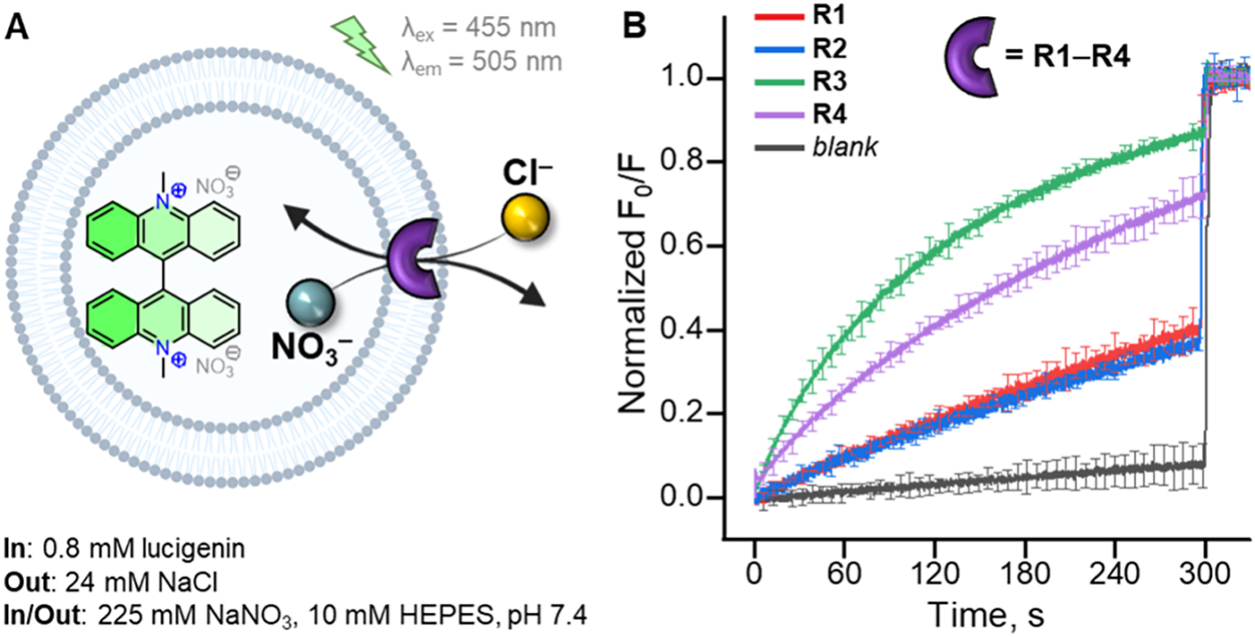
(A) Schematic representation of the lucigenin assay in POPC:cholesterol (7:3) liposomes. (B) Change in the normalized fluorescence due to the transport of Cl^–^ into 200 nm liposomes loaded with lucigenin by postinserted receptors **R1**–**R4** at 1 mol % with respect to lipids.

To quantify the Cl^–^ transport rates, we fitted the experimental fluorescence decay curves with exponential functions and compared their half-time values (*t*_1/2_, see Supporting Information). The highest transport activity was observed for **R3** (*t*_1/2_ = 89 s, Table 2), followed by **R4** (*t*_1/2_ = 176 s). The reduced activity of **R4** relative to **R3** may result from its lower lipophilicity (Table 2) and/or competition between lipid binding and anion transport. These results set **R4** apart from our previously reported urea/crown ether-based PE receptors (e.g., **U1**–**U3**), which did not show any significant anion transport activity even at 6 mol % concentration,^9^ and make it the first receptor to combine both lipid binding and anion transport functions. In contrast, compounds **R1** and **R2** exhibited only low but measurable transport rates (*t*_1/2_ = 453 and 441 s, respectively), suggesting that ester substituents are less favorable than chlorine substituents for promoting transmembrane anion transport.

**Table 2:** Half-Time Value of Anion Transport by **R1–R4**, and Calculated log *P* Values

	**Half-time** **values**, ***t***_**1/2**_ **, s** ^a^		
**Receptor**	Lucigenin assay	HPTS assay	**clog** ***P*** * ^b^
**R1**	453	58	6.27
**R2**	441	72	5.76
**R3**	89	12	7.28
**R4**	176	31	6.04

a Calculated half-time values from Cl^–^ transport experiments with postinserted receptors R1–R4 at 1 mol % with respect to lipids.

b Calculated log*P* values predicted using ChemDraw software.

Because **R2** and **R4** were designed as heteroditopic receptors, they could potentially function as M^+^/Cl^–^ symporters, where M^+^ binds to the crown ether domain and Cl^–^ binds to the diamidocarbazole unit. To test this hypothesis, the lucigenin assay was repeated in sodium sulfate buffer with pulses of NaCl, KCl, RbCl, or CsCl. Sulfate (SO_4_^2–^) is a highly hydrophilic anion that is difficult to transport; therefore, any observed Cl^–^ flux under these conditions is more likely due to M^+^/Cl^–^ symport rather than Cl^–^/SO_4_^2–^ antiport. This experiment revealed that some M^+^/Cl^–^ symport occurs with **R4** at 1 mol %, although to a smaller extent than Cl^–^/NO_3_^–^ antiport, whereas **R2** remained inactive (see Supporting Information).

Complementary transport studies using an HPTS-based assay^42^ provided additional evidence for anion transport by **R1**–**R4**. In this assay, POPC:cholesterol (7:3) liposomes were loaded with the pH-responsive fluorophore 8-hydroxypyrene-1,3,6-trisulfonate (HPTS), and a transmembrane pH gradient was generated by adding a NaOH pulse (see Supporting Information). The ability of receptors **R1**–**R4** (1 mol % with respect to lipids) to dissipate the pH gradient through H^+^/Cl^–^ symport (or the functionally equivalent OH^–^/Cl^–^ antiport) was assessed by monitoring the change in fluorescence emission ratio between the protonated and deprotonated forms of HPTS (for **R4**, Na^+^/H^+^ symport or Na^+^/OH^–^ antiport are also possible mechanisms). The fastest equilibration of pH gradient was observed for receptor **R3** (*t*_1/2_ = 12 s), which has previously been reported as an exceptionally efficient H^+^/Cl^–^ transporter under similar conditions.^22^ Importantly, our novel receptor **R4** also displayed notably rapid transport (*t*_1/2_ = 31 s), while the ester-containing **R1** and **R2** were less active (*t*_1/2_ = 58 and 72 s, respectively).

Taken together, the lucigenin and HPTS assays consistently demonstrate the superior activity of **R3**, while also revealing the unique versatility of **R4**, which can engage in multiple transport pathways. Compound **R4** is therefore a promiscuous membrane-active receptor that binds lipid headgroups (especially PE) and transports both anions and cations, with a preference for anion transport.

### Antibacterial Properties

To assess the antibacterial properties of receptors **R1**–**R4**, the minimum inhibitory concentration (MIC) was determined against a panel of bacteria strains (*B. cereus*, *B. subtilis*, *S. aureus*, *E. faecalis*, and *E. coli*) using standard broth microdilution methods.^43^ None of the compounds showed any activity against the Gram-negative bacterium *E. coli*. Although *E. coli* membranes contain approximately 75% PE,^44^ this lipid is predominantly located in the inner membrane, rendering access to this target challenging.^45^

More interesting are the results obtained with Gram-positive bacteria (Table 1). For example, *B. cereus* is a bacterium whose membrane contains large amounts of PE lipids (up to 60%),^46^ and as a result only the PE-binding receptor **R4** displayed measurable antibacterial activity against this strain. Compound **R4** completely inhibited *B. cereus* growth with an impressive MIC of 4 μM, which is much lower than that of the known PE-targeting antibiotic duramycin (MIC ∼ 32 μM).^8^ Considering that *B*. *cereus* is the closest relative to *B. anthracis* (anthrax),^47^ these results are very promising.

In contrast, both anion transporters **R1** and **R3** and the dual anion transporter and lipid binder **R4** display potent antibacterial activity against the other Gram-positive bacteria: *B. subtilis* and the potentially pathogenic^48,49^ *S. aureus* and *E. faecalis* (Table 1), which all have much lower PE concentrations than *B. cereus* (10–20% for *B. subtilis*, and essentially 0% for *S. aureus* and *E. faecalis*, see Table S1).^6^ This suggests that the high PE content in *B*. *cereus* hinders transmembrane anion transport, as was recently shown for PE-rich organelle-mimicking vesicles.^50^ As a result, the anion transporters **R1**–**R3** do not show antibacterial activity against *B. cereus*, but can still inhibit the growth of other Gram-positive bacteria where transmembrane anion transport remains feasible. In contrast, **R4**, which combines ionophoric activity with PE binding, has a broader antibacterial spectrum and is capable of inhibiting the growth of *B*. *cereus* as well as other Gram-positive bacteria.

To further confirm this hypothesis, we conducted mechanistic studies on the antibacterial activity of **R3** (anionophore only) and **R4** (dual PE binder and anionophore). These studies were performed using *B. subtilis*, because this bacterium contains intermediate levels of PE lipids, making both PE binding and anion transport plausible modes of action. A Sytox Green assay confirmed that detergent-like cell lysis or pore formation is not responsible for antibacterial activity (see Supporting Information). We next investigated the effect of the receptors on the bacterial membrane potential using the DiSC_3_(5) assay.^51^ DiSC_3_(5) is a membrane-permeable fluorescent dye that accumulates and self-quenches inside polarized cells. When the cells are depolarized, the dye leaks out into the external solution, causing an increase in fluorescence that can be measured spectroscopically. As expected, the lipid-binding receptor **R4** clearly shows membrane depolarization of *B. subtilis*, whereas the chloride transporter **R3** does not (Figure 4A). The membrane potential depends on many factors and ions,^5^ and chloride transport alone might be insufficient to cause substantial depolarization. Indeed, an influx of Cl^–^ would be expected to hyperpolarize rather than depolarize the membrane. In contrast, lipid headgroup binding can increase membrane permeability to a variety of ions, producing a more pronounced effect on membrane potential, as we have previously observed.^8,10^

**Figure 4:**
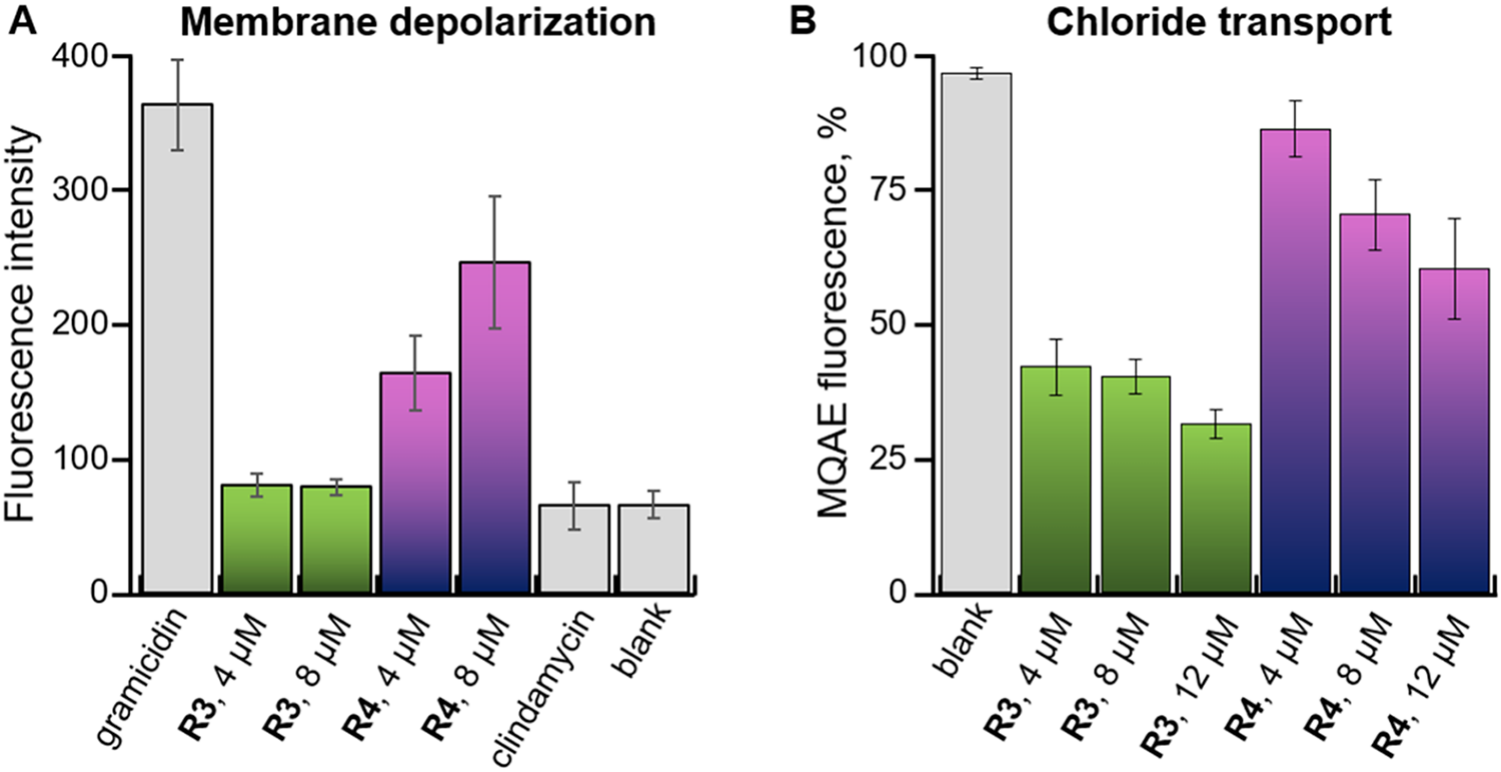
(A) Fluorescence intensity of DiSC_3_(5) in *B*. *subtilis* after 10 min incubation with gramicidin (10 μM, positive control), **R3**–**R4** at various concentrations, clindamycin (2 μg/mL, negative control), and DMSO (1%, blank). (B) Percentage fluorescence of MQAE in *B*. *subtilis* in the absence (*blank*) and presence of **R3**–**R4** at various concentrations after 5 min incubation. Results are the average of at least 2 technical × 2 biological repeats and error bars represent standard deviations.

To determine whether the anion transport activity of **R3** and **R4** alters cytosolic chloride concentrations, we employed the recently reported MQAE chloride influx assay, in which Cl^–^ transport into bacterial cells quenches MQAE fluorescence.^20^ The presumed anionophore **R3** exhibited high Cl^–^ transport activity in *B. subtilis*, whereas PE receptor **R4** showed significantly lower activity, even at high concentrations (Figure 4B). This is consistent with the liposome-based studies, which showed greater transport activity for **R3** compared to **R4**. It also supports our hypothesis that the antibacterial activity of **R3** is due to Cl^–^ transport only and therefore only works against bacteria that are sensitive to Cl^–^ transport, whereas **R4** can function as both a lipid headgroup binder and moderate anionophore and therefore has activity against a broader range of bacteria.

### Hemolysis Studies

One of the most common side effects of membrane-active compounds is the lysis of red blood cells. Red blood cells, like most mammalian cells, contain around 25% PE lipids, but this lipid is mostly located on the inner leaflet and thus not easily accessible.^52,53^ We therefore determined the concentration of **R1**–**R4** required to induce 50% hemolysis (HC_50_, Table 1) in single-donor human red blood cells, using a standard protocol.^54^ All receptors showed low toxicity at concentrations below 0.5 mM (see Supporting Information). Notably, even at concentrations approaching 1 mM, receptor **R4** caused less than 50% hemolysis, highlighting its potential as a promising antibiotic lead.

## Conclusions

In summary, we have developed the first heteroditopic molecular receptor based on a diamidocarbazole scaffold incorporating a crown ether domain, **R4**. This receptor is able to function both as an anionophore and as a selective binder for the bacterial lipid PE over the mammalian lipid PC in both solution and liposomes. Furthermore, **R4** functions as an antibacterial agent against a variety of Gram-positive bacteria, including PE-rich *B. cereus*, with MIC values below 8 μM. In contrast, receptors **R1**–**R3**, which do not interact with PE lipids, function solely as anionophores and display antibacterial activity only against strains with low PE content. Overall, our findings demonstrate that combining transmembrane anion transport and lipid headgroup binding offers a promising approach for developing next-generation membrane-targeting antibiotics, although further studies will be required to fully elucidate the mechanism of action of these antibacterial agents.

## Supplementary Material



## Notes

Synthesis and characterization of compounds **R1–R4**, UV–vis titration, fluorescence titration, anion transport data and analysis, biological and *in silico* studies (PDF)
