# Supplementary material for: Combining Anion Transport and Phospholipid Binding for Improved Antibacterial Activity of Diamidocarbazoles
Source: ACS Omega. 2025 Oct 29;10(44):53557–63. doi: 10.1021/acsomega.5c09348 (PMC12612908; doi:10.1021/acsomega.5c09348)
Supplement: Supplementary file 1 [file ao5c09348_si_001.pdf]

Supporting Information

Combining Anion Transport and Phospholipid Binding for Improved Antibacterial Activity of Diamidocarbazoles

Krystyna Maslowska-Jarzyna,[a,b] Emmanuel O. Ojah,[b] Maria L. Korczak,[a]
Michał J. Chmielewski[a*] and Nathalie Busschaert[b*]

[a] *University of Warsaw, Faculty of Chemistry, Biological and Chemical Research Centre, Żwirki i Wigury 101, 02-089 Warsaw, Poland*
[b] *Tulane University, Department of Chemistry, New Orleans, LA 70118, USA*

E-mail: mchmielewski@chem.uw.edu.pl , nbusschaert@tulane.edu

Contents

1 General information **S3**

1.1 Materials S3

1.2 Instruments and methods S4

2 Synthesis **S5**

2.1 Synthesis of receptor **R1** S6

2.2 Synthesis of receptor **R2** S6

2.3 Synthesis of receptor **R3** S11

2.4 Synthesis of receptor **R4** S12

3 Spectroscopic studies **S15**

3.1 Absorption spectra of **R1**–**R4** S15

3.2 General procedure for UV-vis titrations S15

3.3 Data fitting S15

3.4 UV-vis titrations of **R1** with POPC in organic solvent S16

3.5 UV-vis titrations of **R2** with POPC in organic solvent S18

3.6 UV-vis titrations of **R3** with POPC in organic solvent S20

3.7 UV-vis titrations of **R4** with POPC in organic solvent S22

3.8 UV-vis titrations of **R1** with POPE in organic solvent S24

3.9 UV-vis titrations of **R2** with POPE in organic solvent S25

3.10 UV-vis titrations of **R3** with POPE in organic solvent S26

3.11 UV-vis titrations of **R4** with POPE in organic solvent S27

3.12 Fluorescence titrations in POPC and POPE:POPC liposomes S28

3.13 Determination of Stern-Volmer constants S29

3.14 Fluorescence titration of POPC liposomes with **R1**–**R4** S29

3.15 Fluorescence titration of POPE:POPC 9:1 liposomes with **R1**–**R4** S31

4 Anion transport studies **S33**

4.1 General procedure for measuring anion transport s33

4.2 Chloride transport by **R1**–**R4** S33

4.3 Quantification of the transport rate S34

4.4 Determination of chloride transport rate S34

4.5 Symport assay S35

4.6 HPTS assay S36

4.7 Equilibration of pH gradient by **R1**–**R4** S36

4.8 Determination of transport rate S37

5 Biological studies **S38**

5.1 Lipid composition of bacteria used S38

5.2 MIC determination S38

5.3 Sytox Green assay S39

5.4 Membrane depolarization assay S40

5.5 MQAE chloride influx assay S41

5.6 Hemolytic activity of **R1**–**R4** S42

6 *In silico* studies **S47**

1 General information

1.1 Materials

All solvents and reagents were commercially available and used as received unless otherwise stated.

Ambeed: 4-carboxybenzo-18-crown-6 ether ($C_{17}H_{24}O_8$, 97%, A878274).

Avanti Polar Lipids: POPC (1-palmitoyl-2-oleoyl-glycero-3-phosphocholine, >99%, 850457C), POPE (1-palmitoyl-2-oleoyl-*sn*-glycero-3-phosphoethanolamine, >99%, 850757C), NBD-PC (1-oleoyl-2-(6-((7-nitro-2-1,3-benzoxadiazol-4-yl)amino)hexanoyl)-*sn*-glycero-3-phosphocholine, >99%, 810132C), NBD-PE (1-oleoyl-2-(6-((7-nitro-2-1,3-benzoxadiazol-4-yl)amino)hexanoyl)-*sn*-glycero-3-phosphoethanolamine, >99%, 810155C).

Deutero: dimethyl sulfoxide-d_6 (DMSO-d_6; 99.8% D).

Euriso-top: DMSO-d_6 + 0.03% TMS v/v (> 99.8% D), chloroform-*d* ($CDCl_3$; 99.80% D, D007HAG).

Fisher Scientific: chloroform ($CHCl_3$, ≥99.8%, C2984).

Linegal Chemicals: dichloromethane (DCM; pure p.a., 50-8124.4), freshly distilled, ethyl acetate, ($C_4H_8O_2$; 50-451000) dried and freshly distilled, hexane fraction from kerosene (C_6H_{14}; 50-1009) freshly distilled.

POCH S.A.: sulfuric acid (H_2SO_4; min. 95%, pure p.a., BA5000115), potassium hydroxide (KOH; 85%, pure p.a. basic, BA6800113), triethylamine anhydrous (TEA; pure p.a., 848930117), diethyl ether ($C_4H_{10}O$; 99.5%, pure p.a.-basic, stab. with BHT, BA4210114), tetrahydrofuran (THF; pure p.a.-basic, BA8200118).

Sigma-Aldrich: acetone (puriss. p.a., ≥99.5% (GC), 32201), acetonitrile (ACN; ≥99.9%, 34998), 3,3-dimethylbutyryl chloride (*tert*-butylacetyl chloride; 99%, B88802), diethylene glycol methyl ether ($C_5H_{12}O_3$; ≥99.0%, 579548), dimethyl sulfoxide (DMSO; anhydrous, ≥99.9%, 276855), methanol (MeOH; HPLC grade, ≥99.9%, 34860), thionyl chloride ($SOCl_2$; ≥99%, 230464), toluene (anhydrous, 99.8%, 244511).

VWR: dimethylformamide (DMF; for HPLC, 83635320, >99.90%).

1.2 Instruments and methods

Nuclear magnetic resonance (NMR) spectroscopy

NMR spectra were recorded using Bruker AM-500 (^{1}H: 500 MHz, ^{13}C: 126 MHz) spectrometer at ambient temperature in $(CD_3)_2SO$ or $CDCl_3$. The chemical shifts, δ, are reported in parts per million (ppm) and coupling constants, J, are given in hertz (Hz). The NMR spectra were referenced to the solvent residual signal (^{1}H: δ_{DMSO} = 2.500 ppm, $\delta_{chloroform}$ = 7.260 ppm, ^{13}C: δ_{DMSO} = 39.50 ppm, $\delta_{chloroform}$ = 77.16 ppm). Data are reported as follows: chemical shift, multiplicity (s – singlet, bs – broad singlet, d – doublet, t – triplet, dd – doublet of doublets, etc.), coupling constant and integration.

Mass spectrometry

The ESI-MS spectra were obtained using API 3000 (Applied Biosystems) and AutoSpec Premier (Waters) mass spectrometers with methanol as the spray solvent. Low resolution electron spray ionization mass spectra were recorded on a Bruker micrOTOF.

Elemental analysis

Elemental analysis was performed using an UNIcube elementar analyser from Elementar.

Thin layer chromatography (TLC)

TLC was carried out on Merck silica gel 60 F_{254} plates.

Preparative chromatography

Preparative chromatography was done manually on Merck silica gel 60 (230-400 mesh) or with the aid of Teledyne ISCO CombiFlash instrument using RediSep normal-phase silica flash columns.

2 Synthesis

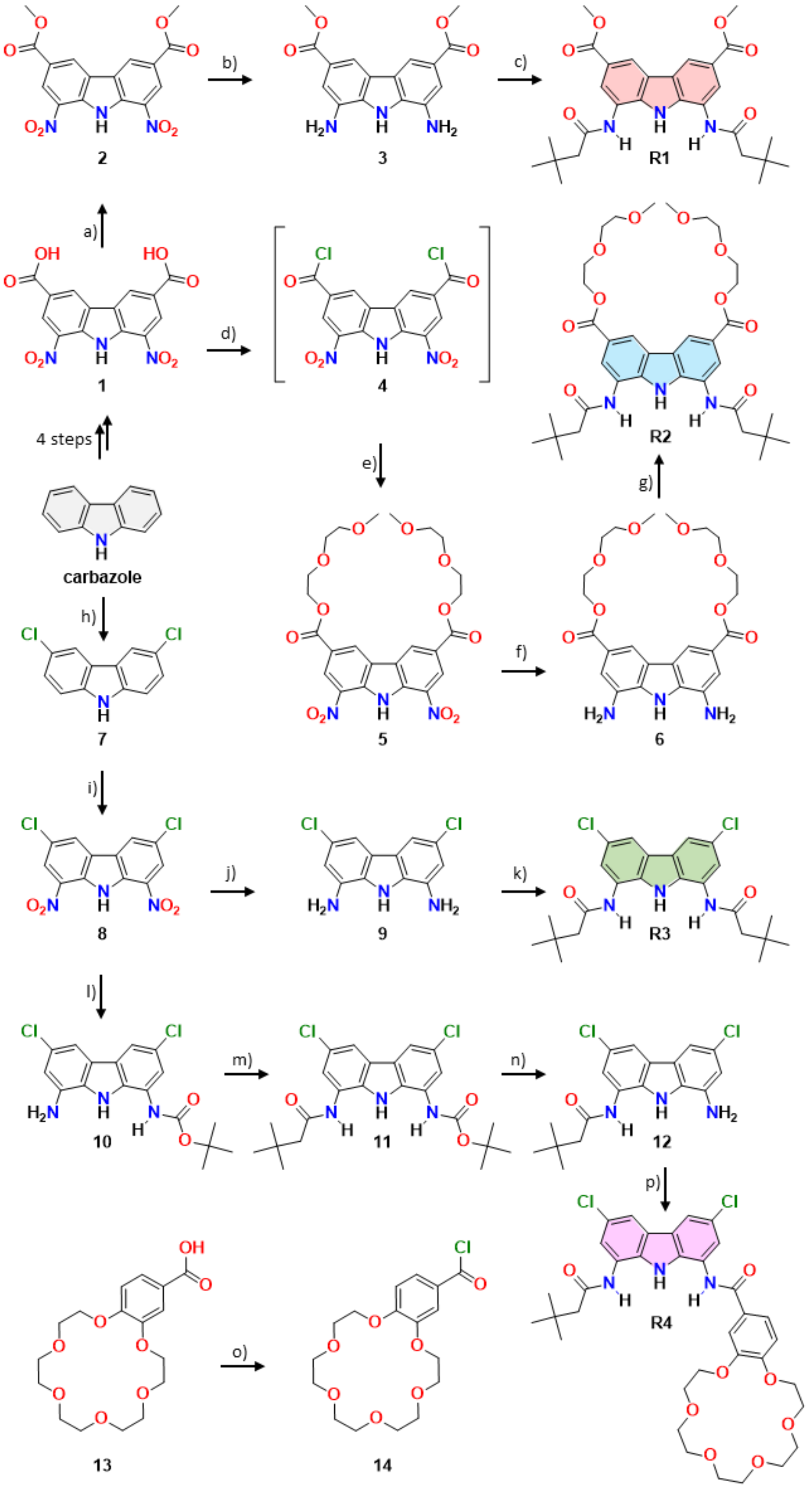

Scheme S1. Reagents and conditions. **R1**: a) MeOH, 95% H_2SO_4 (cat.), reflux, 24 h, 78%; b) H_2, 0.3 bar, 5% Pd/C, ACN, rt, 4 h, 93%; c) $(CH_3)_3CH_2COCl$, TEA, THF, rt, 1 h, 75%. **R2**: d) $SOCl_2$, DMF, 60°C; e) 2-(2-ethoxyethoxy)ethanol, DCM, TEA, 87%; f) H_2, 0.3 bar, 5% Pd/C, ACN, rt, 4 h, 97%; g) $(CH_3)_3CH_2COCl$, TEA, THF, rt, 1 h, 97%. **R3**: h) SO_2Cl_2, CH_2Cl_2, rt, 60%; i) HNO_3 (100%), Ac_2O/AcOH, 1–110 °C, 73%; j) H_2 (balloon), 5% Pt(S)/C (cat.), CH_3CN, RT, 90%; k) $(CH_3)_3CH_2COCl$, TEA, CH_3CN, rt, 88%. **R4**: l) Boc_2O, THF, reflux, 4 h, 54%; m) $(CH_3)_3CH_2COCl$, THF, rt, 1 h, 83%; m) CF_3COOH, DCM, rt, 2 h, 75%; o) $SOCl_2$, reflux, 22 h, 90%; p) **14**, THF, TEA, rt, 22 h, 75%.

2.1 Synthesis of receptor **R1**

Receptor **R1** was obtained according to literature procedure[1] as shown in Scheme S2.

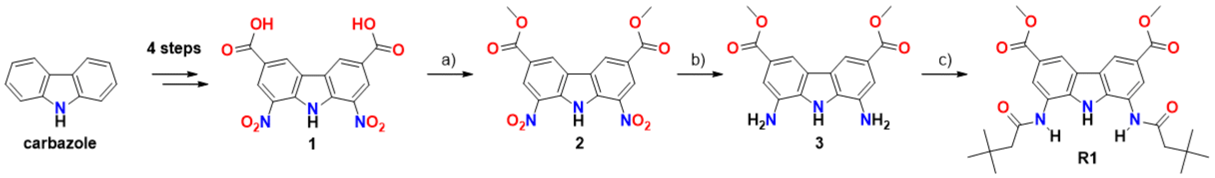

Scheme S2. Synthesis of receptor **R1**. Reagents and conditions: a) MeOH, 95% H_2SO_4 (cat.), reflux, 24 h, 78%; b) H_2, 0.3 bar, 5% Pd/C, ACN, rt, 4 h, 93%; c) $(CH_3)_3CH_2COCl$, TEA, THF, rt, 1 h, 75%.

2.2 Synthesis of receptor **R2**

Receptor **R2** was obtained as shown in Scheme S3.

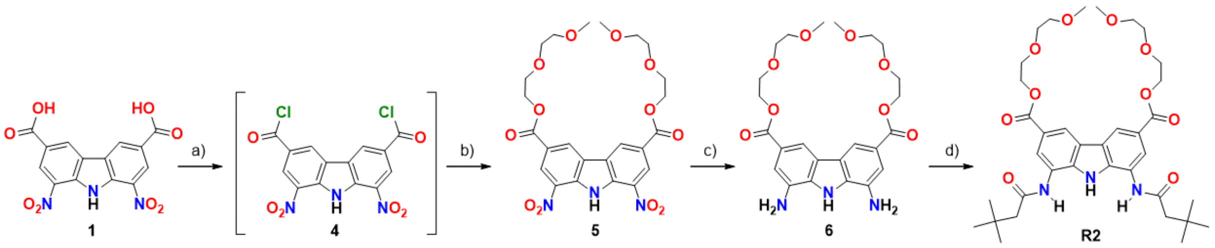

Scheme S3. Synthesis of receptor **R2**. Reagents and conditions: a) $SOCl_2$, DMF, 60°C; b) 2-(2-ethoxyethoxy)ethanol, DCM, TEA, 87%; c) H_2, 0.3 bar, 5% Pd/C, ACN, rt, 4 h, 97%; d) $(CH_3)_3CH_2COCl$, TEA, THF, rt, 1 h, 97%.

Synthesis of bis(2-(2-methoxyethoxy)ethyl) 1,8-dinitro-9H-carbazole-3,6-dicarboxylate, 5

A 10 mL single-neck round-bottom flask was charged with diacid **1**[1] (347 mg, 1.00 mmol) and equipped with a stirring bar. The flask was sealed with a septum and deaerated by sequentially evacuating and refilling with argon three times. Thionyl chloride (3 mL) and three droplets of DMF were added in the flow of argon. Then, still in the flow of argon, the flask was equipped with a reflux condenser connected to a bubbler and the reaction mixture was heated to 60°C (temperature measured in oil bath) with intense stirring for 4 h. After this time the volatiles were evaporated on a rotary evaporator and the solid residue **4** was dried further under high vacuum. Next, the flask was sealed with a septum and deaerated as before. DCM (3 mL) was added through the septum and the flask was put into an ultrasonic bath for approx. 1 minute. Triethylamine (0.40 mL) and bis(2-(2-methoxyethoxy)ethyl alcohol (0.35 mL, 2.98 mmol) were added. The reaction mixture was intensively stirred for 4 h. Then the volatiles were removed on a rotary evaporator and the solid residue was purified by column chromatography using CombiFlash instrument, 4 g cartridge, and eluent's flow of 15 mL/min. Separation was achieved using gradient elution with ethyl acetate in hexane: 0 ⟶ 25% (5 min); 25% (10 min); 25 ⟶ 50% (5 min); 50% (60 min); 50 ⟶ 100% (10 min). Fractions containing pure product were combined and evaporated on a rotary evaporator. The solid was dried in a desiccator with KOH under high vacuum. Yield: 476 mg (87%) of **5** as a yellow solid.

^{1}H NMR (500 MHz, $CDCl_3$) δ_{CDCl3}: 11.71 (s, 1H); 9.20 (dd, J_1 = 1.5 Hz; J_2 = 0.5 Hz, 2H); 9.17 (d, J = 1.5 Hz; 2H); 4.63 (m, 4H); 3.97 (m, 4H); 3.75 (m, 4H); 3.61 (m, 4H); 3.41 (s, 6H).

[1] M. L. Korczak, K. Maslowska-Jarzyna, M. J. Chmielewski, *RSC Adv.*, **2024**, *14*, 29883–29889.

^{13}C NMR (126 MHz, $CDCl_3$) δ_{CDCl3}: 164.57, 136.23, 132.95, 129.33, 126.06, 125.51, 124.35, 71.95, 70.64, 69.13, 65.03, 59.15.

Elemental analysis: calcd. for $C_{24}H_{27}N_3O_{12}$: C, 52.46; H, 4.95; N, 7.65, found: C, 52.33; H, 4.93; N, 7.65.

HR MS (TOF MS ES$^-$) m/z calcd. for $C_{24}H_{27}N_3O_{12}Na$: 572.1492 found: 572.1481.

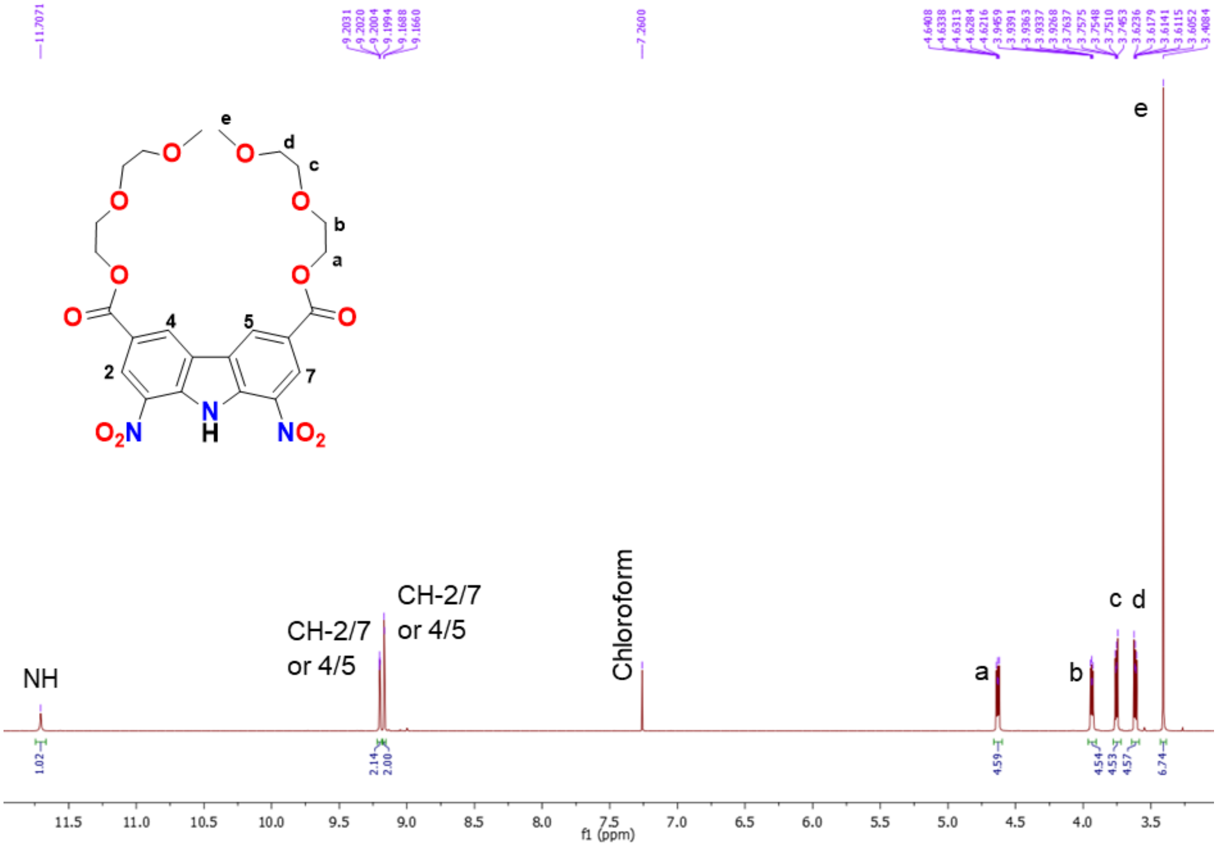

Figure S1. ^{1}H NMR spectrum of **5** in $CDCl_3$.

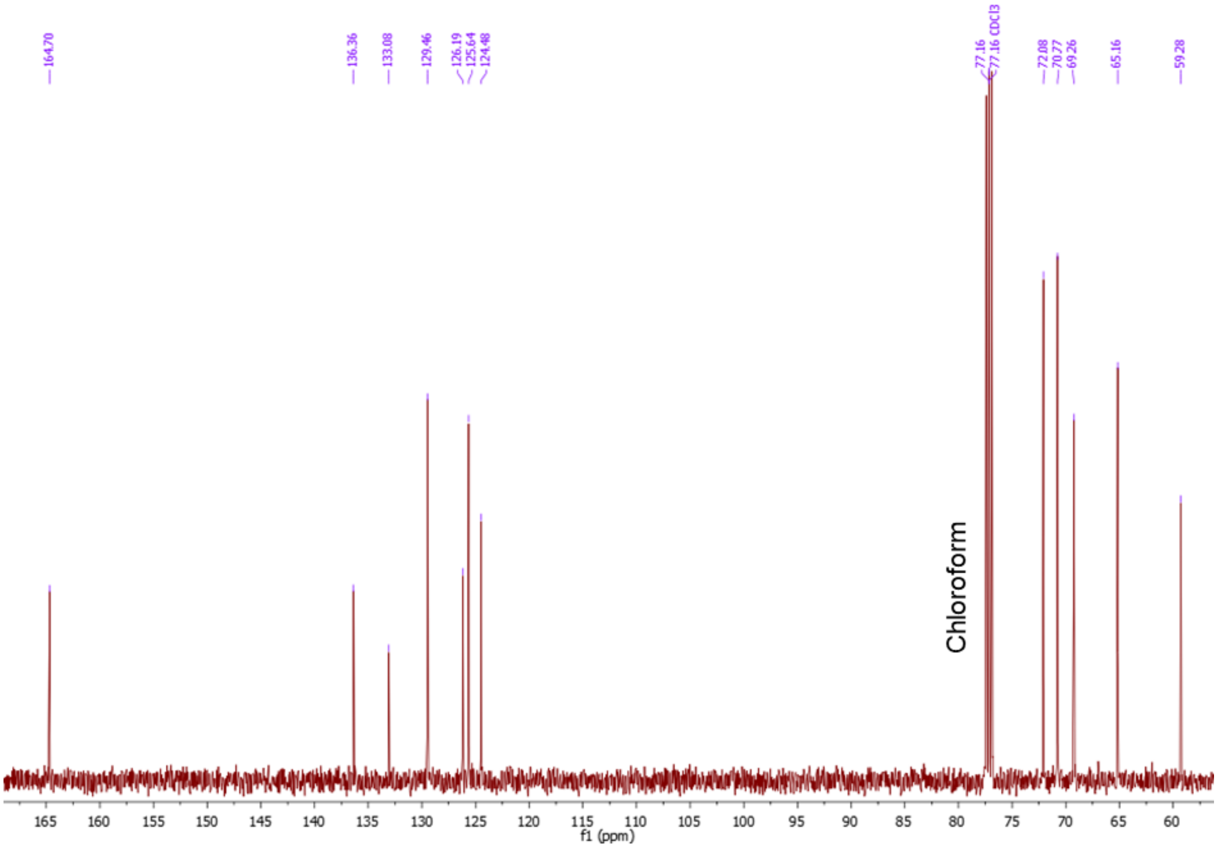

Figure S2. ^{13}C NMR spectrum of **5** in $CDCl_3$.

Synthesis of bis(2-(2-methoxyethoxy)ethyl) 1,8-diamino-9H-carbazole-3,6-dicarboxylate, 6

A 50 mL two-neck round-bottom flask was charged with dinitro compound **5** (551 mg, 1.00 mmol) and equipped with a stirring bar. The flask was closed with septa and deaerated by sequentially evacuating and refilling with argon three times. Then, acetonitrile (10 mL) and 10% Pd/C (30.1 mg) were added in a flow of argon. The argon flow was replaced with hydrogen and maintained for 30 minutes. To wash out the compounds deposited on the flask walls due to bubbling, another 10 mL of acetonitrile was added. The pressure of hydrogen was set up to 0.3 bar and the mixture was intensively stirred for 3 hours. The reaction mixture was filtered using 0.2 µm PTFE filter under argon atmosphere, and evaporated on a rotary evaporator. The solid residue was dried in a desiccator with KOH under high vacuum. Yield 475 mg (97%) of **6** as a pale pink solid.

^{1}H NMR (500 MHz, DMSO-d_6) δ_{DMSO}: 11.16 (s, 1H); 8.05 (d, J = 1.5 Hz, 2H); 7.36 (d, J = 1.5 Hz; 2H); 5.35 (s, 4H); 4.38 (m, 4H); 3.77 (m, 4H); 3.62 (m, 4H); 3.48 (m, 4H); 3.26 (s, 6H).

^{13}C NMR (126 MHz, DMSO-d_6) δ_{DMSO}: 166.78, 133.81, 132.11, 123.04, 121.81, 111.49, 110.02, 71.29, 69.69, 68.57, 63.51, 58.10.

Elemental analysis: calcd. for $C_{24}H_{31}N_3O_8$: C, 58.89; H, 6.38; N, 8.58, found: C, 58.70; H, 6.32; N, 8.59.

HR MS (TOF MS ES$^-$) m/z calcd. for $C_{24}H_{31}N_3O_8Na$: 512.2009, found: 512.1996.

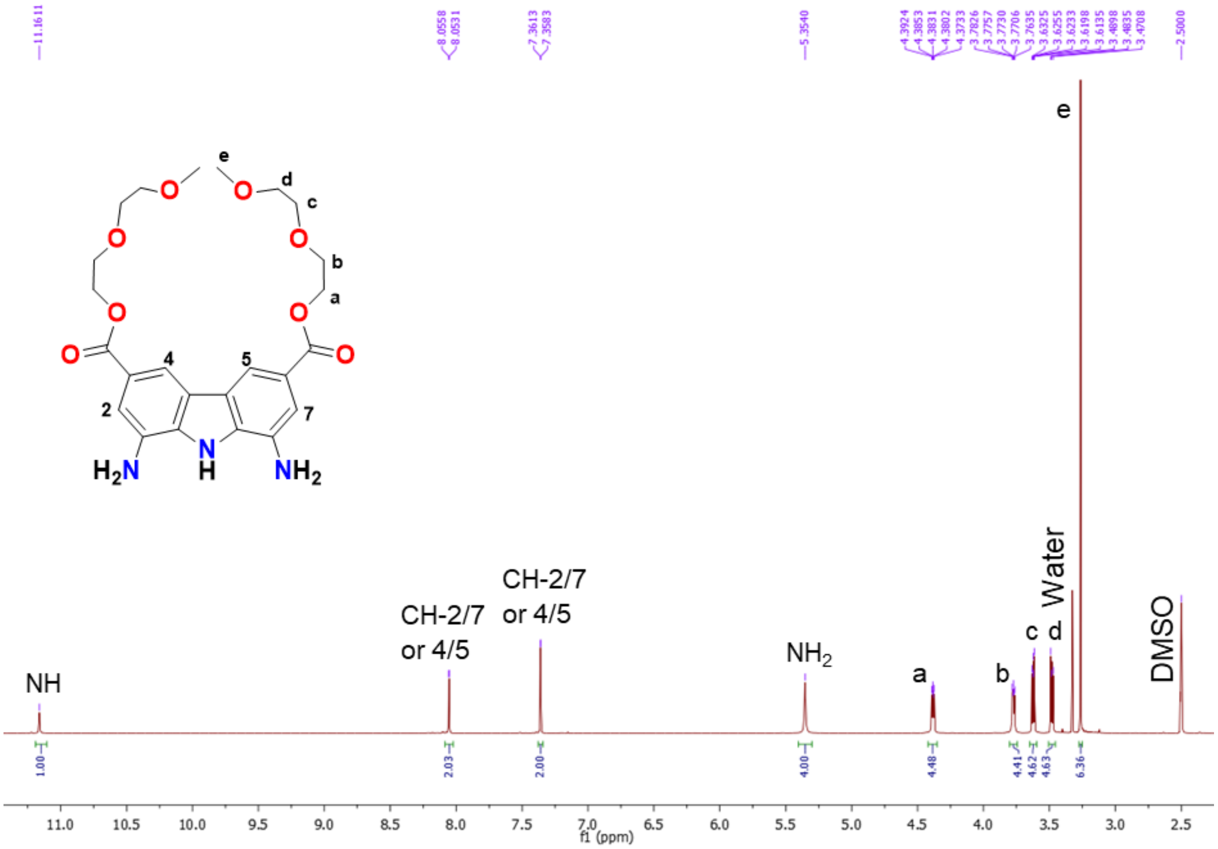

Figure S3. ^{1}H NMR spectrum of **6** in DMSO-d_6.

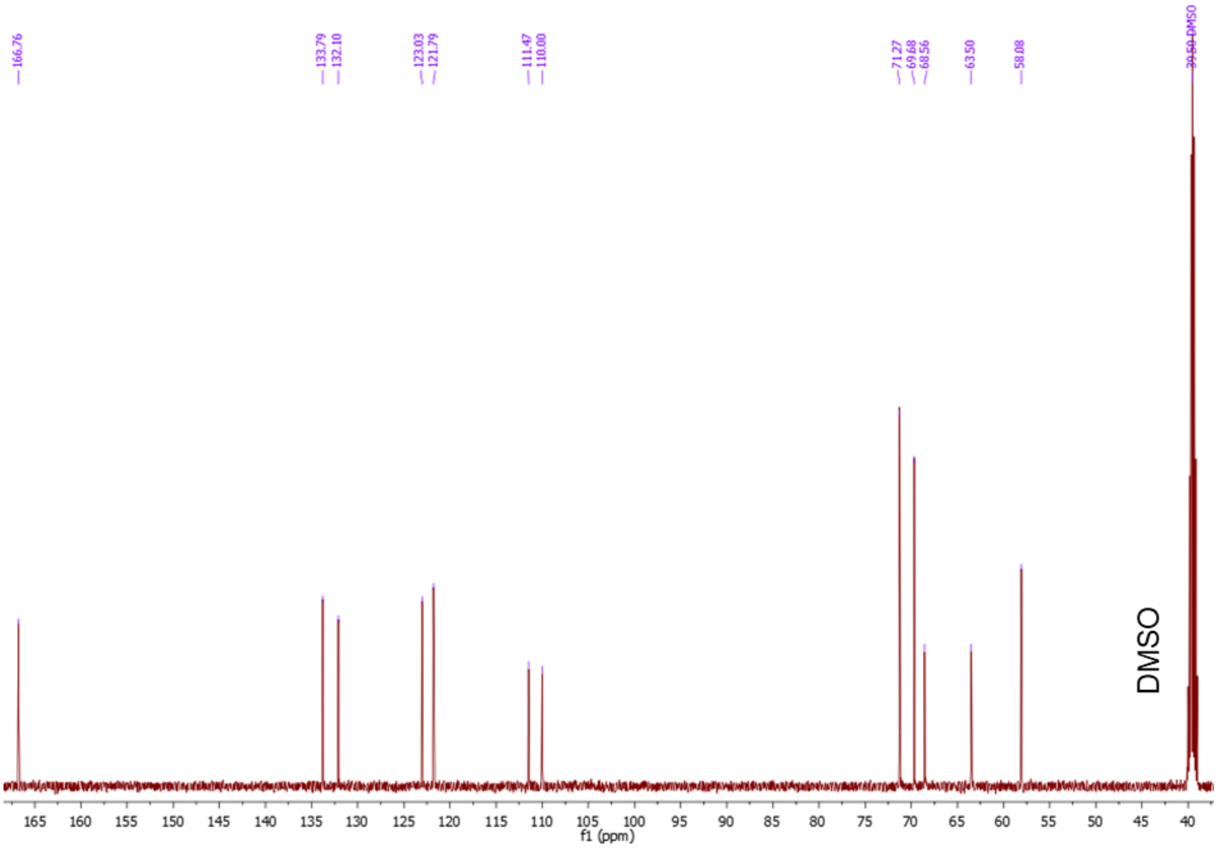

Figure S4. ^{13}C NMR spectrum of **6** in DMSO-d_6.

Synthesis of R2

A 50 mL single-neck round-bottom flask was charged with diamine **6** (147 mg, 0.300 mmol) and equipped with a stirring bar. The flask was sealed with a septum and deaerated by sequentially evacuating and refilling with argon three times. Then, dry THF (20 mL) was added, followed by TEA (0.09 mL, 67 mg, 0.66 mmol) and acid chloride (0.09 mL, 89 mg, 0.66 mmol). The reaction mixture was intensively stirred for 1 h. After this time the volatiles were evaporated on a rotary evaporator. The solid was washed with water (3 × 5 mL) on a Schott filter funnel G4. The solid was dried in a desiccator with KOH under high vacuum. Yield: 200 mg (97%) of **R2** as a white solid.

^{1}H NMR (500 MHz, DMSO-d_6) δ_{DMSO}: 10.90 (s, 1H); 10.23 (s, 2H); 8.71 (d, *J* = 1.5 Hz, 2H); 8.14 (m, 2H); 4.45 (m, 4H); 3.80 (m, 4H); 3.63 (m, 4H); 3.48 (m, 4H); 3.26 (s, 6H); 2.37 (s, 4H); 1.11 (s, 18H).

^{13}C NMR (126 MHz, DMSO-d_6) δ_{DMSO}: 170.54, 165.93, 135.46, 124.14, 123.35, 121.64, 120.47, 118.97, 71.29, 69.70, 68.48, 63.95, 58.09, 49.02, 30.93, 29.68.

HR MS (TOF MS ES$^-$) m/z calcd. for $C_{36}H_{51}N_3O_{10}Na$: 708.3472 found: 708.3484.

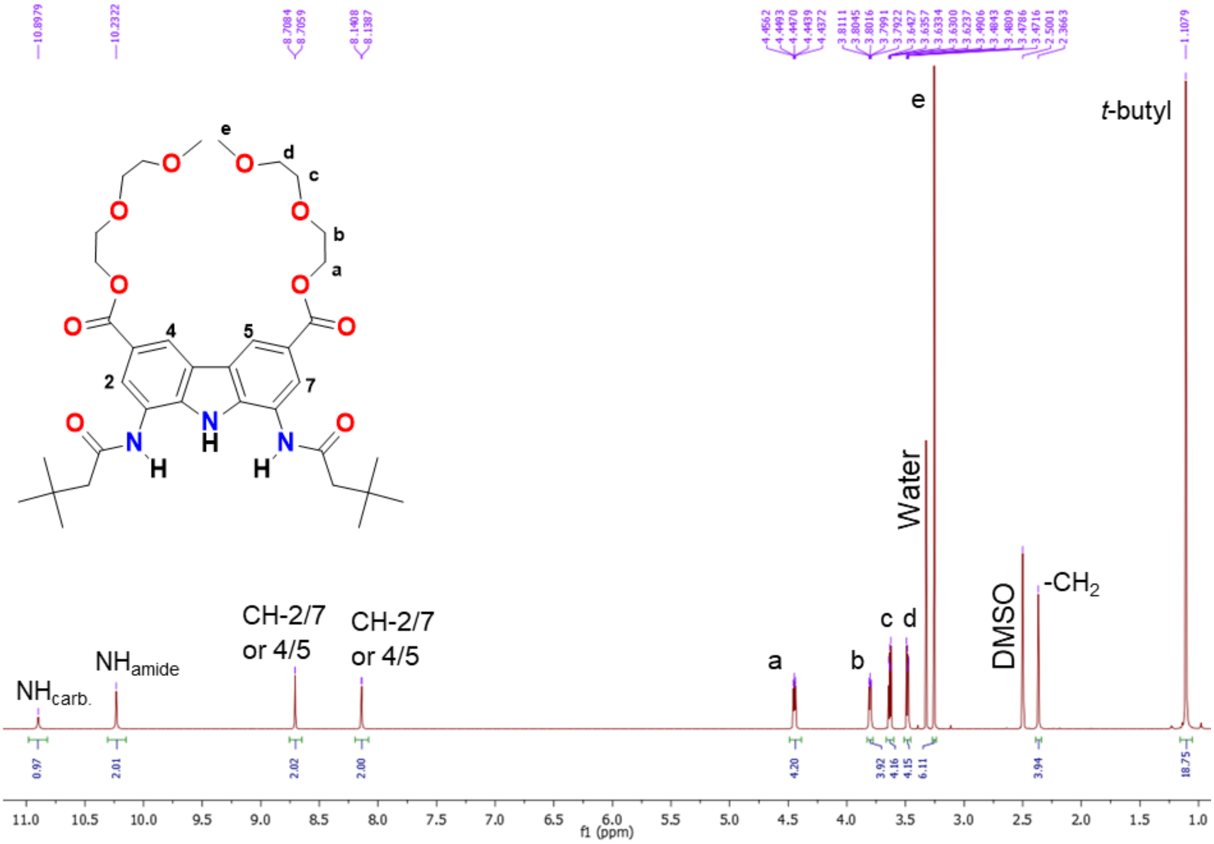

Figure S5. ^{1}H NMR spectrum of **R2** in DMSO-d_6.

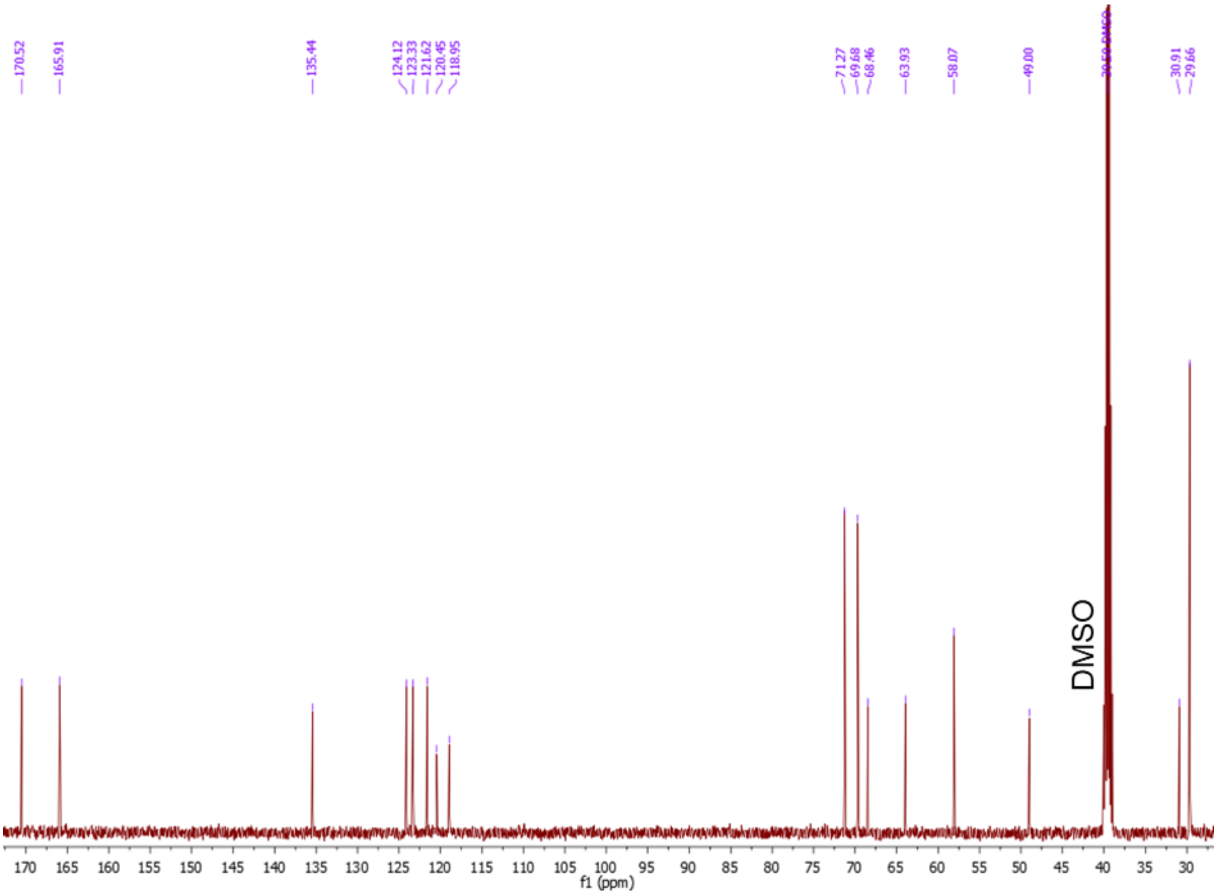

Figure S6. ^{13}C NMR spectrum of **R2** in DMSO-d_6.

2.3 Synthesis of receptor **R3**

Receptor **R3** was obtained according to literature procedure,[2] as shown in Scheme S4.

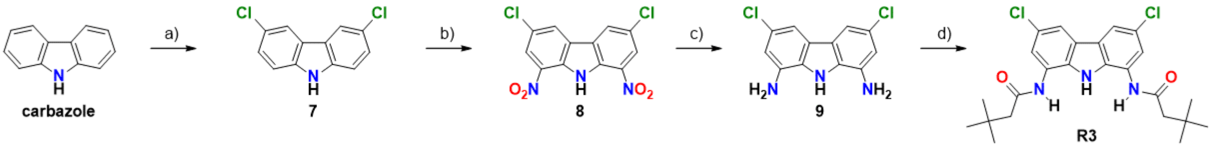

Scheme S4. Synthesis of receptor **R3**. Reagents and conditions: a) SO_2Cl_2, CH_2Cl_2, rt, 60%; b) HNO_3 (100%), Ac_2O/AcOH, 1-110 °C, 73%; c) H_2 (balloon), 5% Pt(S)/C (cat.), CH_3CN, RT, 90%; d) $(CH_3)_3CH_2COCl$, TEA, CH_3CN, rt, 88%.

[2] K. M. Bąk, K. Chabuda, H. Montes, R. Quesada and M. J. Chmielewski, *Org. Biomol. Chem.*, **2018**, *16,* 5188–5196.

2.4 Synthesis of receptor **R4**

Receptor **R4** was obtained as shown in Scheme S5. Steps a–c were carried out as reported previously.[3] Step d was carried out according to the literature.[4]

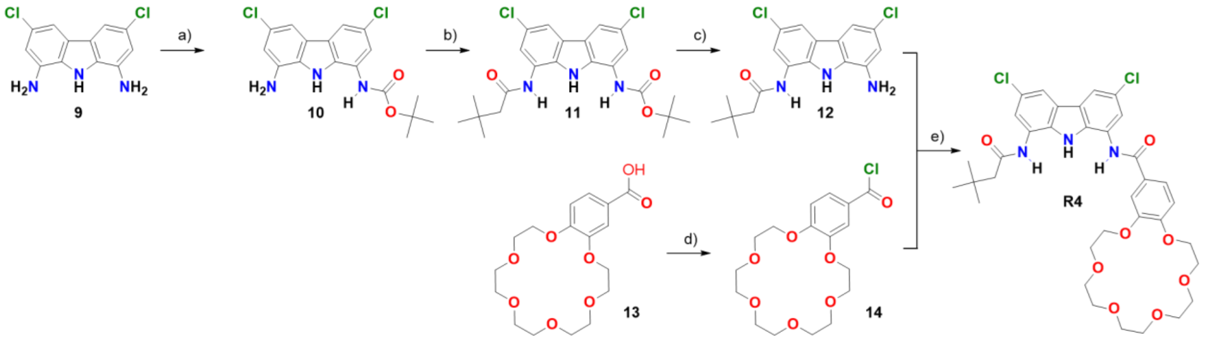

Scheme S5. Synthesis of receptor **R4**. Reagents and conditions: a) Boc_2O, THF, reflux, 4 h, 54%; b) $(CH_3)_3CH_2COCl$, THF, rt, 1 h, 83%; c) CF_3COOH, DCM, rt, 2 h, 75%; d) $SOCl_2$, reflux, 22 h, 90%; e) THF, TEA, rt, 22 h, 75%.

Synthesis of (benzo-18-crown-6)-4'-carbonyl chloride, 14

To a 25 mL round bottom flask equipped with a magnetic stirrer, 4-carboxybenzo-18-crown-6 ether **13** (180 mg, 0.5 mmol) was added, followed thionyl chloride (8 mL). The system was refluxed at 80 °C for 22 h. Excess of $SOCl_2$ was removed in vacuum after dilution with toluene (40 mL), giving 168 mg of white crystalline acyl chloride **14** (90% yield).

Synthesis of R4

The flasks used in the following syntheses were dried in furnace, and cooled down to RT in a desiccator. *Solution A.* A 25 mL single-neck round-bottom flask was charged with **12** (0.055 g, 0.15 mmol) and equipped with a stirring bar. The flask was sealed with septum and filled with nitrogen by three pump-thaw cycles. Dry THF (4 mL) was added to the flask via a syringe, followed by triethylamine (50 µL, 0.40 mmol). *Solution B.* A 50 mL round-bottom flask was charged with solid acyl chloride **14** (115 mg, 0.30 mmol), equipped with a stir bar, closed with septum and filled with nitrogen by three pump-thaw cycles. Next, dry THF (1 mL) was added via a syringe. The *Solution A* was taken via a syringe and slowly added to the *Solution B* drop by drop. White precipitate formed after the addition of the first few droplets. The mixture was intensively stirred for 22 h at room temperature. The progress of the reaction can be monitored by TLC chromatography in 7% MeOH/DCM. Next, the reaction was quenched by the addition of water (20 mL). The flask was put into a refrigerator for 1 hour. After this time, the precipitate was filtered off, washed with water (2 × 5 mL) and ethyl acetate (3 × 3 mL), and dried in a desiccator with KOH under high vacuum. Yield 79 mg (75%) of white solid.

[3] K. M. Bąk, K. Masłowska and M. J. Chmielewski, *Org. Biomol. Chem.*, **2017**, *15*, 5968–5975.
[4] J. C.S. Filho, T. C.O. M. Leod, M. C. A.F. Gotardo and M. das D. Assis, ARKIVOC, **2010**, *5*, 105–116.

^{1}H NMR (500 MHz, DMSO-d_6) δ_{DMSO}: 10.47 (s, 1H); 10.45 (s, 1H); 10.02 (s, 1H), 8.20 (d, J = 2.0 Hz, 1H), 8.11 (d, J = 2.0 Hz, 1H), 7.91 (d, J = 2.0 Hz, 1H), 7.73 (dd, J = 8.5 Hz, 2.1 Hz, 1H), 7.68 (d, J = 2.0 Hz, 1H), 7.57 (d, J = 2.0 Hz, 1H), 7.16 (d, J = 8.5 Hz, 1H), 4.20 (s, 4H), 3.80 (s, 4H), 3.63 (s, 4H), 3.57 (s, 4H), 3.54 (s, 4H), 2.31 (s, 2H), 1.06 (s, 9H).

^{13}C NMR (126 MHz, DMSO-d_6) δ_{DMSO}: 170.41, 164.93, 151.43, 147.66, 132.60, 130.18, 125.80, 124.45, 124.43, 124.17, 124.07, 123.42, 123.19, 121.68, 121.41, 118.03, 117.26, 115.92, 112.34, 112.01, 69.91, 69.79, 69.72, 69.70, 68.61, 68.54, 68.24, 49.18, 30.89, 29.58.

MS (ESI) m/z calcd. for $C_{35}H_{45}Cl_2N_5O_8$: 719.26 found: 719.00.

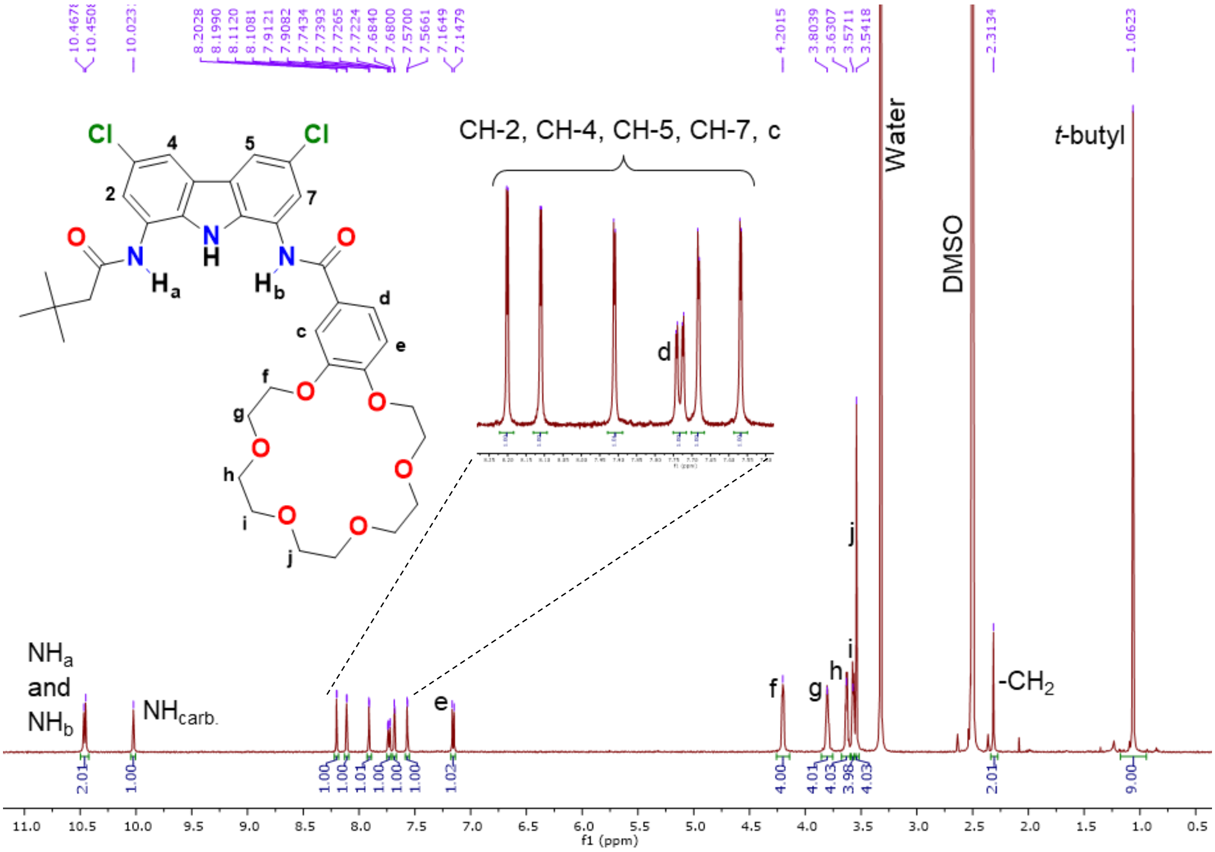

Figure S7. ^{1}H NMR spectrum of **R4** in DMSO-d_6.

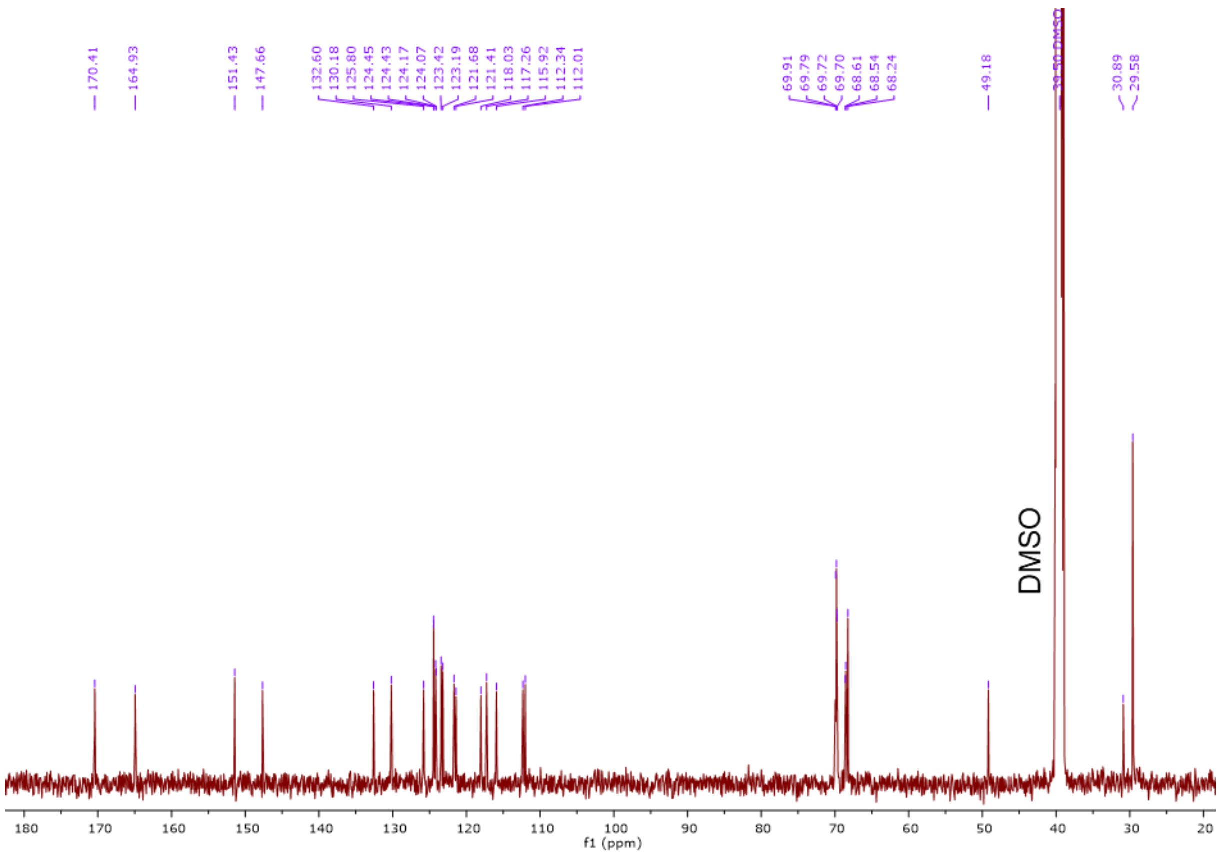

Figure S8. ^{13}C NMR spectrum of **R4** in DMSO-d_6.

3 Spectroscopic studies

3.1 Absorption spectra of **R1**–**R4**

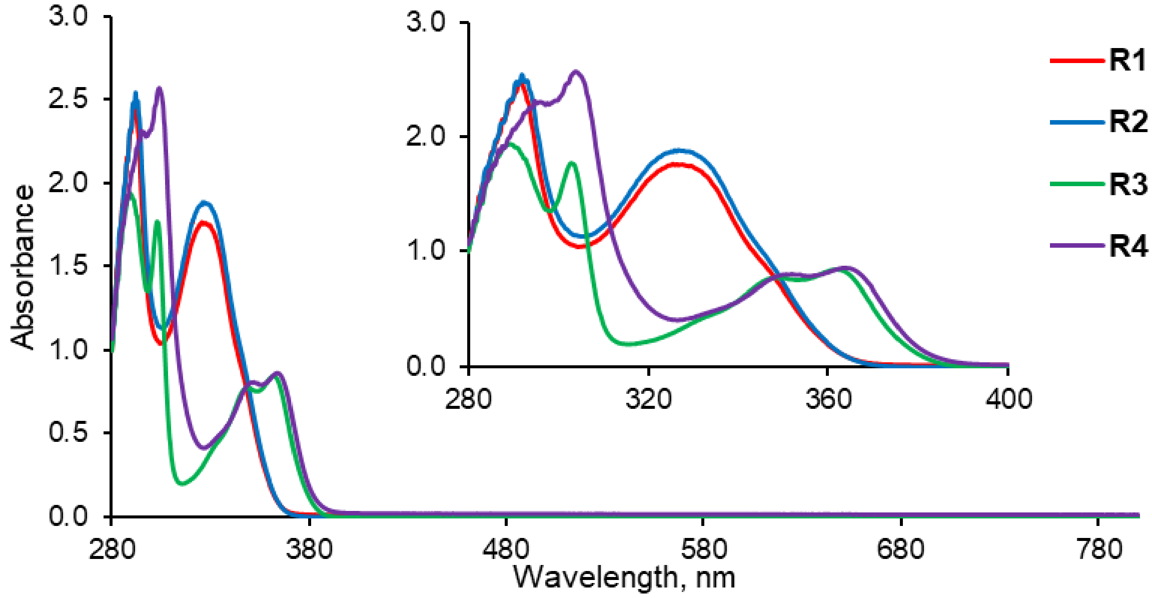

Figure S9. UV-Vis spectra of 1.2 × 10^{-4} M solution of receptors **R1**–**R4** in DMSO.

3.2 General procedure for UV-vis titrations

Preparation of standard solutions. All the reagents were weighted separately on a Mettler Toledo Excellence XA105DU analytical balance (readability 0.01 mg) in screw-capped vials sealed with Teflon-covered septa. All the solvent/solution manipulations were done using Hamilton gas-tight syringes.

UV-vis titration procedure. To a solution of host (2 mL, 5 × 10^{-5} M) in deacidified chloroform (≥99.8%, Fisher Scientific, C2984) in a quartz cuvette (optical path length: 10 mm) appropriate aliquots of titrant were added with a 10 µL gas-tight microsyringe. UV-vis spectra were recorded on Agilent Cary 100 UV-vis spectrophotometer equipped with stirring function and Peltier temperature controller.

3.3 Data fitting

The UV-vis titration data were fitted with BindFit software.[5] For each titration five wavelengths were chosen in the region of the highest absolute absorbance change, as specified in each case below. Association constants derived from independent experiments were averaged using arithmetic mean.

[5] http://supramolecular.org; a) P. Thordarson, *Chem. Soc. Rev.,* **2011**, *40*, 1305–1323; b) D. B. Hibbert and P. Thordarson, *Chem. Commun.*, **2016**, *52*, 12792–12805.

3.4 UV-vis titrations of **R1** with POPC in organic solvent

Stack of UV-vis spectra from the titration of R1 with POPC in $CHCl_3$

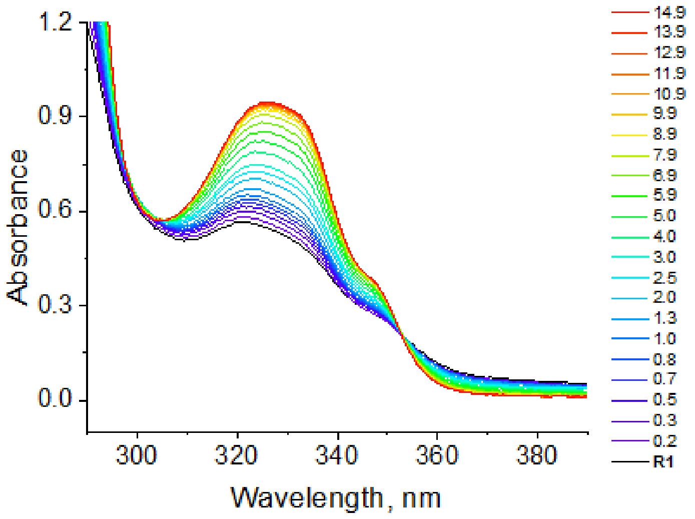

Figure S10. Stack of UV-vis spectra obtained during the titration of 5 × 10^{-5} M solution of receptor **R1** in $CHCl_3$ with 0.033 M solution of POPC (dissolved in $CHCl_3$).

Raw Data

Added volume of POPC, µl	Equivalents of POPC	Wavelength, nm				
		332	331	330	329	328
0.0	0.00	0.499594	0.506491	0.517721	0.525527	0.532812
0.5	0.17	0.516005	0.524652	0.532934	0.541956	0.549844
1.0	0.33	0.533175	0.544340	0.550958	0.563395	0.570330
1.5	0.50	0.547375	0.559875	0.567893	0.576790	0.585000
2.0	0.66	0.562927	0.571180	0.583029	0.593649	0.600120
2.5	0.83	0.575797	0.585653	0.595663	0.604291	0.612978
3.0	0.99	0.588879	0.598458	0.609044	0.617976	0.626009
4.0	1.32	0.611102	0.621649	0.630637	0.637804	0.646952
6.0	1.98	0.645746	0.656027	0.666833	0.675733	0.682916
7.5	2.48	0.673229	0.684463	0.690770	0.701996	0.708568
9.0	2.97	0.694240	0.707770	0.714420	0.725432	0.731589
12.0	3.96	0.737617	0.748400	0.756050	0.766233	0.775436
15.0	4.95	0.775318	0.785610	0.794358	0.804387	0.811107
18.0	5.94	0.809632	0.819576	0.826230	0.837104	0.841372
21.0	6.93	0.838691	0.848048	0.857099	0.867877	0.873275
24.0	7.92	0.864577	0.877126	0.886220	0.893967	0.898209
27.0	8.91	0.881089	0.891654	0.898318	0.910696	0.916868
30.0	9.90	0.890307	0.903204	0.908303	0.918924	0.925224
33.0	10.89	0.899038	0.909622	0.917351	0.923923	0.928160
36.0	11.88	0.900340	0.915488	0.919486	0.927213	0.933854
39.0	12.87	0.906235	0.917185	0.922313	0.932005	0.938042
42.0	13.86	0.908505	0.919583	0.926682	0.933068	0.936383
45.0	14.85	0.91065	0.921913	0.928872	0.935774	0.940114

Exemplary UV-vis titration curve (absorbance at 330 nm)

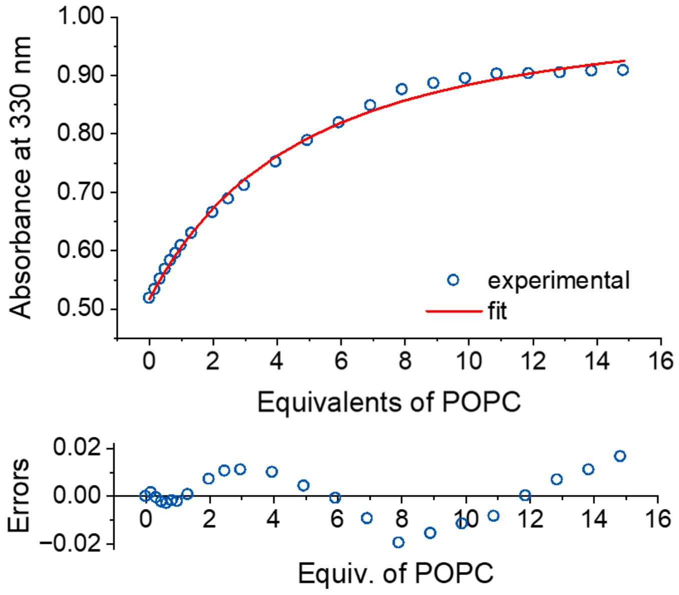

Figure S11. Fitting of 1:1 (receptor:lipid) binding model to the results of the titration of 5 × 10^{-5} M solution of receptor **R1** in $CHCl_3$ with 0.033 M solution of POPC (dissolved in $CHCl_3$).

a) Binding constant K derived from simultaneous fitting of 1:1 model to the five selected wavelengths using BindFit:

log K = 3.6705

b) Binding constant K derived from the experiment repeated according to the same methodology:

log K = 3.7034

c) Binding constant K derived from the experiment repeated according to the same methodology:

log K = 3.6768

d) Binding constant averaged from the three experiments:

log K = 3.68 ± 0.02

3.5 UV-vis titrations of **R2** with POPC in organic solvent

Stack of UV-vis spectra from the titration of R2 with POPC in $CHCl_3$

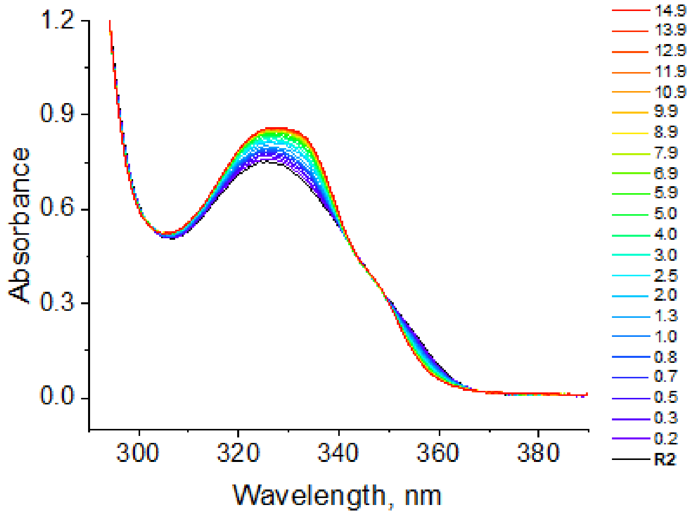

Figure S12. Stack of UV-vis spectra obtained during the titration of 5 × 10^{-5} M solution of receptor **R2** in $CHCl_3$ with 0.033 M solution of POPC (dissolved in $CHCl_3$).

Raw Data

Added volume of POPC, µl	Equivalents of POPC	Wavelength, nm				
		336	335	334	333	332
0.0	0.00	0.632715	0.651848	0.669838	0.688514	0.702648
0.5	0.17	0.639831	0.662164	0.681363	0.697307	0.714714
1.0	0.33	0.649495	0.669679	0.690658	0.708145	0.725357
1.5	0.50	0.656365	0.681057	0.700402	0.717590	0.732707
2.0	0.66	0.663836	0.685828	0.711155	0.726416	0.742227
2.5	0.83	0.669858	0.693594	0.713737	0.731710	0.750205
3.0	0.99	0.675461	0.699904	0.720556	0.737444	0.751876
4.0	1.32	0.681595	0.707035	0.732201	0.748306	0.763724
6.0	1.98	0.694381	0.722137	0.746747	0.764622	0.779615
7.5	2.48	0.703848	0.731032	0.754265	0.773155	0.787902
9.0	2.97	0.707540	0.738328	0.761741	0.779969	0.795819
12.0	3.96	0.718217	0.749156	0.773124	0.792047	0.805726
15.0	4.95	0.728060	0.758231	0.781850	0.798027	0.814103
18.0	5.94	0.732043	0.764161	0.791121	0.804115	0.818733
21.0	6.93	0.737816	0.769762	0.795208	0.811779	0.823182
24.0	7.92	0.741854	0.774312	0.799999	0.817365	0.828127
27.0	8.91	0.746538	0.778420	0.801351	0.820259	0.831870
30.0	9.90	0.749281	0.781373	0.806116	0.822154	0.835568
33.0	10.89	0.750840	0.785596	0.809342	0.825708	0.838196
36.0	11.88	0.755148	0.784871	0.811581	0.828616	0.838769
39.0	12.87	0.756342	0.790438	0.810104	0.828598	0.839217
42.0	13.86	0.758387	0.789975	0.814771	0.831965	0.841951
45.0	14.85	0.757546	0.792620	0.815292	0.832676	0.844871

Exemplary UV-vis titration curve (absorbance at 334 nm)

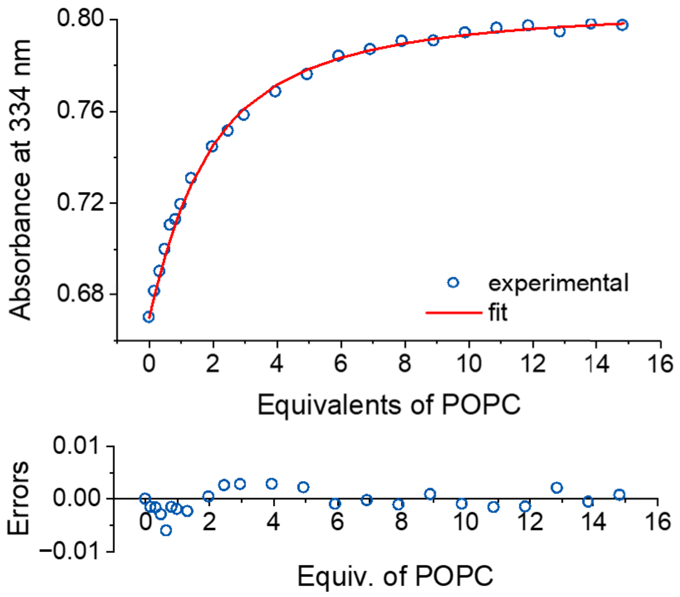

Figure S13. Fitting of 1:1 (receptor:lipid) binding model to the results of the titration of 5 × 10^{-5} M solution of receptor **R2** in $CHCl_3$ with 0.033 M solution of POPC (dissolved in $CHCl_3$).

a) Binding constant K derived from simultaneous fitting of 1:1 model to the five selected wavelengths using BindFit:

log K = 4.1799

b) Binding constant K derived from the experiment repeated according to the same methodology:

log K = 4.3463

c) Binding constant K derived from the experiment repeated according to the same methodology:

log K = 4.1027

d) Binding constant averaged from the three experiments:

log K = 4.21 ± 0.12

3.6 UV-vis titrations of **R3** with POPC in organic solvent

Stack of UV-vis spectra from the titration of R3 with POPC in $CHCl_3$

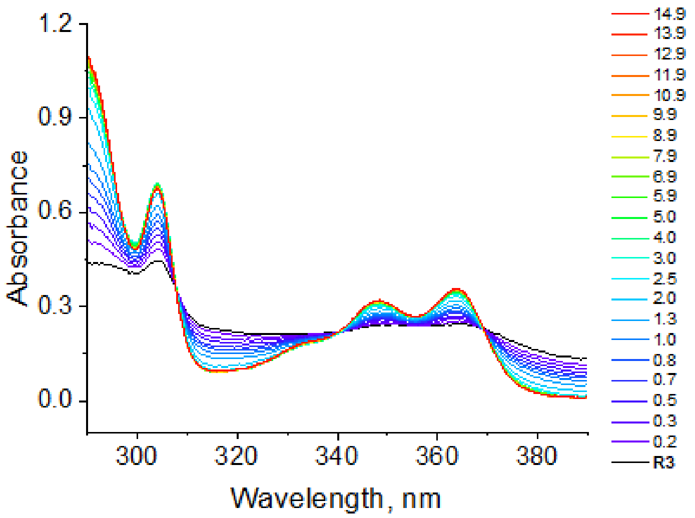

Figure S14. Stack of UV-vis spectra obtained during the titration of 5×10^{-5} M solution of receptor **R3** in $CHCl_3$ with 0.033 M solution of POPC (dissolved in $CHCl_3$).

Raw Data

Added volume of POPC, µl	Equivalents of POPC	Wavelength, nm				
		366	365	364	363	362
0.0	0.00	0.243404	0.243892	0.245269	0.244311	0.245320
0.5	0.17	0.250678	0.253175	0.253868	0.253337	0.250902
1.0	0.33	0.255781	0.260145	0.262248	0.261152	0.261937
1.5	0.50	0.263338	0.267958	0.270607	0.268948	0.267974
2.0	0.66	0.269924	0.276038	0.278958	0.279463	0.276502
2.5	0.83	0.273490	0.280472	0.286361	0.285195	0.283525
3.0	0.99	0.280523	0.288760	0.294624	0.294208	0.289608
4.0	1.32	0.288537	0.298096	0.304331	0.302441	0.299987
6.0	1.98	0.298498	0.314316	0.319008	0.318582	0.314630
7.5	2.48	0.310447	0.323814	0.332135	0.329751	0.326717
9.0	2.97	0.316032	0.332253	0.339935	0.341229	0.331440
12.0	3.96	0.319928	0.337270	0.345421	0.342807	0.335207
15.0	4.95	0.322841	0.339913	0.347345	0.345254	0.336400
18.0	5.94	0.323957	0.340914	0.349745	0.348668	0.338166
21.0	6.93	0.328900	0.344736	0.352856	0.350688	0.341508
24.0	7.92	0.326531	0.345010	0.353414	0.349032	0.339960
27.0	8.91	0.329736	0.346131	0.352276	0.349998	0.341985
30.0	9.90	0.325444	0.343350	0.353463	0.350462	0.342469
33.0	10.89	0.330016	0.347402	0.354741	0.352937	0.343845
36.0	11.88	0.329492	0.346301	0.355738	0.351049	0.343471
39.0	12.87	0.329429	0.348288	0.354772	0.351407	0.343644
42.0	13.86	0.331024	0.347213	0.354766	0.353675	0.343408
45.0	14.85	0.332687	0.350238	0.356921	0.354592	0.34400

Exemplary UV-vis titration curve (absorbance at 364 nm)

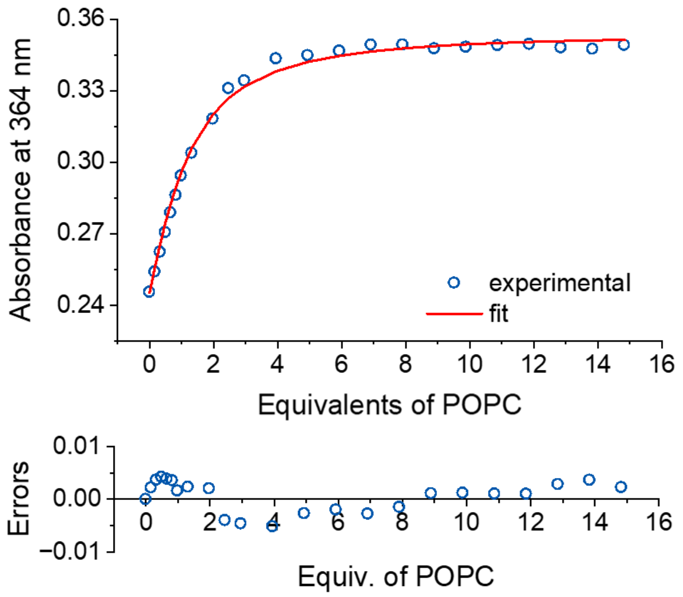

Figure S15. Fitting of 1:1 (receptor:lipid) binding model to the results of the titration of 5 × 10^{-5} M solution of receptor **R3** in $CHCl_3$ with 0.033 M solution of POPC (dissolved in $CHCl_3$).

a) Binding constant K derived from simultaneous fitting of 1:1 model to the five selected wavelengths using BindFit:

log K = 4.4676

b) Binding constant K derived from the experiment repeated according to the same methodology:

log K = 4.5248

c) Binding constant averaged from the two experiments:

log K = 4.50 ± 0.04

3.7 UV-vis titrations of **R4** with POPC in organic solvent

Stack of UV-vis spectra from the titration of R4 with POPC in $CHCl_3$

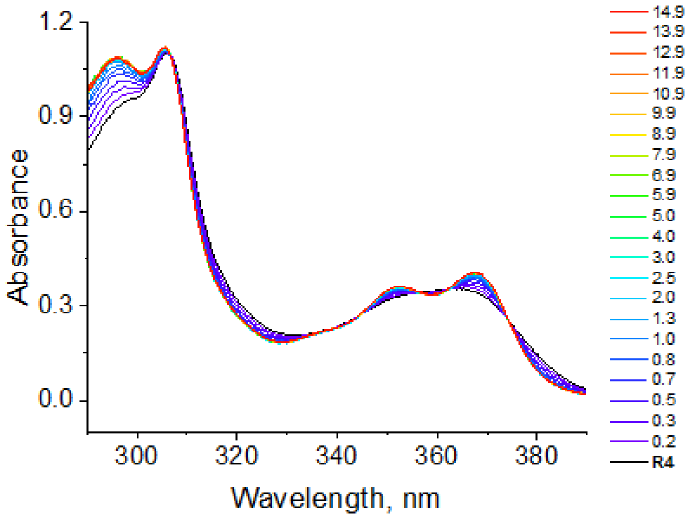

Figure S16. Stack of UV-vis spectra obtained during the titration of 5 × 10^{-5} M solution of receptor **R4** in $CHCl_3$ with 0.033 M solution of POPC (dissolved in $CHCl_3$).

Raw Data

Added volume of POPC, µl	Equivalents of POPC	Wavelength, nm				
		371	370	369	368	367
0.0	0.00	0.310305	0.323298	0.335179	0.341519	0.344558
0.5	0.17	0.317663	0.332271	0.346217	0.354012	0.356294
1.0	0.33	0.327211	0.342093	0.356104	0.361835	0.368311
1.5	0.50	0.333850	0.351648	0.367430	0.374908	0.375647
2.0	0.66	0.340123	0.360187	0.375681	0.380402	0.381243
2.5	0.83	0.345014	0.364663	0.380491	0.387439	0.387985
3.0	0.99	0.350235	0.369794	0.386971	0.390256	0.392465
4.0	1.32	0.355159	0.376286	0.392468	0.396348	0.396342
6.0	1.98	0.356496	0.379512	0.394935	0.400021	0.398474
7.5	2.48	0.356535	0.378638	0.397710	0.400212	0.400238
9.0	2.97	0.359494	0.380731	0.397402	0.402283	0.401268
12.0	3.96	0.360097	0.380629	0.398139	0.402898	0.402719
15.0	4.95	0.357898	0.380932	0.399454	0.403281	0.402272
18.0	5.94	0.360250	0.382189	0.398771	0.405395	0.402854
21.0	6.93	0.361172	0.381585	0.398129	0.402538	0.402626
24.0	7.92	0.358240	0.380570	0.399656	0.403252	0.402607
27.0	8.91	0.360644	0.381519	0.397593	0.401836	0.402843
30.0	9.90	0.359161	0.381312	0.398112	0.402087	0.402256
33.0	10.89	0.360360	0.382230	0.399743	0.404020	0.402794
36.0	11.88	0.359894	0.380952	0.396935	0.403325	0.402674
39.0	12.87	0.361016	0.381020	0.399891	0.403361	0.402473
42.0	13.86	0.363009	0.383614	0.400367	0.403632	0.405461
45.0	14.85	0.359955	0.382230	0.398369	0.404747	0.403730

Exemplary UV-vis titration curve (absorbance at 371 nm)

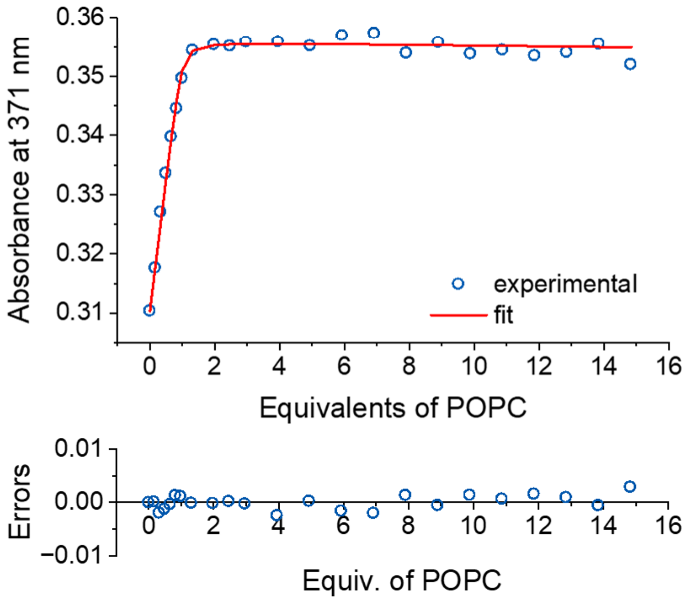

Figure S17. Fitting of 1:1 (receptor:lipid) model to the results of the titration of 5 × 10^{-5} M solution of receptor **R4** in $CHCl_3$ with 0.033 M solution of POPC (dissolved in $CHCl_3$).

a) Binding constant K derived from simultaneous fitting of 1:1 model to the five selected wavelengths using BindFit:

log K = 5.7860

b) Binding constant K derived from the experiment repeated according to the same methodology:

log K = 5.7491

c) Binding constant averaged from the two experiments:

log K = 5.77 ± 0.03

3.8 UV-vis titrations of **R1** with POPE in organic solvent

Stack of UV-vis spectra from the titration of R1 with POPE in $CHCl_3$

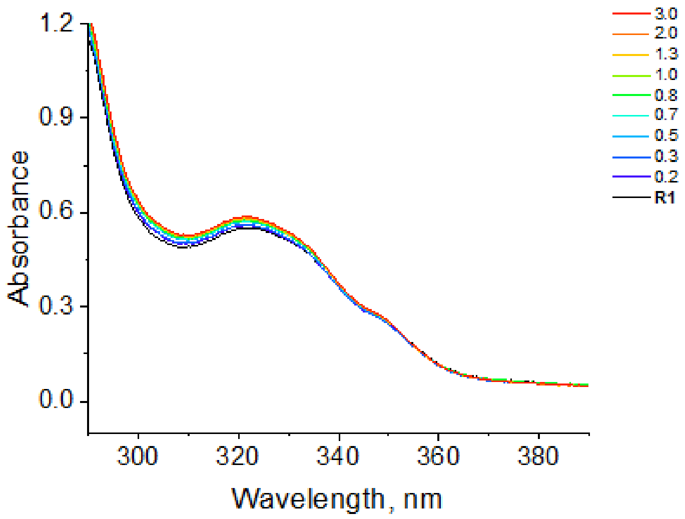

Figure S18. Stack of UV-vis spectra obtained during the titration of 5×10^{-5} M solution of receptor **R1** in $CHCl_3$ with 0.033 M solution of POPE (dissolved in $CHCl_3$).

Raw Data

Added volume of POPE, µl	Equivalents of POPE	Wavelength, nm				
		332	331	330	329	328
0.0	0.00	0.491291	0.501625	0.510845	0.518595	0.524944
0.5	0.17	0.493411	0.505503	0.514654	0.519835	0.529112
1.0	0.33	0.497245	0.507286	0.516032	0.523958	0.530722
1.5	0.50	0.504244	0.516410	0.526323	0.533858	0.539639
2.0	0.66	0.506243	0.519857	0.525703	0.535419	0.542930
2.5	0.83	0.509451	0.519783	0.529627	0.538895	0.546202
3.0	0.99	0.513003	0.523765	0.531667	0.540894	0.549636
4.0	1.32	0.512864	0.523259	0.530200	0.540168	0.551212
6.0	1.98	0.513129	0.526341	0.532941	0.541519	0.552558
9.0	2.97	0.517071	0.529753	0.537614	0.544348	0.554562

3.9 UV-vis titrations of R2 with POPE in organic solvent

Stack of UV-vis spectra from the titration of R2 with POPE in $CHCl_3$

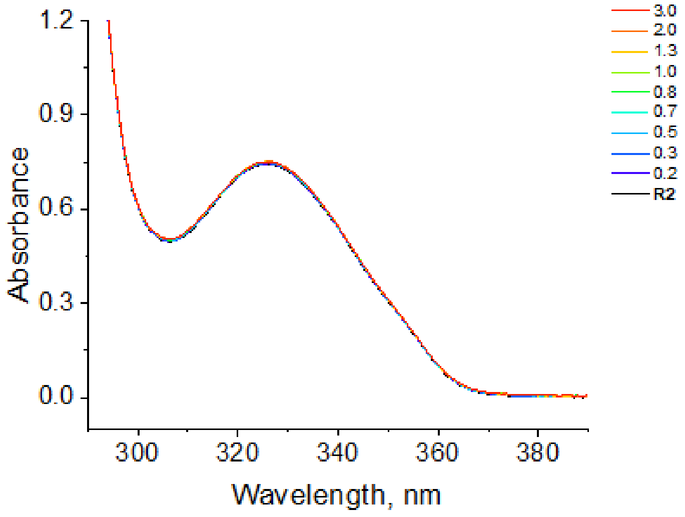

Figure S19. Stack of UV-vis spectra obtained during the titration of 5 × 10^{-5} M solution of receptor **R2** in $CHCl_3$ with 0.033 M solution of POPE (dissolved in $CHCl_3$).

Raw Data

Added volume of POPE, μl	Equivalents of POPE	Wavelength, nm				
		336	335	334	333	332
0.0	0.00	0.623330	0.644071	0.661998	0.679572	0.693505
0.5	0.17	0.625700	0.647799	0.665339	0.682564	0.695967
1.0	0.33	0.626512	0.648553	0.665036	0.684415	0.695917
1.5	0.50	0.627714	0.651332	0.667556	0.683753	0.698408
2.0	0.66	0.632651	0.651626	0.670385	0.687471	0.701452
2.5	0.83	0.632734	0.649719	0.670964	0.687094	0.702091
3.0	0.99	0.631627	0.651729	0.671991	0.689619	0.702449
4.0	1.32	0.631641	0.651933	0.670115	0.687204	0.701507
6.0	1.98	0.631365	0.655292	0.669734	0.686086	0.703171
9.0	2.97	0.631661	0.651841	0.671751	0.685416	0.702397

3.10 UV-vis titrations of **R3** with POPE in organic solvent

Stack of UV-vis spectra from the titration of R3 with POPE in $CHCl_3$

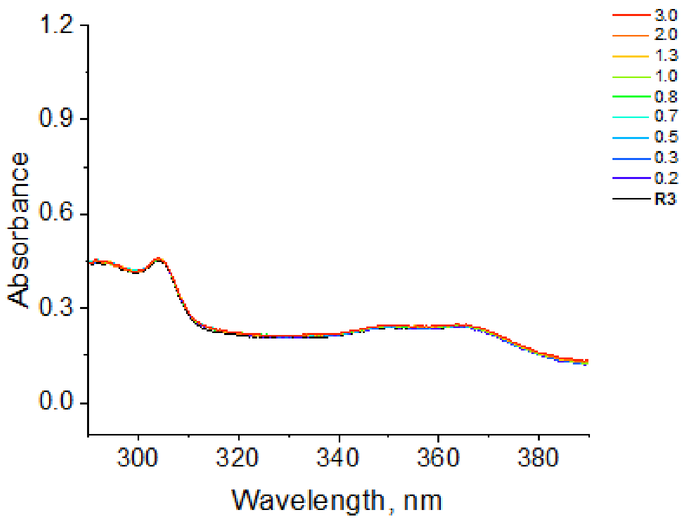

Figure S20. Stack of UV-vis spectra obtained during the titration of 5 × 10^{-5} M solution of receptor **R3** in $CHCl_3$ with 0.033 M solution of POPE (dissolved in $CHCl_3$).

Raw Data

Added volume of POPE, µl	Equivalents of POPE	Wavelength, nm				
		366	365	364	363	362
0.0	0.00	0.237837	0.241417	0.241075	0.240426	0.237614
0.5	0.17	0.238956	0.242497	0.239847	0.240477	0.238866
1.0	0.33	0.240089	0.243621	0.243586	0.243917	0.241512
1.5	0.50	0.241237	0.244159	0.245100	0.244860	0.242096
2.0	0.66	0.242630	0.248083	0.247330	0.247177	0.245723
2.5	0.83	0.243242	0.247497	0.247483	0.246668	0.243342
3.0	0.99	0.243376	0.245179	0.245617	0.244630	0.241809
4.0	1.32	0.246150	0.248928	0.248410	0.248740	0.247348
6.0	1.98	0.246730	0.248678	0.249162	0.247846	0.244911
9.0	2.97	0.246470	0.247485	0.248216	0.247548	0.246269

3.11 UV-vis titrations of **R4** with POPE in organic solvent

Stack of UV-vis spectra from the titration of R4 with POPE in $CHCl_3$

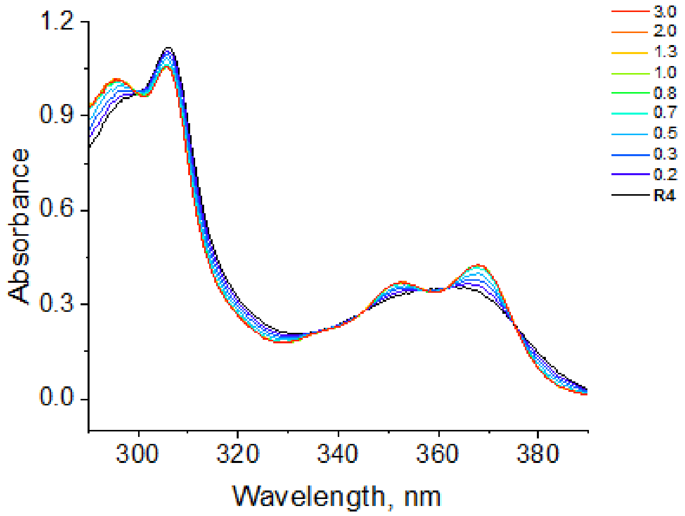

Figure S21. Stack of UV-vis spectra obtained during the titration of 5 × 10^{-5} M solution of receptor **R4** in $CHCl_3$ with 0.033 M solution of POPE (dissolved in $CHCl_3$).

Raw Data

Added volume of POPE, µl	Equivalents of POPE	Wavelength, nm				
		371	370	369	368	367
0.0	0.00	0.307492	0.320659	0.332954	0.340622	0.34526
0.5	0.17	0.325835	0.339134	0.353801	0.360212	0.361373
1.0	0.33	0.341985	0.358107	0.374610	0.379592	0.380284
1.5	0.50	0.356808	0.377360	0.392522	0.399211	0.395015
2.0	0.66	0.375089	0.396038	0.413313	0.416372	0.415630
2.5	0.83	0.383107	0.406065	0.422900	0.425579	0.421163
3.0	0.99	0.383643	0.405983	0.422693	0.424561	0.420474
4.0	1.32	0.382351	0.405610	0.424138	0.427666	0.421633
6.0	1.98	0.384069	0.405760	0.422534	0.425538	0.422930
9.0	2.97	0.383319	0.403614	0.422806	0.426212	0.421551

Exemplary UV-vis titration curve (absorbance at 371 nm)

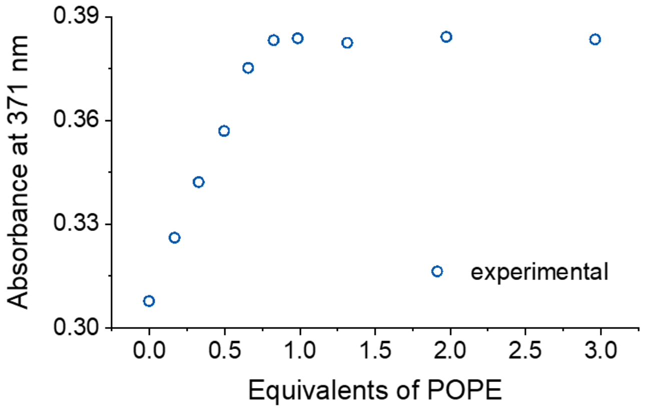

Figure S22. Fitting of 1:1 (receptor:lipid) model to the results of the titration of 5 × 10^{-5} M solution of receptor **R4** in $CHCl_3$ with 0.033 M solution of POPE (dissolved in $CHCl_3$). The curve is too sharp to be reliably reproduced by fitting software (**log K>7**).

3.12 Fluorescence titrations in POPC and POPE:POPC liposomes

To determine the interaction between the receptors **R1**–**R4** and PC or PE lipids that are part of a bilayer (membrane), we performed fluorescence titrations whereby aliquots of the hosts in DMSO were added to an aqueous suspension of 100 nm large unilamellar vesicles (LUVs) containing NBD-labelled lipids, NBD-PC and NBD-PE, based on a published protocol.[6] Due to the inability of pure POPE to form stable liposomes, a mixture of 9:1 POPE:POPC was used. The titrations were thus performed with either 100 nm POPC LUVs containing 1 mol% NBD-PC, or 100 nm 9:1 POPE:POPC LUVs containing 1 mol% NBD-PE.

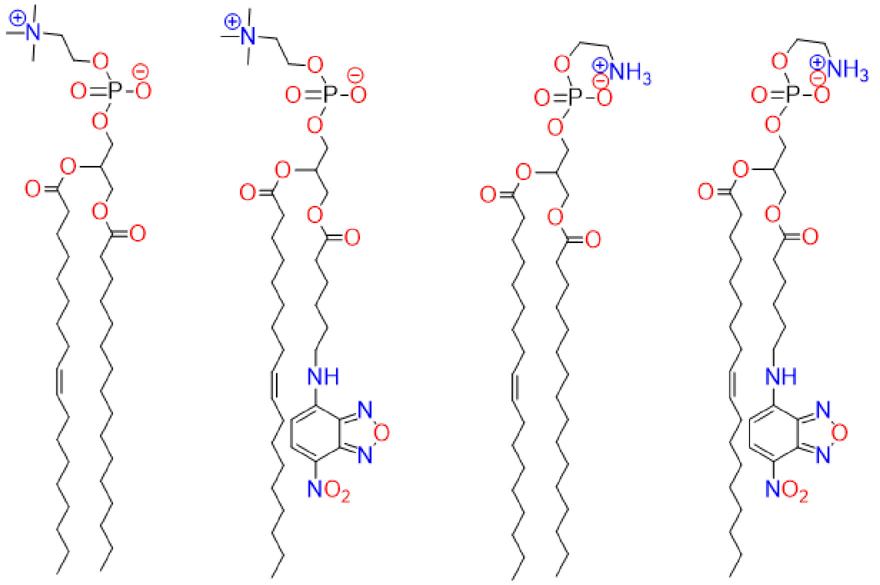

To prepare the LUVs, the unlabelled lipids (POPC or POPE:POPC 9:1) were weighed out in a 10 mL round bottom flask, and 1 mol% NBD-labelled lipid was added from a 1 mg/mL stock in chloroform (mol% with respect to total unlabelled lipid). The lipids were subsequently dissolved in chloroform to generate a homogenous mixture. The chloroform was removed on a rotary evaporator and the residue was dried under high vacuum overnight. The lipid film was hydrated with HEPES buffer (0.8 mL, 10 mM HEPES, 150 mM $NaNO_3$, pH 7.4), kept for 30 s in an ultrasonic bath and vortexed for 30 min. The resulting suspension was subjected to 11 freeze-thaw cycles, alternating between submersion in liquid nitrogen followed by thawing in mildly warm water. The lipid suspension was allowed to rest at room temperature for 30 minutes before extruding 35 times through a 100 nm polycarbonate membrane (*Nucleopore*) using the Avanti mini extruder set (*Avanti Polar Lipids, Inc.*).

The collected vesicles were diluted with the HEPES buffer to obtain liposomes stock solution at lipids concentration ≈ 0.32 mM. For each titration, the lipid stock solution was diluted with HEPES buffer to obtain 2.5 mL of a 12.5 µM lipid solution in a fluorescence cuvette. A cuvette stir bar was added to achieve adequate mixing upon each addition. The emission spectrum was measured using an Agilent Cary Eclipse fluorescence spectrophotometer equipped with stirring function and Peltier temperature controller (excitation wavelength = 470 nm).

[6] S. R. Herschede, H. Gneid, T. Dent, E. B. Jaeger, L. B. Lawson and N. Busschaert, *Org. Biomol. Chem.*, **2021**, *19*, 3838–3843.

Aliquots of the hosts in DMSO (2 mM stock) were added and the emission spectrum was obtained upon each addition. The final addition corresponded to 12.7 µM hosts and a total volume of 16 µL DMSO. A control experiment where the same volume of neat DMSO was added was also performed.

Fluorescence intensity was normalized by dividing the fluorescence intensity at every wavelength by the fluorescence intensity at 530 nm prior to the addition of compound (maximum fluorescence) from control experiment.

3.13 Determination of Stern-Volmer constants

Where there was a significant change in fluorescence intensity, a Stern-Volmer analysis was performed. F_0/F values, whereby F_0 is the intensity at 530 nm before the addition of host and F is the intensity at 530 nm upon each addition, were plotted against the concentration of receptors and fitted with the following equation using OriginPro2022:

$$\frac{F_0}{F} = K_{SV} \cdot [R] + 1$$

where K_{SV} is the Stern-Volmer constant and $[R]$ is the concentration of a quencher – receptor. Fits were fixed at y(0) = 1.

It was found that the data was linear for the interaction of lipids with compounds **R1**–**R4**. The results are shown in Figures S23–S30. The titrations were repeated a minimum of 3 times (independent repeats), and the Stern-Volmer constant was calculated for each repeat and subsequently averaged.

3.14 Fluorescence titration of POPC liposomes with **R1**–**R4**

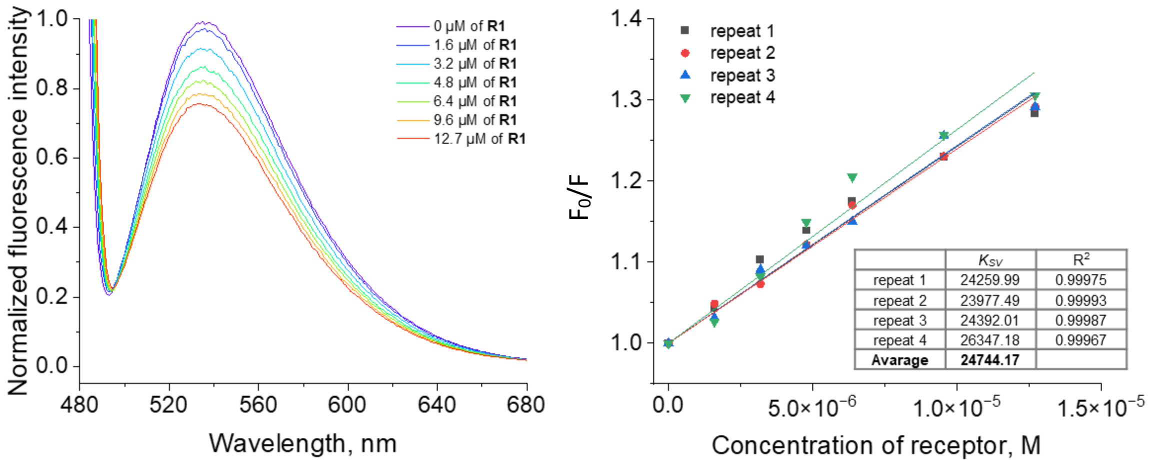

Figure S23. Fluorescence titration of liposome suspension (100 nm LUVs, POPC liposomes containing 1 mol% NBD-PC) with **R1** (2 mM in DMSO). Excitation wavelength = 470 nm, temperature = 25 °C. Exemplary normalized fluorescence spectra (left). Stern-Volmer plots for all individual repeats (right).

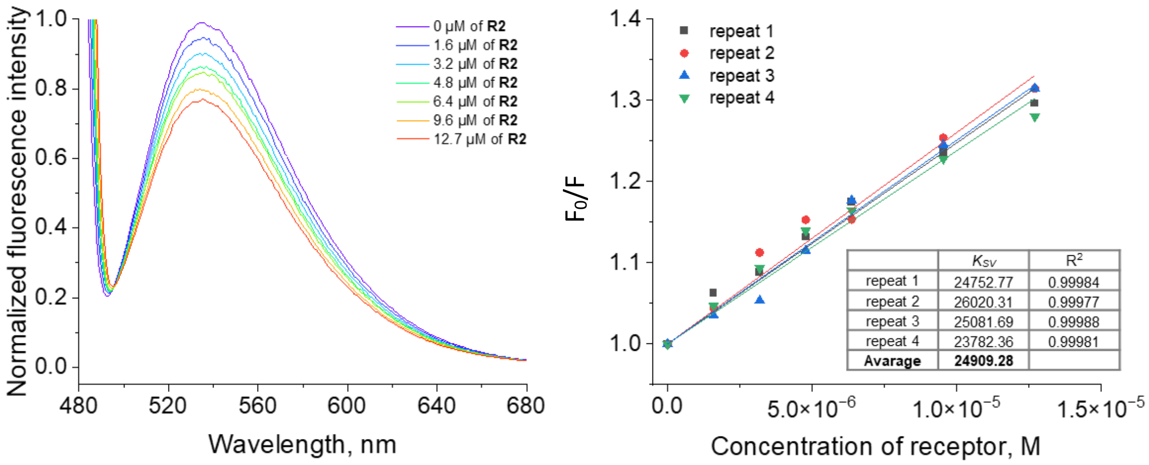

Figure S24. Fluorescence titration of liposome suspension (100 nm LUVs, POPC liposomes containing 1 mol% NBD-PC) with **R2** (2 mM in DMSO). Excitation wavelength = 470 nm, temperature = 25 °C. Exemplary normalized fluorescence spectra (left). Stern-Volmer plots for all individual repeats (right).

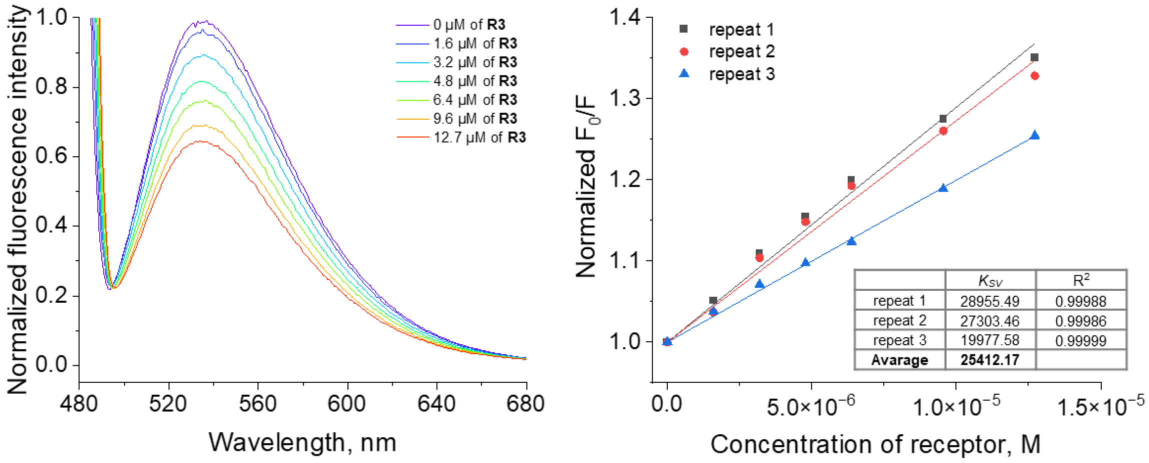

Figure S25. Fluorescence titration of liposome suspension (100 nm LUVs, POPC liposomes containing 1 mol% NBD-PC) with **R3** (2 mM in DMSO). Excitation wavelength = 470 nm, temperature = 25 °C. Exemplary normalized fluorescence spectra (left). Stern-Volmer plots for all individual repeats (right).

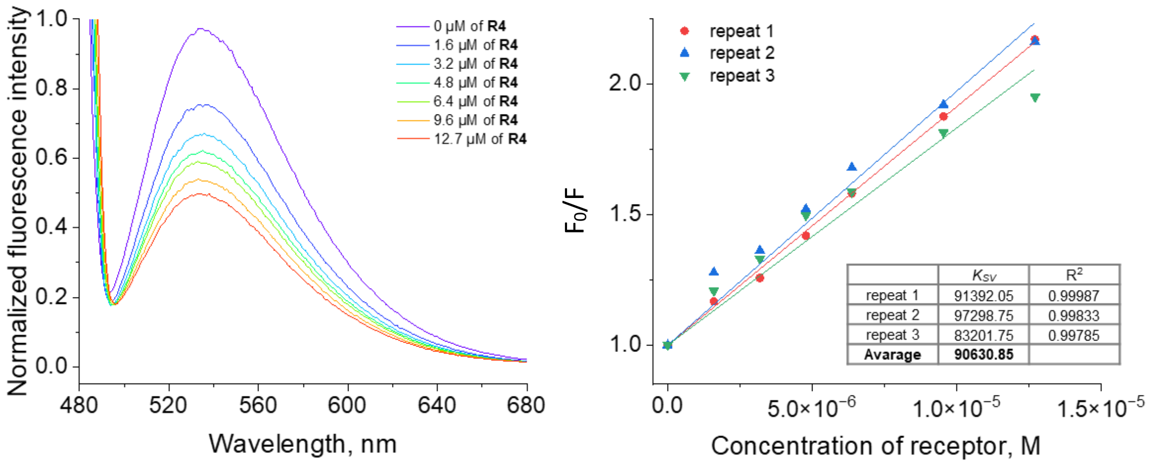

Figure S26. Fluorescence titration of liposome suspension (100 nm LUVs, POPC liposomes containing 1 mol% NBD-PC) with **R4** (2 mM in DMSO). Excitation wavelength = 470 nm, temperature = 25 °C. Exemplary normalized fluorescence spectra (left). Stern-Volmer plots for all individual repeats (right).

3.15 Fluorescence titration of POPE:POPC 9:1 liposomes with **R1**–**R4**

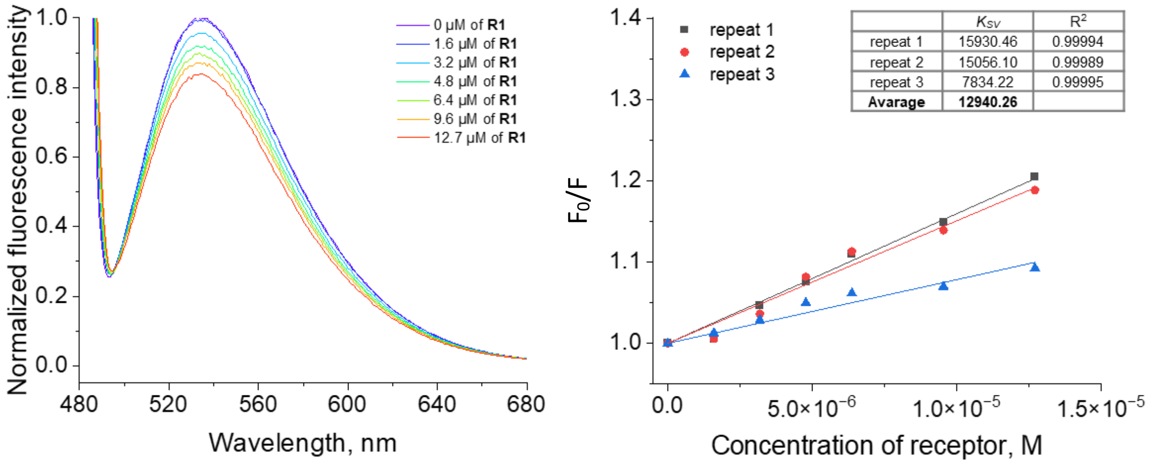

Figure S27. Fluorescence titration of liposome suspension (100 nm LUVs, POPE:POPC 9:1 liposomes containing 1 mol% NBD-PE) with **R1** (2 mM in DMSO). Excitation wavelength = 470 nm, temperature = 25 °C. Exemplary normalized fluorescence spectra (left). Stern-Volmer plots for all individual repeats (right).

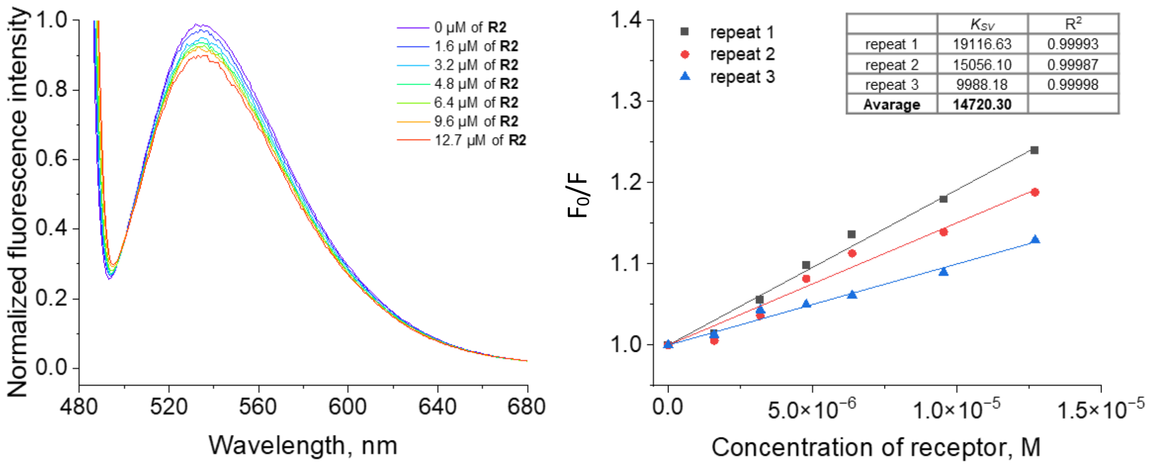

Figure S28. Fluorescence titration of liposome suspension (100 nm LUVs, POPE:POPC 9:1 liposomes containing 1 mol% NBD-PE) with **R2** (2 mM in DMSO). Excitation wavelength = 470 nm, temperature = 25 °C. Exemplary normalized fluorescence spectra (left). Stern-Volmer plots for all individual repeats (right).

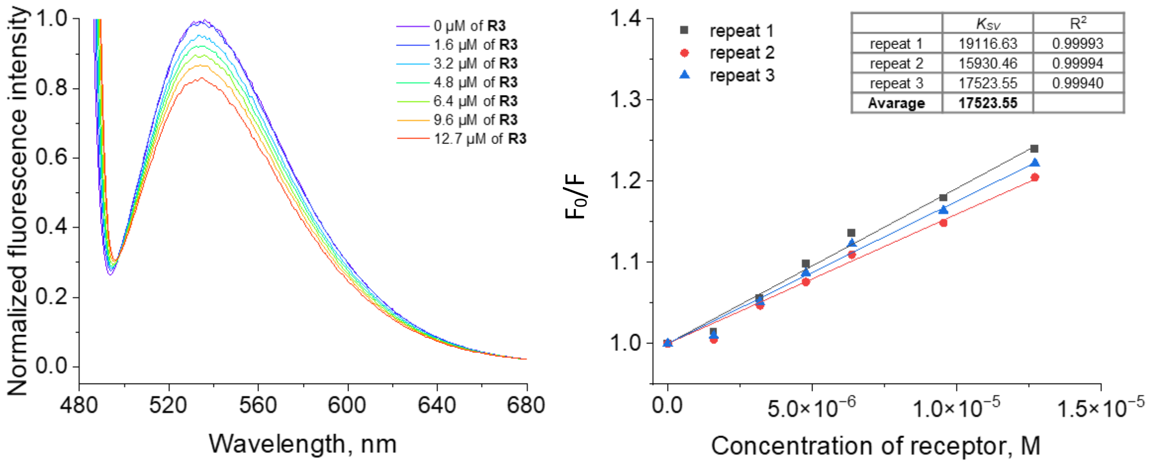

Figure S29. Fluorescence titration of liposome suspension (100 nm LUVs, POPE:POPC 9:1 liposomes containing 1 mol% NBD-PE) with **R3** (2 mM in DMSO). Excitation wavelength = 470 nm, temperature = 25 °C. Exemplary normalized fluorescence spectra (left). Stern-Volmer plots for all individual repeats (right).

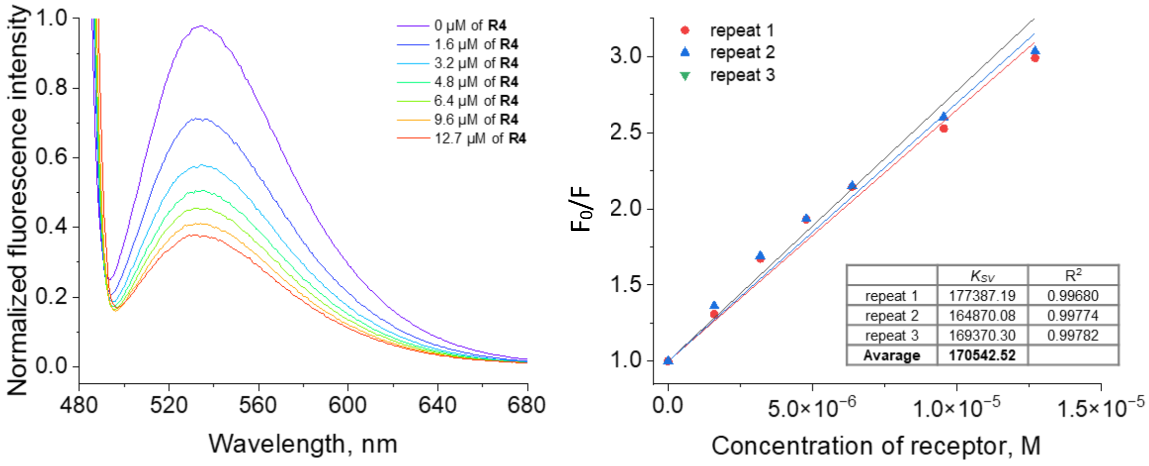

Figure S30. Fluorescence titration of liposome suspension (100 nm LUVs, POPE:POPC 9:1 liposomes containing 1 mol% NBD-PE) with **R4** (2 mM in DMSO). Excitation wavelength = 470 nm, temperature = 25 °C. Exemplary normalized fluorescence spectra (left). Stern-Volmer plots for all individual repeats (right).

4 Anion transport studies

4.1 General procedure for measuring anion transport

In a 10 mL round bottom flask the mixture of POPC and cholesterol (7:3 molar ratio) in chloroform was prepared. The organic solvent was evaporated on a rotary evaporator and the residue was dried under high vacuum overnight. The lipid film was hydrated with 0.500 mL of buffered aqueous solution containing lucigenin (0.8 mM lucigenin, 225 mM $NaNO_3$ (or 75 mM Na_2SO_4 for symport assays), 10 mM HEPES, pH 7.4), sonicated for 30 s and vortexed for 1 h resulting in vesicle formation. The multilamellar vesicles were broken down into unilamellar vesicles by 10 freeze-thawing cycles, diluted to 1 mL by the addition of buffer (225 mM $NaNO_3$ (or 75 mM Na_2SO_4 for symport assays), 10 mM HEPES, pH 7.4), and extruded 29 times through a polycarbonate membrane (200 nm pore size) to give homogeneous large unilamellar vesicles. Unencapsulated lucigenin was removed by passing the mixture through a column with Sephadex 50G (ca. 2 g, superfine) using buffer (225 mM $NaNO_3$ (or 75 mM Na_2SO_4 for symport assays), 10 mM HEPES, pH 7.4) as eluent. The collected vesicles were diluted with buffer (225 mM $NaNO_3$ (or 75 mM Na_2SO_4 for symport assays), 10 mM HEPES, pH 7.4) to reach a lipid concentration of 0.4 mM. 3 mL of the vesicle suspension was placed in a quartz cuvette with a small stirring bar, and receptor was added as a MeOH solution to achieve a concentration of 1 mol% related to total lipids (5 µL of 2.4 mM). The fluorescence was measured as a function of time (excitation: 455 nm, emission: 505 nm). Aqueous NaCl, KCl, RbCl or CsCl (75 µL, 1.0 M in 225 mM $NaNO_3$ $NaNO_3$ (or 75 mM Na_2SO_4 for symport assays), 10 mM HEPES, pH 7.4) was added 30 s after the start of fluorescence measurement to give an overall external Cl^- concentration of 25 mM. After 5 min, Triton X-100 was added to lyse the liposomes. For each run the initial plateau (before the addition of chloride) and the vertical drop (the first 0.5–2.0 s after chloride addition) due to quenching of residual external lucigenin were removed. Next, the data were normalized as:

$$Normalized\ fluorescence = \frac{\frac{F_0}{F} - 1}{\frac{F_0}{F_{max}} - 1}$$

4.2 Chloride transport by **R1**–**R4**

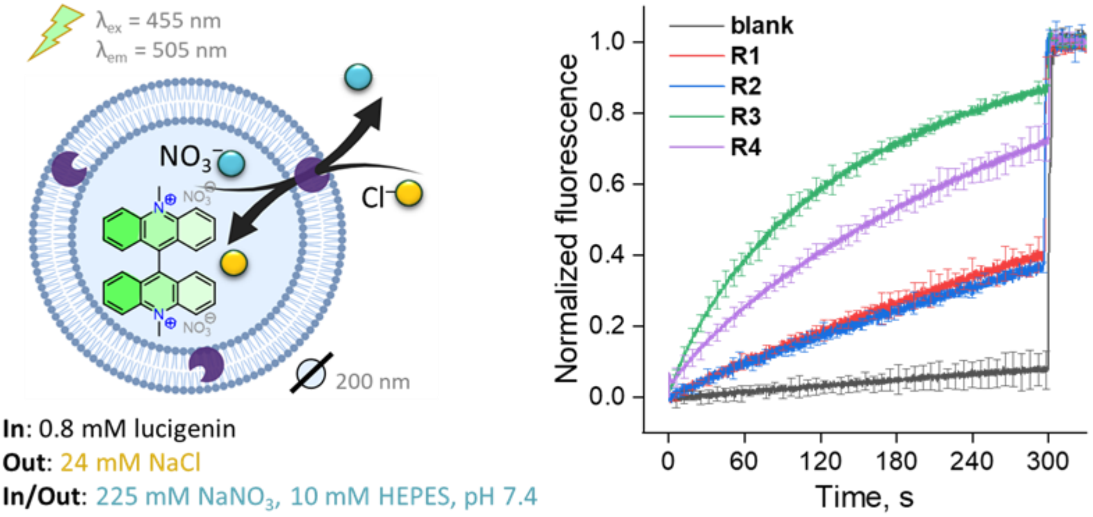

Figure S31. Schematic representation of lucigenin assay in POPC:cholesterol liposomes (left), normalized transport curves for post-inserted **R1**–**R4** at 1 mol% (right).

4.3 Quantification of the transport rate

Data fitting to obtain transport rates was performed using OriginPro 2022. The first 30 seconds of averaged and normalized data were removed, leaving addition of NaCl at t = 0 s and next 300 s of the traces were fitted with a single exponential decay function:

$$F = y0 - a \cdot exp(-k \cdot x)$$

where $y0$, a and k were treated as fitting parameters.

4.4 Determination of chloride transport rate

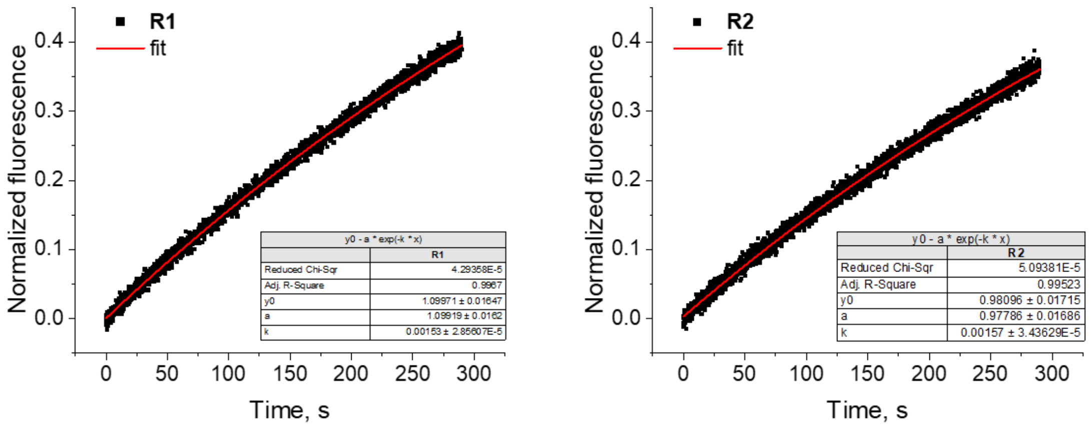

Figure S32. Normalized fluorescence and single exponential fit for the transport of Cl⁻ with **R1** (left) and **R2** (right) at 1 mol%.

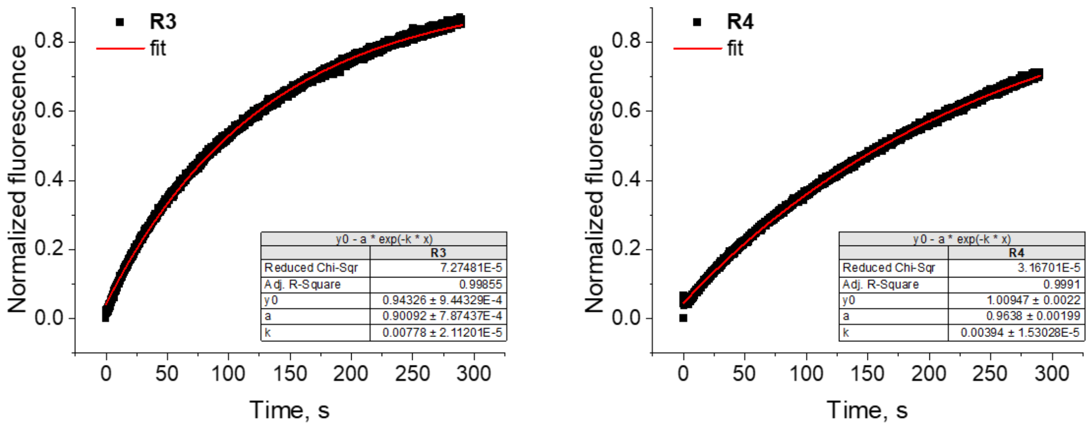

Figure S33. Normalized fluorescence and single exponential fit for the transport of Cl⁻ with **R3** (left) and **R4** (right) at 1 mol%.

The rate constants (k) give approximate half-times according to the equation: $t_{1/2} = ln(2)/k$.

	**R1**	**R2**	**R3**	**R4**
$t_{1/2}$(lucigenin)	453	441	89	176

4.5 Symport assay

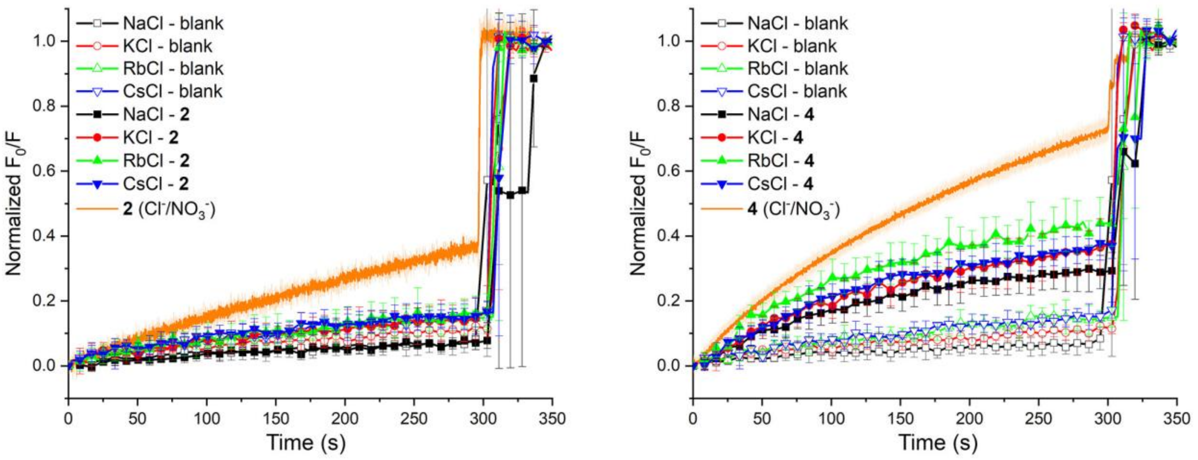

Figure S34. Symport assay. 200 nm 7:3 POPC:cholesterol LUVs were prepared encapsulating 0.8 mM lucigenin, 75 mM Na_2SO_4 for symport assays, 10 mM HEPES, pH 7.4, and suspended in 75 mM Na_2SO_4, 10 mM HEPES, pH 7.4. Transport was started by the addition of 25 mM NaCl, KCl, RbCl or CsCl, and MeOH (blank) or 1 mol% **R2** or **R4**. (left) results for **R2**, and (right) results for **R4**. The orange solid line or the results for the Cl^-/NO_3^- antiport assay shown in Figure S31 as comparison.

4.6 HPTS assay

In a 10 mL round bottom flask the mixture of POPC and cholesterol (7:3 molar ratio) in chloroform was prepared. The organic solvent was evaporated on a rotary evaporator and the residue was dried under high vacuum overnight. The lipid film was hydrated with 0.500 mL of buffered aqueous solution containing HPTS (1 mM HPTS, 100 mM NaCl, 10 mM HEPES, pH 7.0), sonicated for 30 s and vortexed for 1 h resulting in vesicle formation. The multilamellar vesicles were broken down into unilamellar vesicles by 10 freeze-thawing cycles, diluted to 1 mL by the addition of buffer (100 mM NaCl, 10 mM HEPES, pH 7.0), and extruded 29 times through a polycarbonate membrane (200 nm pore size) to give homogeneous large unilamellar vesicles. Unencapsulated HPTS was removed by passing the mixture through a column with Sephadex 50G (ca. 2 g, superfine) using buffer (100 mM NaCl, 10 mM HEPES, pH 7.0) as eluent. The collected vesicles were diluted with buffer (100 mM NaCl, 10 mM HEPES, pH 7.0) to reach a lipid concentration of 0.1 mM. Vesicle suspension (0.1 mM, 3 mL) was placed in a quartz cuvette with a small stirring bar, and receptor was added as a MeOH solution (5 µL) to achieve a concentration of 1 mol% related to total lipids. The fluorescence emission of the intravesicular HPTS was recorded using a fluorometer as a function of time. An NaOH pulse (25 µL, 0.5 M) was added to basify the external solution to pH 8.0 to initiate the start of the experiment. After t = 210 s a solution of Triton X-100 (11 w%) in DMSO:H_2O (1:7, v/v) (50 µL) was added to lyse the vesicles to fully dissipate the pH gradient and a final fluorescence reading was t = 300 s. DMSO control experiments were performed in the absence of the transporter, and experiments were independently repeated in triplicate.

HPTS was used as a ratiometric probe to measure the H^+/Cl^- symport (or equivalent OH^-/Cl^- antiport) through the intravesicular pH change during the experiment. The acidic and basic forms of HPTS were excited at λ_{ex} = 403 nm and λ_{ex} = 460 nm respectively, and the fluorescence emission of both forms was collected at λ_{em} = 510 nm. The fluorescence intensity ratio (R) of the basic form and the acidic form of HPTS was calculated, which allowed the calculation of the fractional fluorescence intensity (I_f) using the equation:

$$I_f = \frac{R_t - R_0}{R_d - R_0}$$

Where R_t is the ratiometric fluorescence value at a given time t, R_0 is the ratiometric fluorescence value at t = 0 s and R_d is the fluorescence ratiometric value recorded at t = 300 s following vesicular lysis.

4.7 Equilibration of pH gradient by **R1**–**R4**

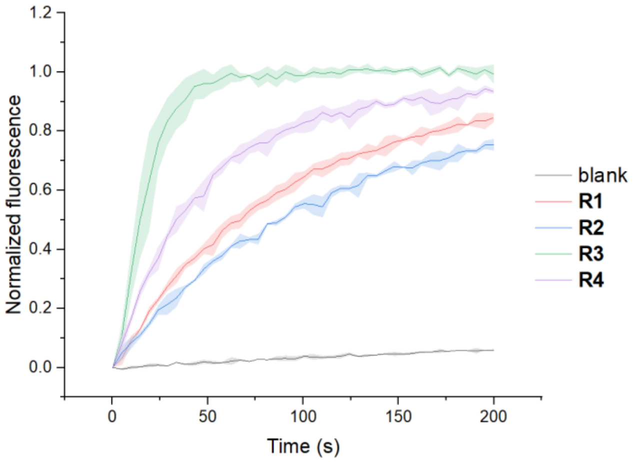

Figure S35. Normalized transport curves for post-inserted **R1**–**R4** at 1 mol% (HPTS assay).

4.8 Determination of transport rate

Data fitting to obtain transport rates was performed using OriginPro 2022 by fitting the traces with a single exponential decay function:

$$F = y0 - a \cdot exp(-k \cdot x)$$

where $y0$, a and k were treated as fitting parameters.

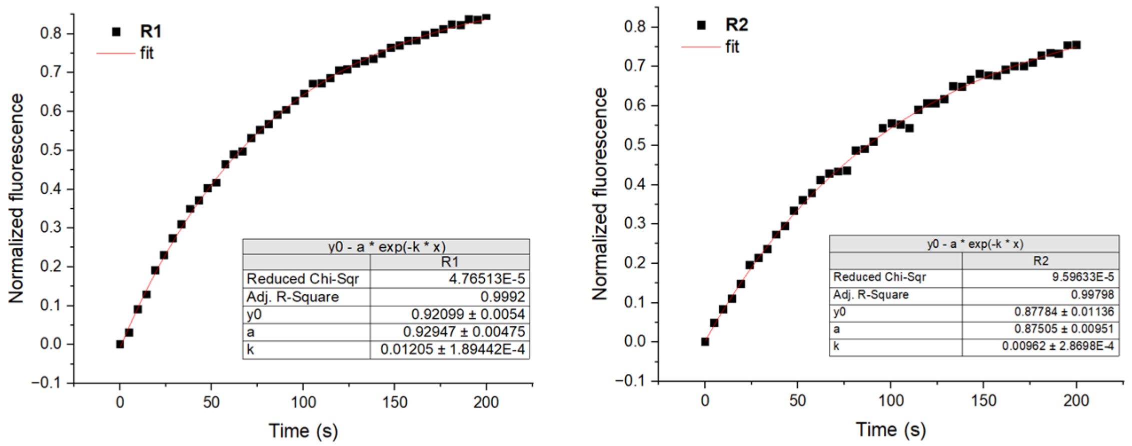

Figure S36. Normalized fluorescence and single exponential fit for the equilibration of pH gradient with **R1** (left) and **R2** (right) at 1 mol%.

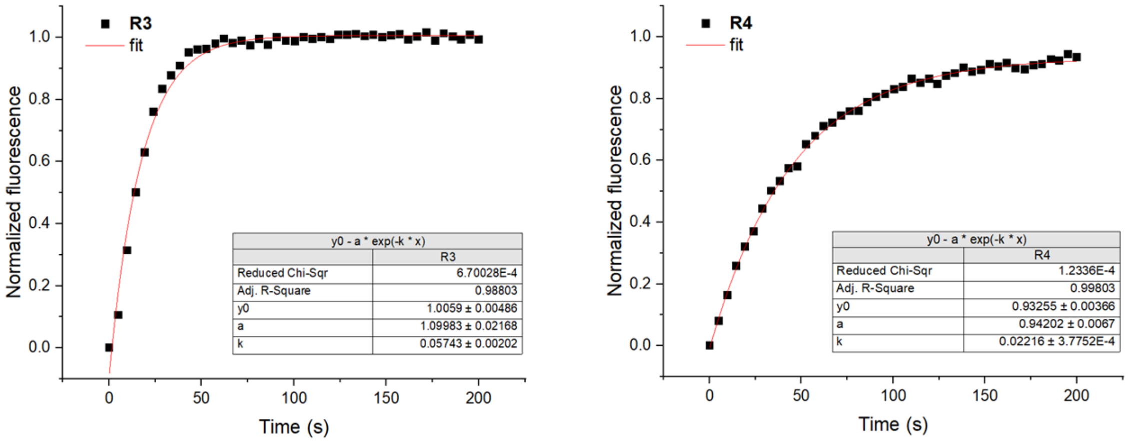

Figure S37. Normalized fluorescence and single exponential fit for the equilibration of pH gradient with **R1** (left) and **R2** (right) at 1 mol%.

The rate constants (k) give approximate half-times according to the equation: $t_{1/2} = ln(2)/k$.

	R1	R2	R3	R4
$t_{1/2}$ (HPTS)	58	72	12	31

5 Biological studies

5.1 Lipid composition of bacteria used

While detailed lipidomics data for the specific ATCC strains used in our study (see below) are not available, approximate membrane compositions reported in the literature are summarized in the **Table S1** below.

Table S1. Membrane phospholipid constitution of various bacteria. Percentages refer to major phospholipid headgroups in total membrane lipids.

Bacteria	Phosphatidylethanolamine (PE)	Phosphatidylglycerol (PG)	Cardiolipin (CL)	Lysyl phosphatidyl-glycerol (Lys-PG)
Bacillus cereus [7]	~50–60%	~30–40%		
Bacillus subtilis [8]	~20%	~70%	~5%	~2%
Staphylococcus aureus [9]	0%	~55%	~5%	~40%
Enterococcus faecalis [10]	0%	~54%	~1%	~1%
Escherichia coli [11]	~75% *	~20%	~5%	

* - PE is predominantly located in the inner membrane with 75%/25% (cytoplasmic/periplasmic leaflet) distribution.

5.2 MIC determination

The minimum inhibitory concentration (MIC) against the Gram-positive bacteria *Bacillus cereus*, *Bacillus subtilis*, *Staphylococcus aureus* and *Enterococcus faecalis*, and the Gram-negative bacterium *Escherichia coli*, was determined using the broth microdilution method recommended by the Clinical and Laboratory Standards Institute.[12] All bacteria were obtained from the American Type Culture Collection (*B. cereus* – ATCC 117781, *B. subtilis* – ATCC 6051, *S. aureus* – ATCC 25923, *E. faecalis* – ATCC 29212, and *E. coli* – ATCC 25922) and stored in glycerol stocks at -80 °C.

For each experiment, a small amount of the glycerol stock was streaked onto a Müller-Hinton agar plate (Sigma-Aldrich #70191) and the agar plate was incubated for 24 hours at 35 °C. The obtained colonies were aseptically transferred into sterile cation-adjusted Müller-Hinton broth (Sigma-Aldrich #90922) and vortex briefly. Colonies were added until the inoculum solution achieved an OD_{600} value corresponding to 1×10^8 CFU/mL. OD_{600} values were determined using a Biowave CO8000 Cell Density meter and 17×100 mm polystyrene culture tubes (VWR #60818-703). The inoculum was subsequently

---

[7] Lang Dennis, R.; Lundgren, D. G. Lipid Composition of Bacillus cereus During Growth and Sporulation. *J. Bacteriol.* **1970**, *101* (2), 483.

[8] Kira L. F. Hilton, K.L.F.; Manwani, C.; Boles, J.E.; White, L.J.; Ozturk, S.; Garrett, M.D.; Hiscock, J.R. The phospholipid membrane compositions of bacterial cells, cancer cell lines and biological samples from cancer patients. *Chem. Sci*. **2021**, *12*, 13273.

[9] Dennison, S.R.; Morton, L.H.G.; Harris, F.; Phoenix, D.A. Low pH Enhances the Action of Maximin H5 against Staphylococcus aureus and Helps Mediate Lysylated Phosphatidylglycerol-Induced Resistance. *Biochemistry* **2016**, *55*, 3735.

[10] Woodall, B.M.; Harp, J.R.; Brewer, W.T.; Tague, E.D.; Campagna, S.R.; Fozo, E.M. Enterococcus faecalis Readily Adapts Membrane Phospholipid Composition to Environmental and Genetic Perturbation. *Front Microbiol*. **2021**, 12:616045.

[11] Rowlett, V.W.; Mallampalli, V.K.P.S.; Karlstaedt, A.; Dowhan, W.; Taegtmeyer, H.; Margolin, W.; Vitrac, H. Impact of Membrane Phospholipid Alterations in Escherichia coli on Cellular Function and Bacterial Stress Adaptation. *J Bacteriol*. **2017**, 199 (13):e00849-16.

[12] CLSI, Methods for dilution antimicrobial susceptibility tests for bacteria that grow aerobically; approved standard. 11th ed. CLSI standard M07 ed.; Clinical and Laboratory Standards Institute: Wayne, PA, **2018**; Vol. 32.

diluted to 5×10^5 CFU/mL in sterile cation-adjusted Müller-Hinton broth. 192 µL of this inoculum was transferred to the wells of a sterile flat-bottom polystyrene non-tissue culture treated 96-well plate (Falcon #351172) and 8 µl of a DMSO stock solution of compounds **R1**–**R4** was added to each well (final (DMSO concentration 4%) and the 96-well plate was covered with a Breathe-Easy sealing membrane (Sigma-Aldrich #Z380059). The DMSO concentration was kept constant at 4% (v/v) in all experiments.

The 96-well plates were subsequently incubated, and MIC values were determinated visually after 24 h of incubation at 35 °C. The experiment was performed in biological triplicate, including 2 technical repeats each. The MICs value was defined as the minimum concentration of compound that resulted in complete inhibition of bacterial growth over the full 24 hours. Clindamycin was used for quality control reasons.

5.3 Sytox Green assay

To determine if the compounds **R3**–**R4** cause the formation of large pores, or lead to other large defects or disruption of the membrane, we performed a fluorescence assay with Sytox Green.[13] For each experiment, a small amount of the *B. subtilis* glycerol stock was streaked onto a Müller-Hinton agar plate and the agar plate was incubated for 18–24 hours at 35 °C. The obtained colonies were aseptically transferred into sterile cation-adjusted Müller-Hinton broth and diluted to OD_{600} of 0.2. The bacteria were subsequently incubated at 35 °C until they reached mid-logarithmic phase. The cultures were then centrifuged at 3000 rpm for 5 mins and the pellets were washed twice with phosphate-buffered saline (PBS) and diluted in PBS to an OD_{600} of 0.2. Then, Sytox Green dissolved in DMSO was added to achieve a final Sytox Green concentration of 1 µM and 1% DMSO, and the bacteria were incubated at 35 °C for 15 minutes. Then, 180 µl of the bacteria suspension was transferred into the wells of a 96-well plate for fluorescence (sterile, black, flat-bottom, polystyrene microplate from Brand #7816668) and the fluorescence intensity was measured for 4 minutes. At this point, 20 µl of stock solutions of receptors **R3**–**R4** and control antibiotics (dissolved to 10× the desired concentration in PBS buffer with 1% DMSO) were added and the fluorescence was measured for 2 hours. All fluorescence measurements were preformed using a BioTek Cytation 5 Cell Imaging Multi-Mode Reader (35 °C, excitation at 485 nm and emission at 520 nm, time intervals of 23 seconds, orbital shaking (5 seconds at 548 rpm (2 mm) before each measurement). As a positive control, 2.5 µM nisin was used, which is known to form large pores that allow Sytox Green to enter the bacterial cells.[14] As a negative control, the ribosome-targeting antibiotic clindamycin was used (2 µg/mL), as well as gramicidin (1 µM) and a blank run (no antibiotic added). Neither compound **R3** nor **R4** leads to an increase in fluorescence, indicating that they do not form large pores (Figure S38).

[13] B. L. Roth, M. Poot, S. T. Yue and P. J. Millard, *Appl. Environ. Microbiol.*, **1997**, *63*, 2421–2431.
[14] K. M. Scherer, J.-H. Spille, H.-G. Sahl, F. Grein and U. Kubitscheck, *Biophys. J.*, **2015**, *108*, 1114–1124.

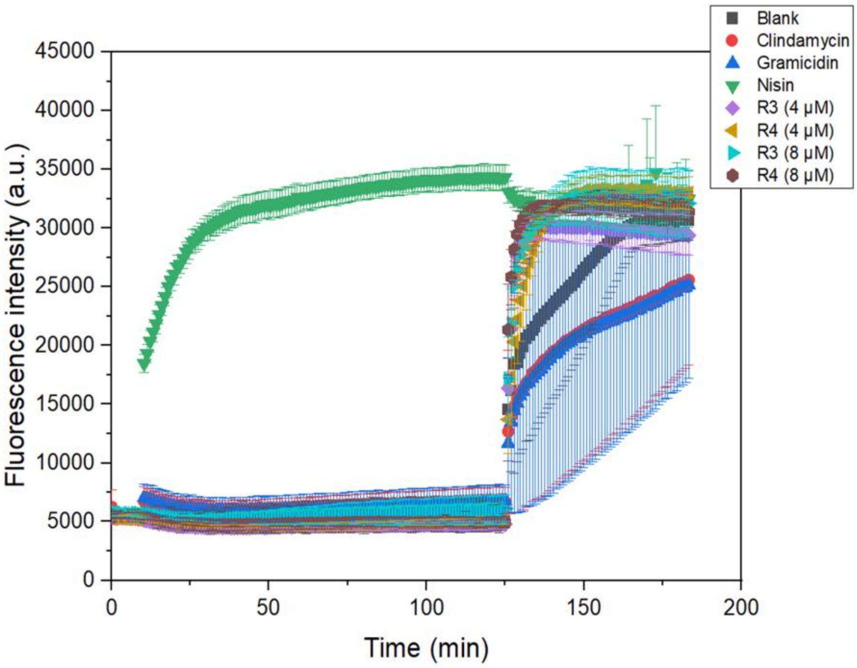

Figure S38. Fluorescence intensity of Sytox Green in *B. subtilis* in the presence of DMSO (1%, blank), clindamycin (2 µg/mL, negative control), gramicidin (1 µM, negative control), nisin (2.5 µM, positive control), 4 µM **R3**, 8 µM **R3**, 4 µM **R4**, or 8 µM **R4**. Results are the average of at least 2 technical x 2 biological repeats and error bars represent standard deviations.

5.4 Membrane depolarization assay

A membrane depolarization assay was performed using the dye 3,3′-dipropylthiadicarbocyanine iodide $DiSC_3(5)$, according to the method by te Winkel *et al.*[15] For each experiment, a small amount of the *B. subtilis* glycerol stock was streaked onto a Müller-Hinton agar plate (Sigma-Aldrich #70191) and the agar plate was incubated for 18–24 hours at 35 ^{o}C. The obtained colonies were aseptically transferred into sterile cation-adjusted Müller-Hinton broth (Sigma-Aldrich #90922) and diluted to OD_{600} of 0.2. The bacteria were subsequently incubated at 35 ^{o}C until they reached mid-logarithmic phase. The cultures were then centrifuged at 3000 rpm for 5 mins and the pellets were re-suspended and diluted in Müller-Hinton broth supplemented with 0.5 mg/mL BSA (bovine serum albumin) to an OD_{600} of 0.2.

Then, 178 µl of the diluted cells were transferred to a fluorescence 96-well plate (sterile, black, flat-bottom, polystyrene microplate from Brand #7816668) and the fluorescence was followed for 3 minutes to obtain values for background fluorescence. After obtaining a baseline, 2 µL $Disc_3(5)$ dissolved in DMSO was added to each well to a final concentration of 1 µM $Disc_3(5)$ and 1% DMSO, and the fluorescence intensity was measured for another 15 minutes. At this point, 20 µL of stock solutions of receptors **R3**–**R4** and control antibiotics (dissolved to 10× the desired concentration in Müller-Hinton broth with 1% DMSO) were added and the fluorescence was measured for 1 hour. All fluorescence measurements were preformed using a BioTek Cytation 5 Cell Imaging Multi-Mode Reader (35 °C, excitation at 610 nm and emission at 660 nm, time intervals of 23 seconds, orbital shaking (5 seconds at 548 rpm (2 mm) before each measurement). As a positive control, 10 µM gramicidin was used which is known to cause membrane depolarization.[16] As a negative control,

[15] J. D. te Winkel, D. A. Gray, K. H. Seistrup, L. W. Hamoen and H. Strahl, *Front. Cell Dev. Biol.*, **2016**, *4.* DOI: 10.3389/fcell.2016.00029.
[16] D. A. Kelkar and A. Chattopadhyay, *Biochim. Biophys. Acta, Biomembr.*, **2007**, *1768*, 2011–2025.

the ribosome-targeting antibiotic clindamycin was used (2 µg/mL), as well as a blank run (no antibiotic added). The kinetic traces is shown in Figure S35, and a bar graph showing the fluorescence intensity after 10 minutes incubation with **R3**, **R4** or control compounds is shown in the main manuscript. The lipid binder **R4** clearly shows depolarization of the membrane, while the chloride transporter **R3** does not. Chloride is not the main contributor to membrane potential, which may explain the lack of depolarizing activity of **R3**.

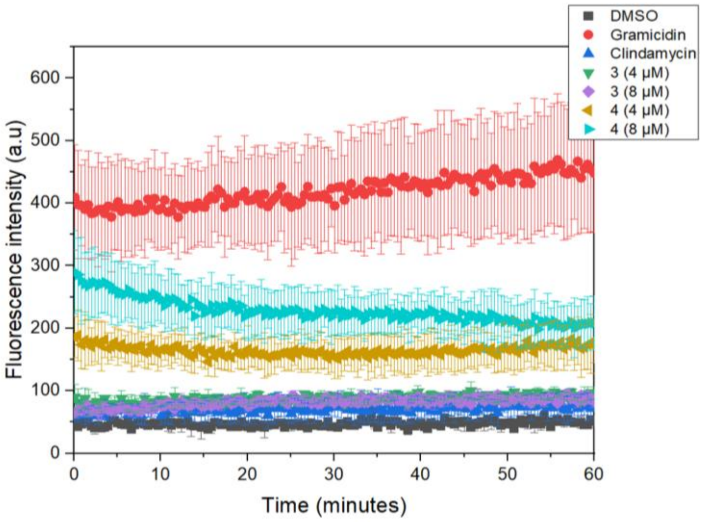

Figure S39. Membrane depolarization of *B. subtilis* induced by **R3** and **R4**. Fluorescence intensity of Disc$_3$(5) in *B. subtilis* after incubation with DMSO (1%, blank), clindamycin (2 µg/mL, negative control), gramicidin (10 µM, positive control), 4 µM **R3**, 8 µM **R3**, 4 µM **R4**, or 8 µM **R4**. Results are the average of at least 2 technical x 2 biological repeats and error bars represent standard deviations.

5.5 MQAE chloride influx assay

MQAE chloride influx assay was performed by a modification of the previously published method.[17] For each experiment, a small amount of the *B. subtilis* glycerol stock was streaked onto a Müller Hinton agar plate and the agar plate was incubated for 24 hours at 35°C. The obtained colonies were aseptically transferred into sterile cation-adjusted Müller-Hinton broth and diluted to OD_{600} of 0.2. The bacteria were subsequently incubated at 35 °C until they reached mid-logarithmic phase (OD_{600} of 0.7). The cultures were centrifuged at 3000 rpm for 5 mins, washed with phosphate-buffered saline (PBS), and resuspended in PBS to obtain OD_{600} of 0.2. The bacteria were treated with *N*-(ethoxycarbonylmethyl)-6-methoxyquinolium bromide (MQAE) to achieve a final MQAE concentration of 10 mM. The bacteria containing the dye were incubated for 1 h at 37°C, centrifuged at 3000 rpm for 5 minutes, washed with PBS to remove the dye, and resuspended in PBS to obtain OD_{600} of 0.2. Then, 200 µL of the bacteria suspension were transferred into a 96-well plate (sterile, black, flat-bottom, polystyrene microplate from Brand #7816668), and the fluorescence intensity was measured (λ_{ex} = 350 nm, λ_{em} = 460 nm, 25 °C, 10 s linear shaking prior to measuring fluorescence). Subsequently samples were treated with compounds **R3**–**R4** and controls (final DMSO concentration 1%), and the fluorescence was measured after 5 min incubation.

[17] L. E. Brennan, L. K. Kumawat, M. E. Piatek, A. J. Kinross, D. A. McNaughton, L. Marchetti, C. Geraghty, C. Wynne, H. Tong, O. N. Kavanagh, F. O'Sullivan, C. S. Hawes, P. A. Gale, K. Kavanagh and R. B.P. Elmes., *Chem*, **2023**, *9*, 3138–3158.

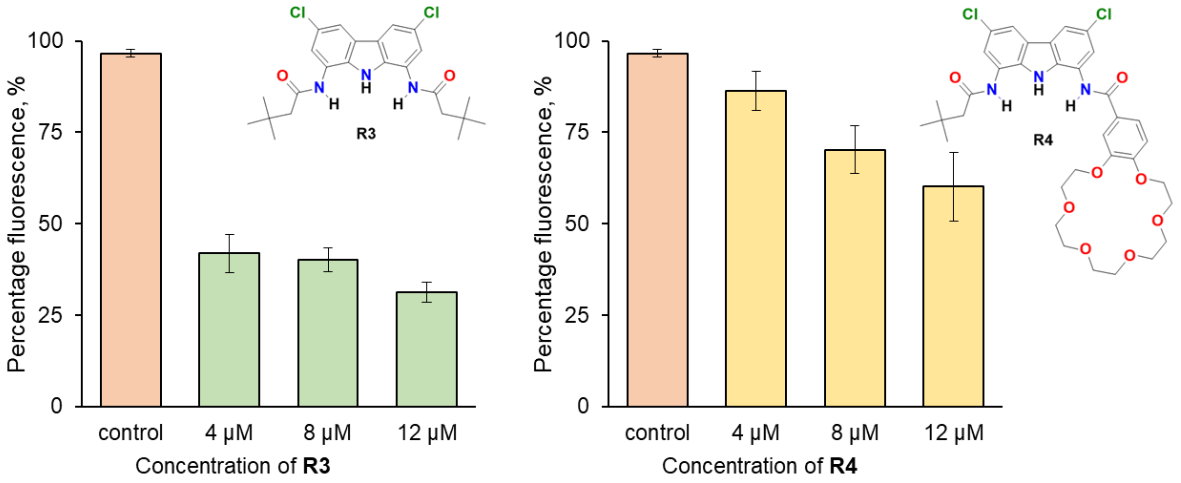

Figure S40. Percentage fluorescence of MQAE in *B. subtilis* in the absence (*control*) or presence of **R3** and **R4** at various concentrations. Results are the average of at least 2 technical x 2 biological repeats and error bars represent standard deviations.

5.6 Hemolytic activity of **R1–R4**

Hemolysis is the destruction of erythrocytes/red blood cells (RBCs) resulting in the release of hemoglobin from the RBC into the blood plasma. The following protocol was adapted from a protocol designed for antimicrobial peptides.[18] Single donor human red blood cells were washed twice and diluted in phosphate-buffered saline (PBS) to obtain a final concentration of 2×10^7 cells/mL. The assay was performed in a 96 well plate with a final volume of 200 µl and different concentrations of our receptors. First, 8 µl of receptor in DMSO was added to each well, diluted with 192 µl of the red blood cell suspension and mixed by pipetting (final DMSO concentration – 4%). A detergent (1% Triton X-100) was used as a positive control and 4% DMSO in PBS buffer was used as a negative control. The plates were sealed with a protective film to prevent evaporation and incubated for 1 hour at 37˚C in a VWR 1585 Incubator (no shaking). After incubation, the 96 well plates were centrifuged at 3900 rpm for 5 min. 50 µl of each well's supernatant was then carefully transferred to a new 96 well plate, which was then centrifuged to remove any bubbles, and the absorbance was measured at 414 nm. The percent haemolysis was calculated by the following formula:

$$\%\,Haemolysis\ = \frac{Abs_{sample} - Abs_{neg}}{Abs_{pos} - Abs_{neg}} \cdot 100\%$$

where Abs_{sample} is each sample's absorption at 414 nm, Abs_{neg} is the negative control's average absorbance at 414 nm, and Abs_{pos} is the positive control's average absorption at 414 nm. The percent of haemolysis values were input into OriginPro and fitted with a sigmoidal model with the maximum value set at 100% to determine HC_{50} from the inflection point (Figures S41–S44).

[18] A. Oddo and P. R. Hansen in Antimicrobial Peptides: Methods and Protocols (Ed.: P. R. Hansen), Springer New York, NY, **2017**, 427–435.

Hemolytic activity of R1

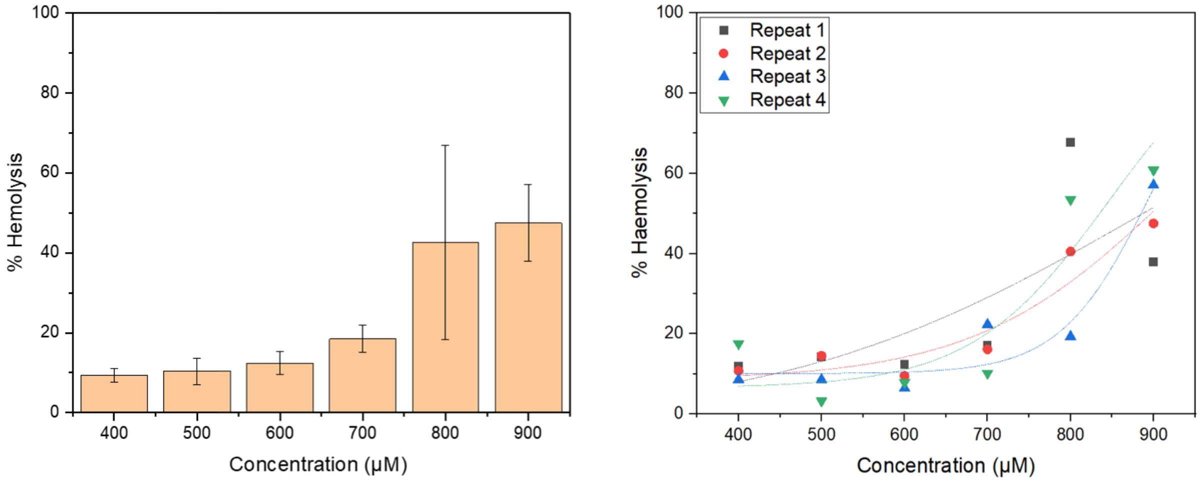

Model	DoseResp			
Equation	y = A1 + (A2-A1)/(1 + 10^((LOGx0-x)*p))			
Plot	Repeat 1	Repeat 2	Repeat 3	Repeat 4
A1	-2.03081 ± 64.85509	8.41322 ± 6.43718	10.05556 ± 3.76641	6.54706 ± 9.38715
A2	100 ± 0	100 ± 0	100 ± 0	100 ± 0
LOGx0	878.63248 ± 260.19124	918.70498 ± 34.47966	897.0999 ± 16.1532	846.62908 ± 38.24002
p	0.00201 ± 0.00371	0.00368 ± 0.00164	0.008 ± 0.00307	0.00523 ± 0.00278
Reduced Chi-Sqr	395.0294	41.3751	44.39158	153.99622
R-Square (COD)	0.52379	0.90872	0.92701	0.85276
Adj. R-Square	0.20631	0.84787	0.87835	0.75461

Figure S41. Normalized hemolytic activity of receptor **R1** at varying concentrations. The results are the average of a minimum 2 biological × 2 technical repeats and the error bars represent standard deviations (left). HC_{50} calculations using the adjusted DoseResp model. The dashed lines represent the fit of the model (right). Table: HC_{50} output chart with inflection value (Logx0).

Hemolytic activity of R2

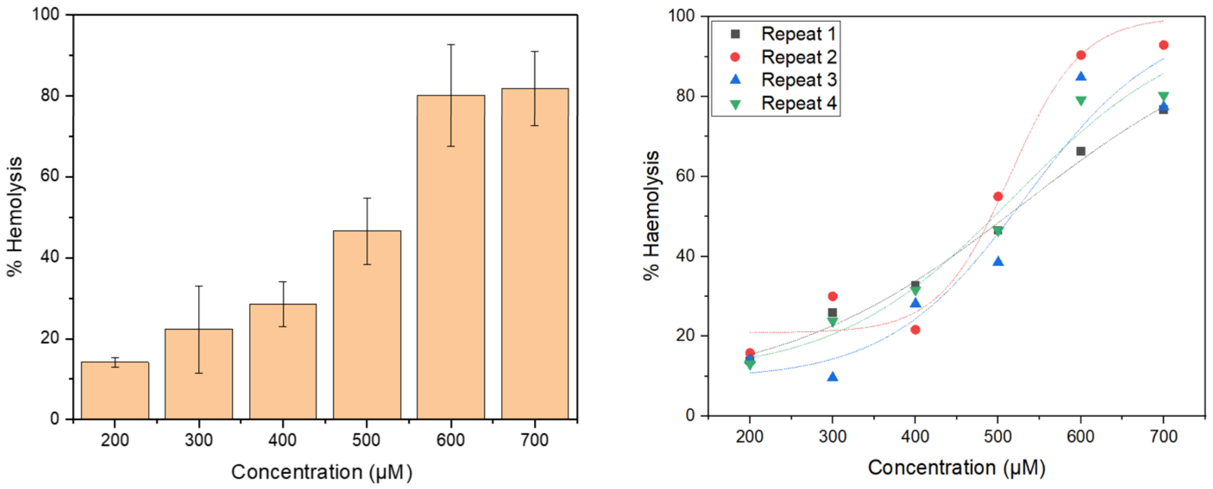

Model	DoseResp			
Equation	y = A1 + (A2-A1)/(1 + 10^((LOGx0-x)*p))			
Plot	Repeat 1	Repeat 2	Repeat 3	Repeat 4
A1	6.4293 ± 6.57316	20.86537 ± 4.96614	9.20375 ± 11.5985	10.19622 ± 9.55256
A2	100 ± 0	100 ± 0	100 ± 0	100 ± 0
LOGx0	530.26533 ± 27.47676	514.57978 ± 16.25258	532.30679 ± 41.75462	519.64175 ± 36.62607
p	0.00294 ± 4.42492E-4	0.01009 ± 0.00356	0.00527 ± 0.00227	0.00403 ± 0.00112
Reduced Chi-Sqr	8.0466	52.2132	134.67206	43.38967
R-Square (COD)	0.9918	0.97329	0.92107	0.96799
Adj. R-Square	0.98634	0.95549	0.86844	0.94664

Figure S42. Normalized hemolytic activity of receptor **R2** at varying concentrations. The results are the average of a minimum 2 biological × 2 technical repeats and the error bars represent standard deviations (left). HC_{50} calculations using the adjusted DoseResp model. The dashed lines represent the fit of the model (right). Table: HC_{50} output chart with inflection value (Logx0).

Hemolytic activity of R3

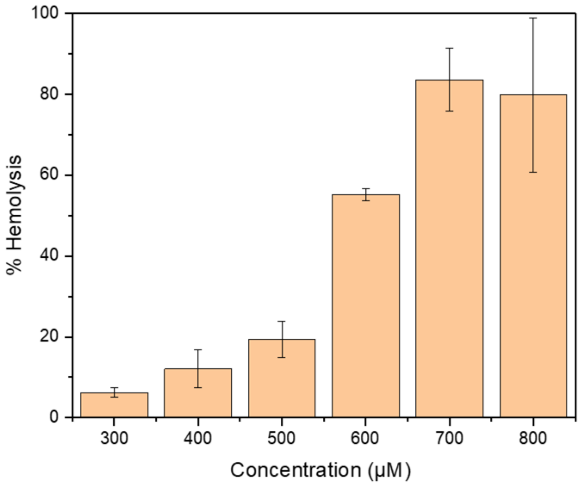

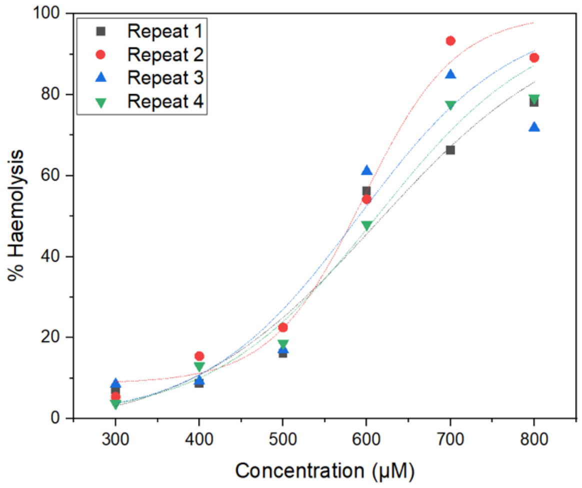

Model	DoseResp			
Equation	y = A1 + (A2-A1)/(1 + 10^((LOGx0-x)*p))			
Plot	Repeat 1	Repeat 2	Repeat 3	Repeat 4
A1	-3.3249 ± 14.63841	8.69089 ± 5.61086	-0.2853 ± 19.08919	-0.26131 ± 9.22294
A2	100 ± 0	100 ± 0	100 ± 0	100 ± 0
LOGx0	612.01952 ± 49.55073	595.54825 ± 16.76947	589.90337 ± 59.87073	611.61178 ± 30.47238
p	0.00377 ± 0.00128	0.00789 ± 0.00209	0.00475 ± 0.00242	0.00444 ± 0.00112
Reduced Chi-Sqr	78.66236	45.90948	206.97805	49.27153
R-Square (COD)	0.95308	0.98114	0.89486	0.97324
Adj. R-Square	0.9218	0.96857	0.82476	0.95539

Figure S43. Normalized hemolytic activity of receptor **R3** at varying concentrations. The results are the average of a minimum 2 biological × 2 technical repeats and the error bars represent standard deviations (left). HC_{50} calculations using the adjusted DoseResp model. The dashed lines represent the fit of the model (right). Table: HC_{50} output chart with inflection value (Logx0).

Hemolytic activity of R4

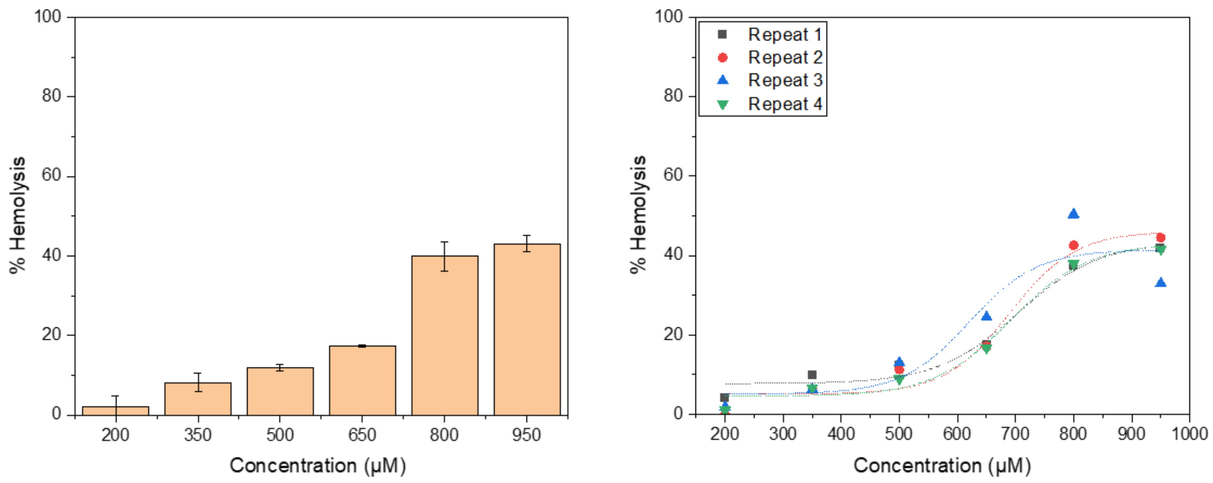

Figure S44. Normalized hemolytic activity of receptor **R4** at varying concentrations. The results are the average of a minimum 2 biological × 2 technical repeats and the error bars represent standard deviations (left). HC_{50} calculations using the adjusted DoseResp model. The dashed lines represent the fit of the model (right). Because the data levels off at 45% hemolysis (presumably because higher concentrations are not soluble anymore) an accurate estimate of HC_{50} is not possible. However, it is clear that to achieve 50% haemolysis (HC_{50}), a concentration >950 μM will be necessary.

6 *In silico* studies

Methyl phosphoethanolamine and methyl phosphorylcholine were chosen for conformation search as model headgroups of palmitoyl-2-oleoyl-*sn*-glycero-3-phosphoethanolamine (POPE) and palmitoyl-2-oleoyl-*sn*-glycero-3-phosphocholine (POPC), respectively.

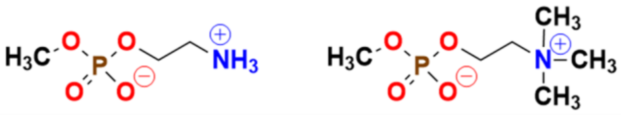

Methyl phosphoethanolamine   Methyl phosphorylcholine

The geometry of **R4**:methyl phosphoethanolamine and **R4**:methyl phosphorylcholine complexes was optimized *in silico* starting from the receptor conformation in which amide groups were in a *syn*-*syn* conformation. The geometry optimization and vibrational frequencies calculation were done by Gaussian 16 v. C.01 package using wB97XD functional and 6-31++ G(d,p) basis set.[19]

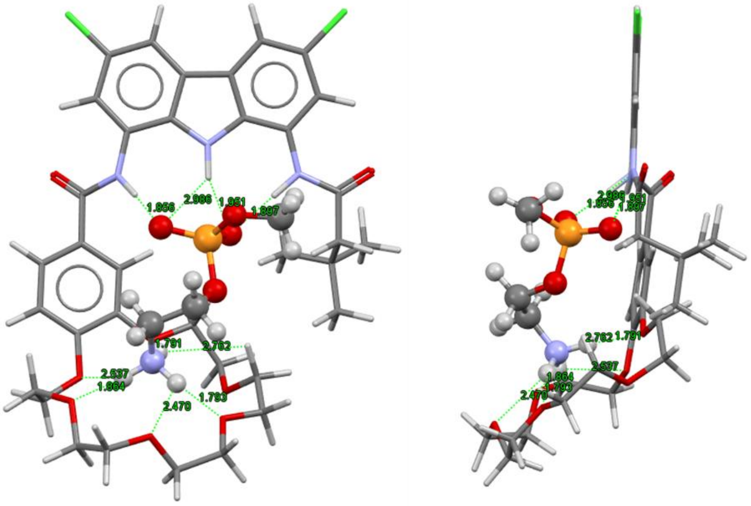

Figure S45. The lowest energy structure of **R4**:methyl phosphoethanolamine complex.

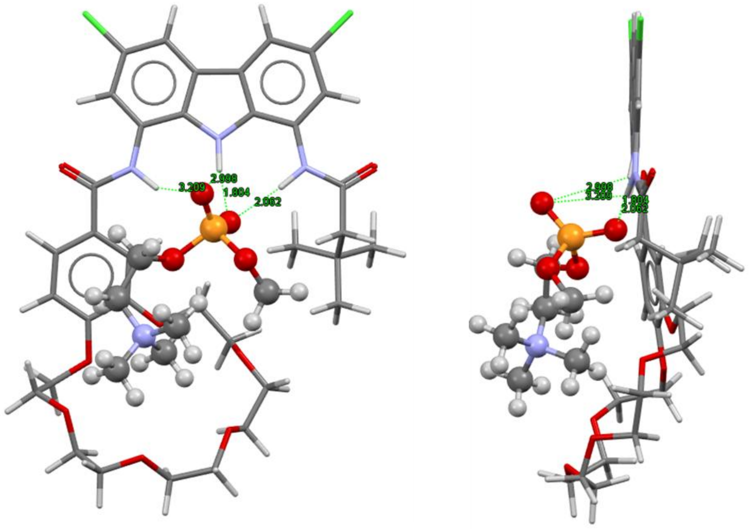

Figure S46. The lowest energy structure of **R4**:methyl phosphorylcholine complex.

[19] B. Zawada and M. J. Chmielewski, *Org. Biomol. Chem.*, **2024**, *22*, 7143-7147.

Table S2. Atomic coordinates of the optimized structure of **R4** and methyl phosphoethanolamine shown in Figure S45.

Atom number	Atom type	x	y	z
1	C1	12.863	-6.8768	5.3158
1	C	12.961	-7.1460	5.2970
2	C	13.266	-6.5420	6.5250
3	C	12.296	-6.0440	7.3770
4	C	10.963	-6.1700	6.9780
5	C	10.643	-6.7900	5.7550
6	C	11.638	-7.2730	4.8940
7	C	9.7040	-5.8030	7.5900
8	C	8.6950	-6.2240	6.7040
9	N	9.2740	-6.8320	5.6070
10	C	9.3770	-5.1750	8.7960
11	C	8.0350	-4.9910	9.0650
12	C	7.0140	-5.4010	8.1940
13	C	7.3320	-6.0280	6.9920
14	N	11.241	-7.8530	3.6690
15	N	6.3820	-6.4720	6.0550
16	Cl	7.5670	-4.2080	10.565
17	Cl	14.955	-6.4100	6.9860
18	C	11.863	-8.9040	3.0630
19	O	12.890	-9.4400	3.4570
20	C	11.171	-9.3830	1.8170
21	C	5.0250	-6.5550	6.2180
22	O	4.4310	-6.2050	7.2290
23	C	4.2890	-7.1470	5.0280
24	C	4.0780	-8.6830	5.1060
25	C	3.3700	-9.0810	6.4070
26	C	3.1930	-9.0710	3.9130
27	C	5.4230	-9.4160	4.9950
28	C	9.7770	-9.4510	1.7300
29	C	11.352	-10.160	-0.4620
30	C	11.950	-9.7710	0.7360
31	C	9.9500	-10.666	-2.9250
32	O	9.3310	-8.4170	-3.7010
33	C	10.401	-9.3300	-3.5040
34	O	6.7790	-7.0410	-4.1840
35	C	7.7720	-7.3420	-5.1460
36	C	8.5380	-8.6230	-4.8580
37	O	4.9790	-7.6060	-2.1100
38	C	4.5370	-7.1880	-3.3850
39	C	5.5290	-7.6590	-4.4240
40	C	9.9660	-10.193	-0.5690
41	C	9.1790	-9.8740	0.5550
42	O	7.8290	-9.9620	0.3580
43	C	6.9360	-9.8780	1.4700
44	C	5.5820	-10.333	0.9530
45	O	5.1220	-9.5950	-0.1630

46	C	4.5560	-8.3360	0.1330
47	C	3.9580	-7.7800	-1.1390
48	H	13.752	-7.5300	4.6680
49	H	12.566	-5.5810	8.3190
50	H	10.139	-4.8480	9.4930
51	H	5.9780	-5.2430	8.4550
52	H	3.3120	-6.6550	5.0020
53	H	4.8230	-6.9180	4.0970
54	H	3.1680	-10.158	6.4100
55	H	3.9780	-8.8440	7.2850
56	H	2.4180	-8.5510	6.5170
57	H	3.0460	-10.156	3.8700
58	H	3.6560	-8.7500	2.9740
59	H	2.2060	-8.5990	3.9820
60	H	5.9760	-9.0880	4.1080
61	H	6.0580	-9.2230	5.8650
62	H	5.2630	-10.499	4.9360
63	H	9.1640	-9.1560	2.5750
64	H	11.982	-10.433	-1.3000
65	H	13.030	-9.7510	0.8290
66	H	9.2290	-11.152	-3.5880
67	H	10.811	-11.339	-2.8300
68	H	11.086	-8.8260	-2.8200
69	H	10.929	-9.5050	-4.4520
70	H	7.3250	-7.4080	-6.1490
71	H	8.4740	-6.5040	-5.1360
72	H	7.8540	-9.4720	-4.7160
73	H	9.1850	-8.8460	-5.7190
74	H	3.5600	-7.6350	-3.6130
75	H	4.4370	-6.0940	-3.4130
76	H	5.1570	-7.3850	-5.4230
77	H	5.6140	-8.7540	-4.3700
78	H	6.9150	-8.8580	1.8700
79	H	7.2620	-10.556	2.2680
80	H	4.8580	-10.297	1.7790
81	H	5.6610	-11.366	0.6040
82	H	5.3010	-7.6460	0.5500
83	H	3.7400	-8.4380	0.8640
84	H	3.2060	-8.4790	-1.5310
85	H	3.4710	-6.8180	-0.9220
86	H	8.7690	-7.1560	4.7860
87	H	6.7340	-6.7570	5.1400
88	H	10.554	-7.3280	3.1110
89	O	9.2630	-10.518	-1.6990
90	P	8.0110	-6.1920	2.3740
91	O	7.0740	-6.4100	1.0220
92	O	7.7330	-4.6390	2.7240
93	C	7.4540	-5.7180	-0.1570
94	C	8.3740	-6.5650	-1.0140
95	N	7.6990	-7.8190	-1.4380

96	H	6.7430	-7.6460	-1.7890
97	H	8.2390	-8.2520	-2.2000
98	H	7.6140	-8.5190	-0.6810
99	H	7.9560	-4.7740	0.0820
100	H	6.5310	-5.4890	-0.7020
101	H	9.2730	-6.8280	-0.4520
102	H	8.6480	-6.0230	-1.9220
103	O	9.4550	-6.3240	2.0020
104	O	7.3970	-7.1170	3.3990
105	C	6.4600	-4.2410	3.2280
106	H	6.3220	-4.5960	4.2530
107	H	6.4490	-3.1510	3.2190
108	H	5.6480	-4.6200	2.5960

Table S3. Atomic coordinates of the optimized structure of **R4** and methyl phosphorylcholine shown in Figure S46.

Atom number	Atom type	x	y	z
1	C1	12.863	-6.8768	5.3158
2	C2	13.180	-6.4393	6.6101
3	C3	12.225	-6.162	7.5686
4	C4	10.886	-6.3258	7.2023
5	C5	10.547	-6.7667	5.9093
6	C6	11.532	-7.054	4.9509
7	C7	9.6368	-6.133	7.9073
8	C8	8.6181	-6.4595	6.9963
9	N9	9.1766	-6.8791	5.8052
10	C10	9.3220	-5.7214	9.2063
11	C11	7.9821	-5.6429	9.5321
12	C12	6.9512	-5.9428	8.6284
13	C13	7.2588	-6.3534	7.3348
14	N14	11.1124	-7.5264	3.6885
15	N15	6.3021	-6.6474	6.3458
16	Cl16	7.5292	-5.1361	11.1516
17	Cl17	14.8774	-6.2411	7.0143
18	C18	11.8858	-8.1109	2.7258
19	O19	13.1076	-8.1689	2.7744
20	C20	11.1487	-8.704	1.5555
21	C21	4.9625	-6.8614	6.5266
22	O22	4.4011	-6.8123	7.6133
23	C23	4.2042	-7.1902	5.2521
24	C24	4.0175	-8.7074	4.9889
25	C25	3.2254	-9.3671	6.1249
26	C26	3.2288	-8.8485	3.6782
27	C27	5.3837	-9.3929	4.8371
28	C28	9.7967	-9.0665	1.5928
29	C29	11.2883	-9.5209	-0.7138
30	C30	11.8874	-8.9418	0.4032
31	C31	9.9349	-10.8026	-2.8941

32	O32	9.0059	-8.8961	-4.1453
33	C33	10.1733	-9.6236	-3.8320
34	O34	6.1039	-8.1419	-4.5075
35	C35	7.0459	-8.5783	-5.4655
36	C36	8.0612	-9.58	-4.9418
37	O37	4.1445	-8.2177	-2.1684
38	C38	3.9095	-8.3146	-3.5554
39	C39	5.0046	-9.0171	-4.3401
40	C40	9.9405	-9.8683	-0.6877
41	C41	9.1868	-9.6323	0.4815
42	O42	7.8825	-9.9864	0.4134
43	C43	7.0804	-9.8495	1.5794
44	C44	5.6523	-10.1084	1.1713
45	O45	5.1537	-8.9444	0.5402
46	C46	3.8391	-9.0593	0.0462
47	C47	3.7948	-9.3783	-1.4359
48	H54	13.6477	-7.09	4.6056
49	H55	12.5072	-5.8279	8.5604
50	H56	10.0905	-5.4734	9.9293
51	H57	5.9178	-5.8643	8.9332
52	H58	3.2201	-6.7216	5.3496
53	H59	4.7116	-6.7439	4.3912
54	H60	3.0753	-10.4318	5.9109
55	H61	3.7450	-9.2768	7.0820
56	H62	2.2422	-8.8988	6.2414
57	H63	3.0414	-9.9060	3.4543
58	H64	3.7787	-8.4132	2.8365
59	H65	2.2574	-8.3458	3.7466
60	H66	5.9995	-8.8841	4.0868
61	H67	5.9421	-9.3833	5.7784
62	H68	5.2535	-10.4412	4.5418
63	H69	9.2059	-8.9029	2.4859
64	H70	11.8958	-9.7076	-1.5909
65	H71	12.9411	-8.6868	0.3966
66	H72	9.2934	-11.5484	-3.3707
67	H73	10.8914	-11.2931	-2.6711
68	H74	10.8483	-8.8949	-3.3772
69	H75	10.6504	-10.0026	-4.7503
70	H76	6.5258	-9.0155	-6.3312
71	H77	7.5802	-7.6866	-5.8071
72	H78	7.5596	-10.3708	-4.3668
73	H79	8.5676	-10.0504	-5.7998
74	H80	2.9561	-8.8334	-3.7393
75	H81	3.8160	-7.2907	-3.9291
76	H82	4.6002	-9.2917	-5.3273
77	H83	5.3107	-9.9433	-3.8324
78	H84	7.1639	-8.8426	2.0071
79	H85	7.3965	-10.5803	2.3358
80	H86	5.0565	-10.332	2.0666
81	H87	5.6138	-10.9745	0.4947

82	H88	3.3412	-8.0979	0.2144
83	H89	3.2824	-9.8279	0.6007
84	H90	4.4789	-10.2104	-1.6600
85	H91	2.7730	-9.6893	-1.7058
86	H97	8.6468	-6.9638	4.9359
87	H98	6.6414	-6.7193	5.3883
88	H100	10.1284	-7.4204	3.4835
89	O101	9.2512	-10.4404	-1.7136
90	P48	7.7836	-5.7579	2.6518
91	O49	8.4402	-6.4463	1.2839
92	O50	6.3696	-5.1845	2.0694
93	C51	9.3486	-5.6854	0.5199
94	C52	9.3198	-6.1332	-0.9310
95	N53	7.9606	-6.0893	-1.6041
96	H92	10.3743	-5.8464	0.8754
97	H93	9.1461	-4.6141	0.6193
98	H94	9.6728	-7.1624	-1.0312
99	H95	9.9820	-5.4775	-1.5035
100	O96	8.6261	-4.6143	3.0737
101	O99	7.5108	-6.9636	3.5348
102	C102	7.1735	-4.8920	-1.1802
103	H103	6.2525	-4.8741	-1.7639
104	H104	6.9403	-4.9529	-0.1174
105	H105	7.7590	-3.9935	-1.3829
106	C106	8.1672	-6.0254	-3.0819
107	H107	8.7887	-6.8670	-3.3880
108	H108	8.6379	-5.0732	-3.3330
109	H109	7.1959	-6.1199	-3.5695
110	C110	7.1887	-7.3439	-1.2896
111	H111	7.7230	-8.1925	-1.719
112	H112	7.1210	-7.4452	-0.2115
113	H113	6.1964	-7.2734	-1.7355
114	C114	5.2838	-6.0579	1.7895
115	H115	4.3697	-5.5875	2.1605
116	H116	5.1956	-6.2201	0.7089
117	H117	5.4197	-7.0300	2.2671
